# Meet our North American editorial board members

**DOI:** 10.1111/iwj.70061

**Published:** 2024-10-07

**Authors:** Douglas Queen, Keith Harding

**Affiliations:** ^1^ International Wound Journal Oxford UK

In our recent editorial, we discussed the expansion of our Editorial Board^1^ and introduced you to our Senior Editorial Advisors. We promised a series of further editorials introducing our wider membership. This will be the first in a series of three providing insight into the group of outstanding and distinguished individuals that comprise our board. We have created the largest most internationally diverse board to greatly increase the capabilities and expertise of the journal as it moves to its third decade of life.

Look out for a series of editorials in the coming month introducing our new board members by providing some background on their valued experiences. As the editorial team, we are excited to expand this group to help maintain the high‐quality standards of the International Wound Journal moving forward into our third decade of existence.

## NORTH AMERICAN EDITORIAL BOARD MEMBERS

Please meet the North American Editorial Board Members for the International Wound Journal:

**Professor Afsaneh, Alavi, USA**

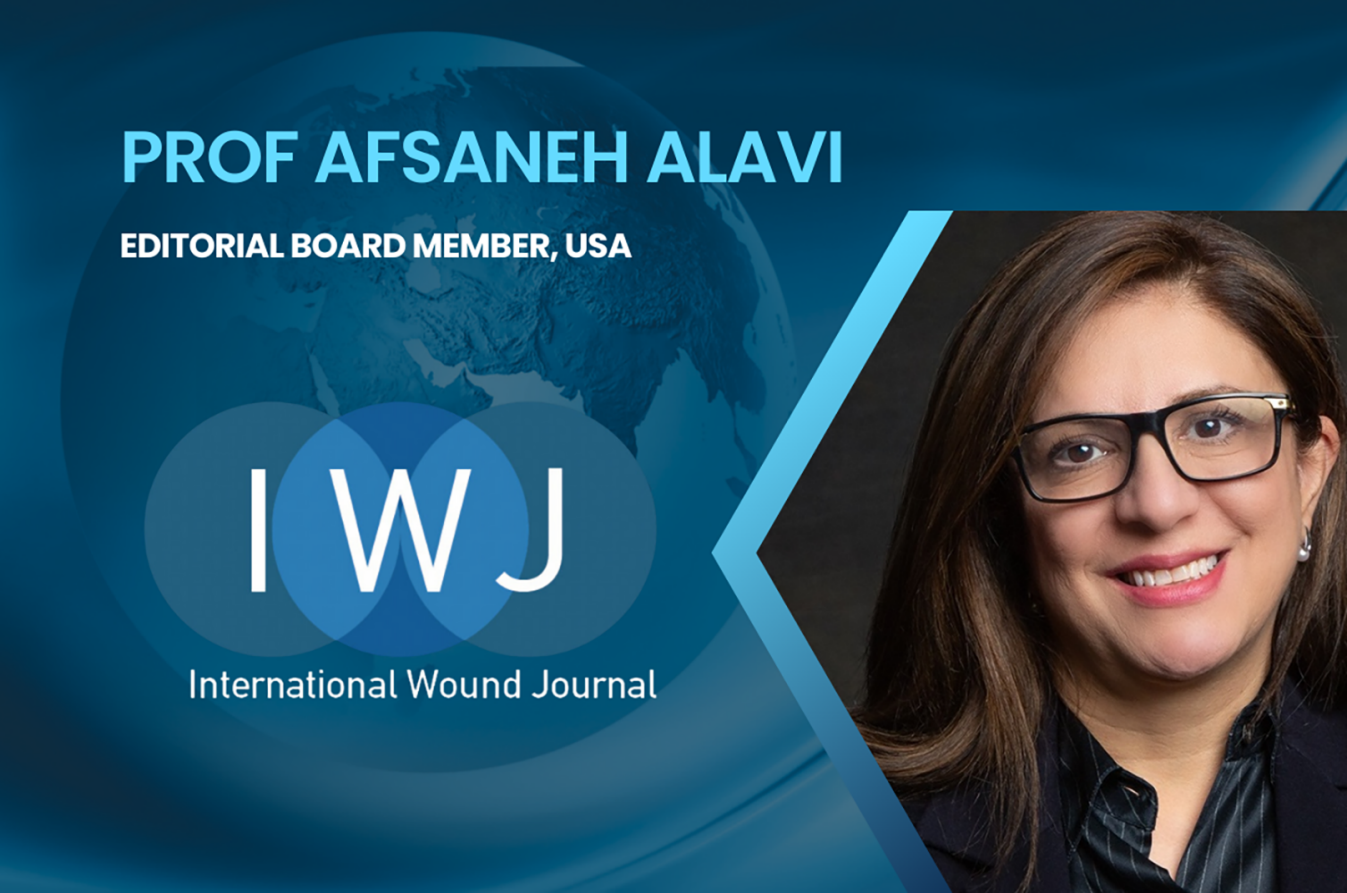

Afsaneh Alavi is a professor of Dermatology at the Mayo Clinic with special interests in inflammatory disorders and wound healing. Prior joining to the Mayo Clinic, Dr Alavi completed her residency in dermatology both in Iran and Canada and a 2‐year fellowship in wound healing at the University of Toronto in Canada. She earned her Master of Science in Community Health from the University of Toronto. She has received multiple prestigious awards including the Physician of the Year Award by the Canadian Dermatology Association in 2017 and the Supervisor of the Year Award by Mayo Clinic Dermatology in 2021.

Dr. Alavi has been involved in numerous clinical trials from phase I‐IV and basic science research. She is currently the Director of Complex Medical Dermatology Fellowship at the Mayo Clinic and runs the Hidradenitis Suppurativa Specialty Clinic.

**Dr David Alper, USA**

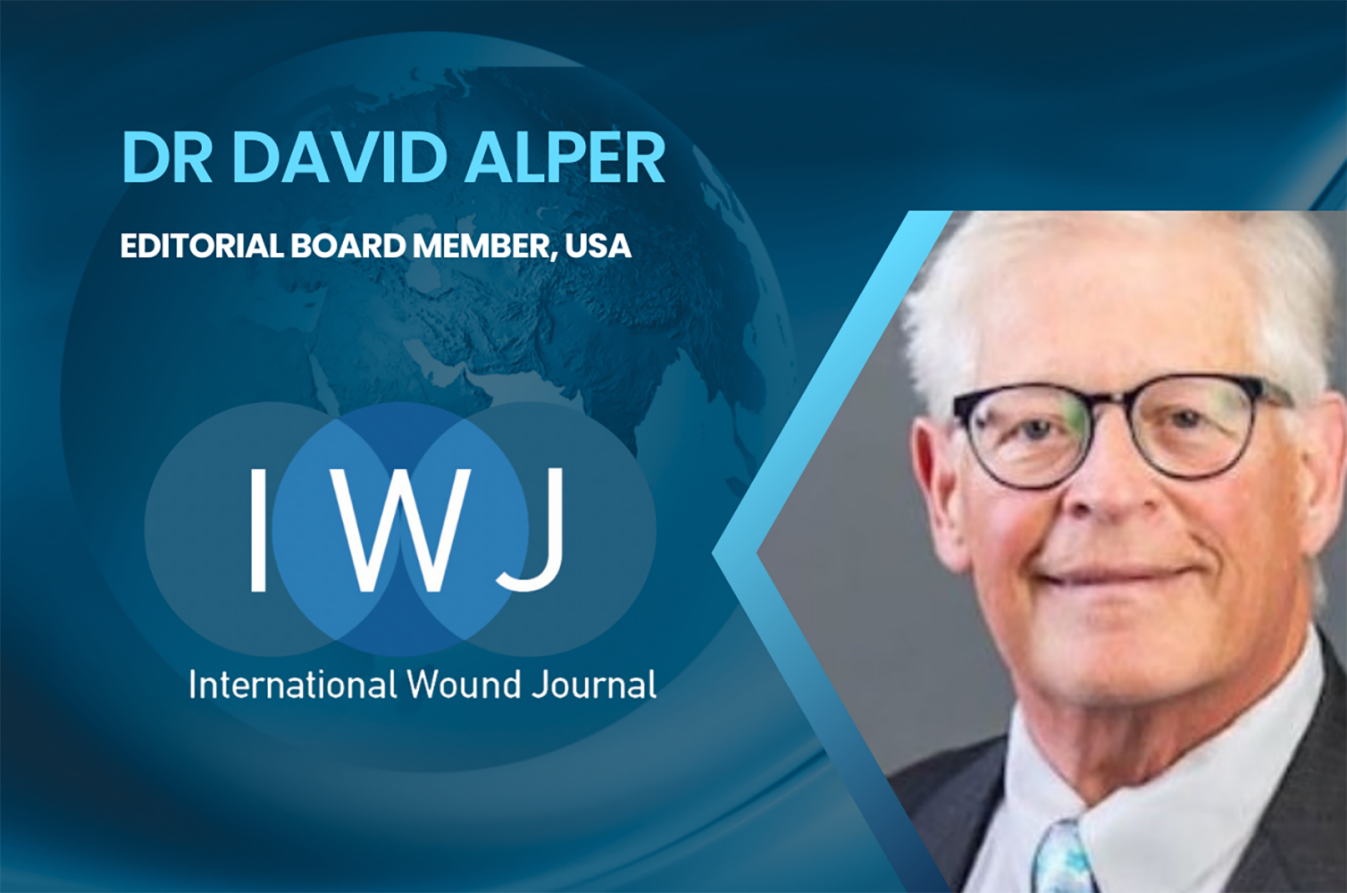

Dr. Alper is a podiatric physician who recently retired from working in the private solo practice he started 36 years ago in Belmont, MA. Specializing in preventative diabetic foot care, he is now Emeritus Surgical staff at Mt. Auburn Hospital (a Harvard teaching hospital). He is currently a member of the Leadership Board of the American Diabetes Association (ADA) in New England, having served as its President for over 20 years, and is an elected member of the Board of Trustees of the American Podiatric Medical Association (APMA). He also had the honour of serving as Chair of the Belmont (MA) Board of Health for 30 years, where he was seen as a leader in public health for the community. This led to his being appointed by the Governor to his current position on the Massachusetts Association of Health Boards (MAHB), allowing him to continue his public health work.

Since his retirement, Dr. Alper has spent much of his time in the world of limb amputation prevention, using his connections in diabetes, podiatry and vascular fields to create collaborations between medical societies. He currently serves on the Board of the Wound Care Collaborative Community (WCCC), The Way To My Heart (a patient advocacy organization) and the Foot and Ankle Health Section of the American Public Health Association (APHA).

**Professor Pravenn Arany, USA**

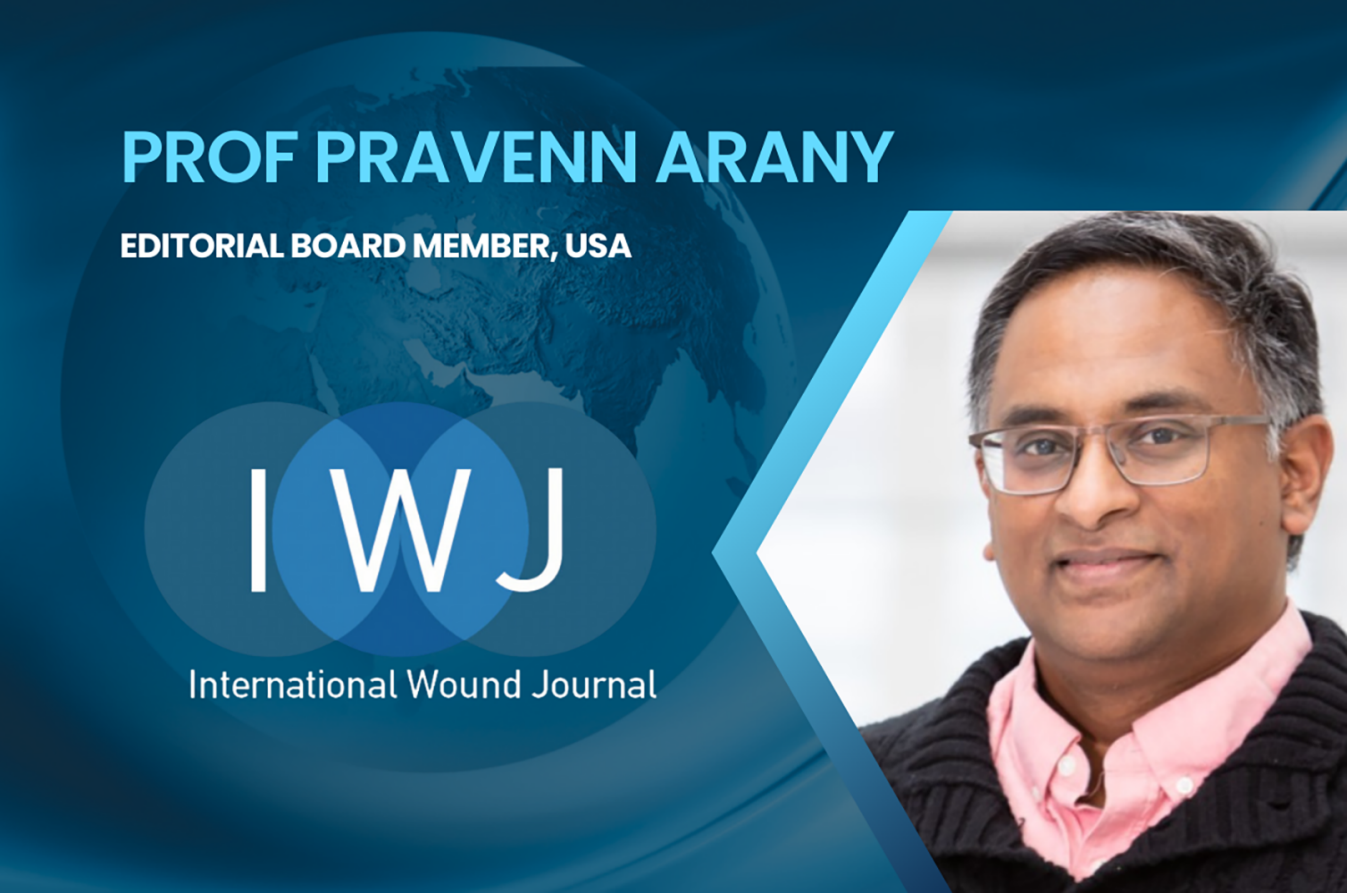

Dr. Arany trained as a dentist, oral pathologist and biomedical engineer. He served as an Assistant Clinical Investigator at NIDCR/NIH, Bethesda, from 2012 to 2015. He is currently an Associate Professor at the University at Buffalo, NY. He has six patents and over 150 scientific publications with more than 8000 citations and an h‐index of 36. His work has been featured in many mainstream media highlights in over 70 countries. He has received numerous awards recognizing his research contributions, including the Young Investigator Award from the National Institutes of Health and Wound Healing Society, the Horrace Furomoto Award from the American Society for Lasers in Surgery and Medicine and the Theodore Maiman Award from the Academy of Laser Dentistry. He has been invited to speak in various national and international forums, reviews for over 75 scientific journals, serves on nine journal editorial boards (including associate editor in four) and reviews grants for national and international funding agencies. He is the immediate past president of the World and North American Association for Photobiomodulation Therapy, Chair of the PBM group in SPIE and Optica (OSA) and Chair‐elect of Lasers and Biophotonics Group at the International Association of Dental Research.

**Professor Rummana Aslam, USA**

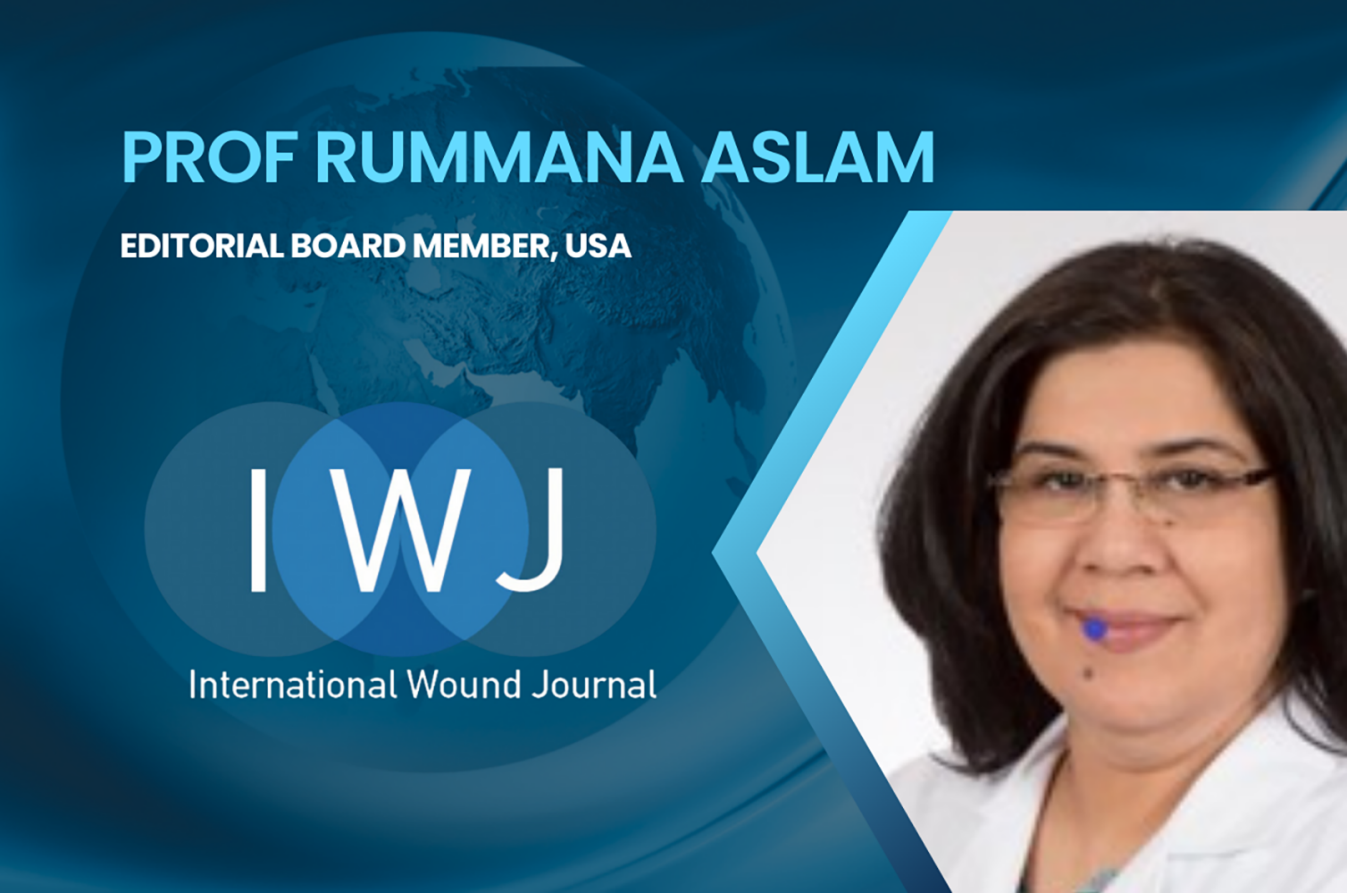

Dr Rummana Aslam is an Associate Professor and Chief of Physical Medical and Rehabilitation at Yale School of Medicine. She is also the Medical Director of Yale New Haven Health Wound and Hyperbaric Center. She founded the new ACGME‐accredited PMR residency programme at Yale and has initiated/designed the Yale Fellowship in Chronic Wound Care and Regenerative Medicine, where she serves as the Fellowship Program Director. She has a patent on a wound gel for chronic wounds issued in many countries. She has served on multiple panels and has co‐authored several guidelines on chronic wounds. Currently, she is leading a team of experts in developing guidelines for the management of lymphoedema. She is a board member of the Wound Healing Society Board of Directors. Her research in wound healing has focused on the biochemistry of oxygen and lactate in chronic wound healing, as well as on the wound healing properties of sugar.

Her clinical practice focuses on brain injury rehabilitation in addition to chronic wound care. She is the co ‐lead of the Yale Avascular Necrosis Program, which offers hyperbaric oxygen therapy in addition to core decompression surgery as a treatment for early avascular necrosis. She has a keen interest in Rehabilitation Engineering and is a principal investigator on multiple clinical trials at Yale, developing new innovative technologies to enhance mind and body performance.

**Professor Mona Baharestani, USA**

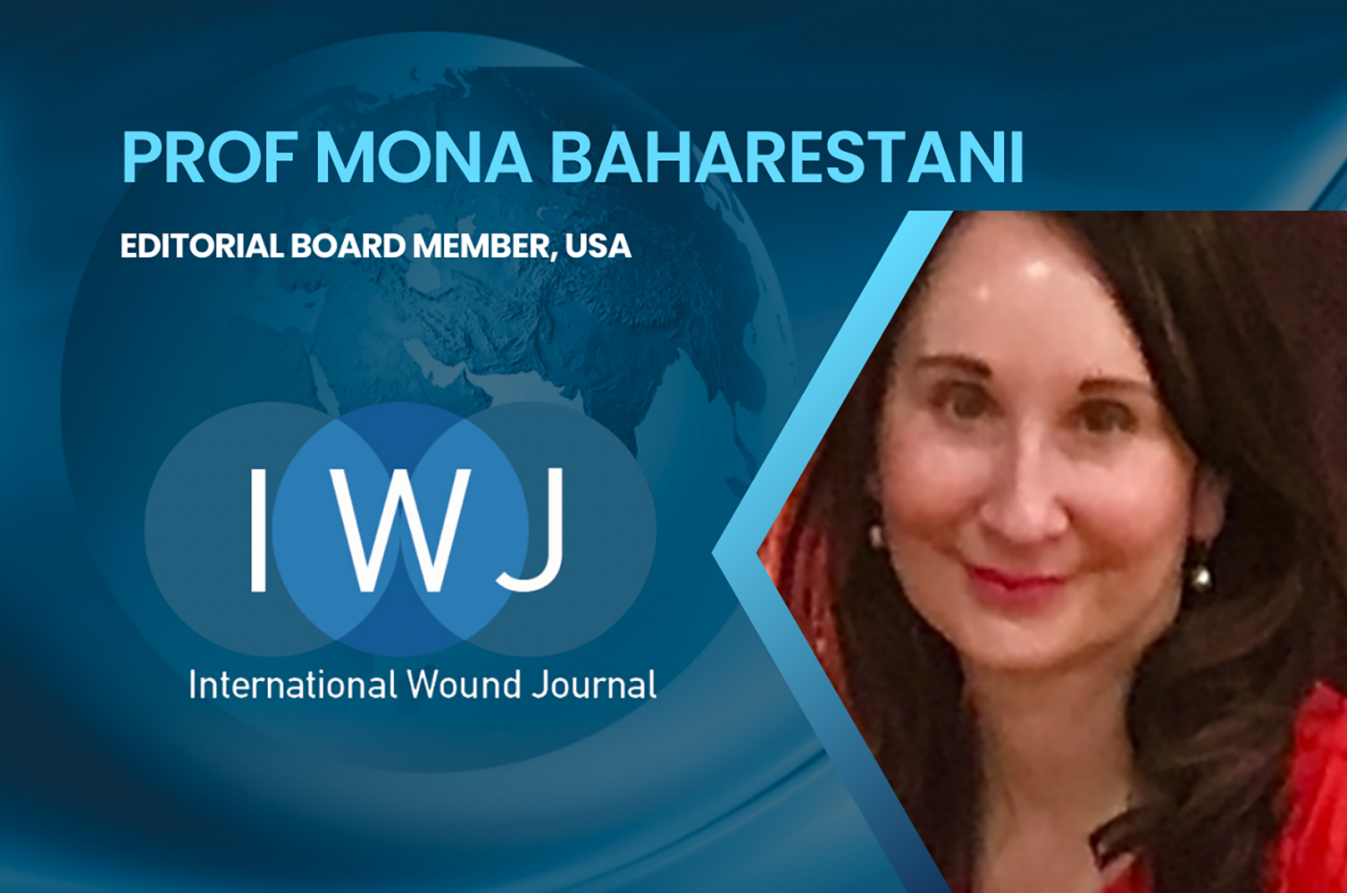

Dr. Mona Baharestani is a Clinical Associate Professor at the Quillen College of Medicine in Johnson City, Tennessee, and the Associate Chief of Wound Care & Research at the James H. Quillen VAMC. In her multi‐faceted role, she serves as a VHA pressure injury subject matter expert and directs health system‐wide wound care programmatic development in acute care, the Community Living Center (CLC), Home‐Based Primary Care (HBPC), and the Telewound programme. She provides interprofessional education, offers second opinion consultation, serves on multiple national pressure injury initiatives and performs clinical research. Selected as a Gold Status Fellow in the VA Diffusion of Excellence Program, she has mentored multiple VA healthcare systems in the development and advancement of TeleWound programmes aimed at increasing Veteran's access to wound care. This TeleWound Care Programme, ‘No Wound Left Behind Initiative’ (NWLB) was diffused across the Veteran's Health Administration (VHA) enterprise.

Prior to returning to her native home of Tennessee, Dr. Baharestani served as the Director of Wound Healing at the North Shore‐Long Island Jewish Healthcare System in New Hyde Park, New York. She also developed paediatric wound care guidelines which have been adopted worldwide.

**Professor Barbara Bates‐Jensen, USA**

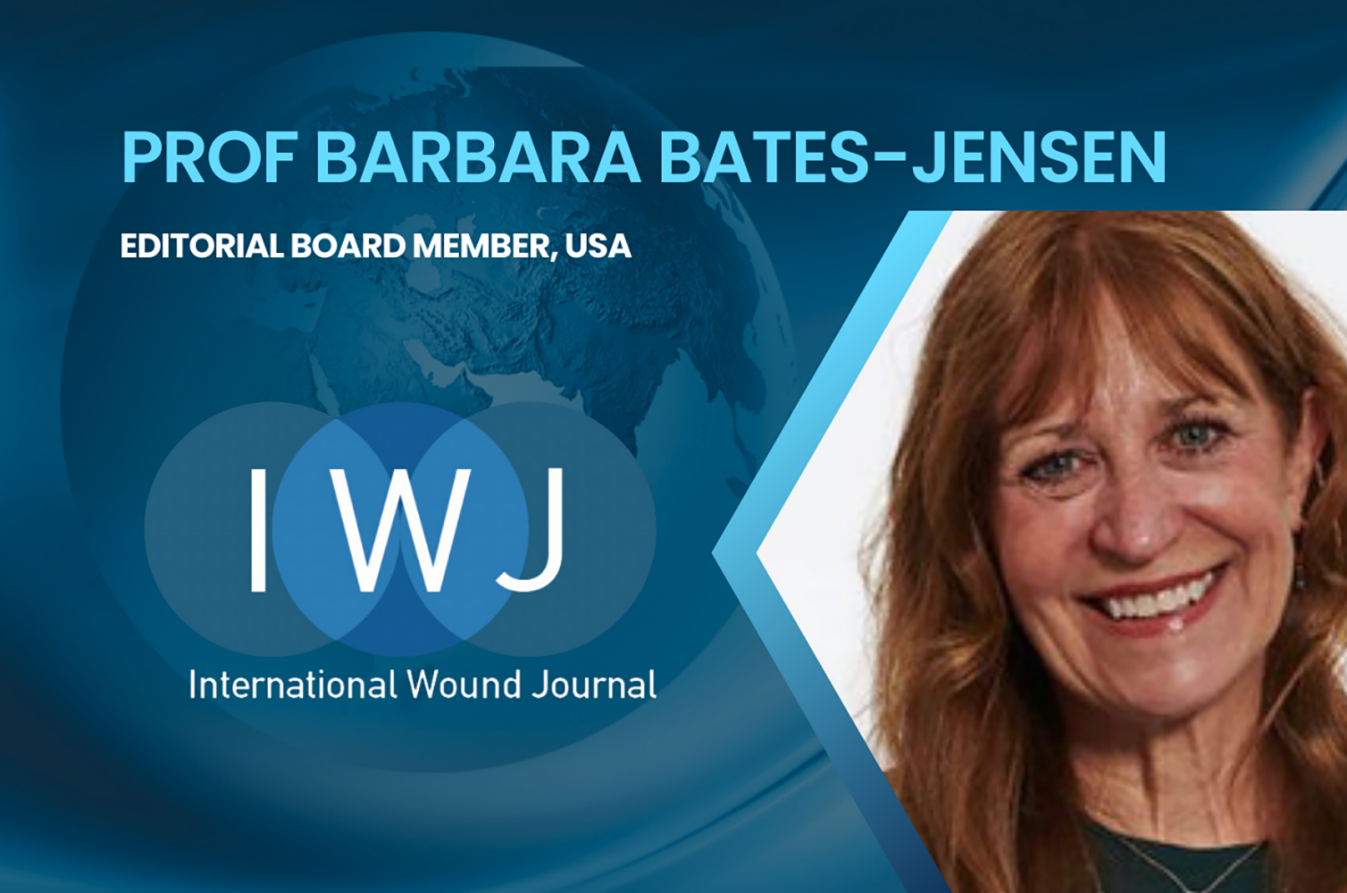

Dr. Barbara Bates‐Jensen is engaged in clinical research and education that highlights wound care best practices and focused on eliminating the disparities that exist in the detection of skin and tissue damage among persons of colour. Her research on pressure injuries and chronic wound care (screening, detection methods, assessment and management) focuses on vulnerable populations such as nursing home residents, elders, veterans with spinal cord injury and critically ill patients. Chronic wounds, such as ulcers, diabetic foot ulcers and leg ulcers have become a silent global epidemic.

Working with UCLA professors in computer science and bioengineering, Dr. Bates‐ Jensen helped invent a wound care medical device that measures skin and tissue damage before it becomes visible on the skin surface. Dr. Bates‐Jensen is the author and developer of the Bates‐Jensen Wound Assessment Tool (BWAT) a wound assessment tool that is used worldwide and incorporated into multiple EHRs. The research her team is doing has the potential to improve sustainable patient wound care throughout the global medical community.

**Professor Maryse Beaumier, Canada**

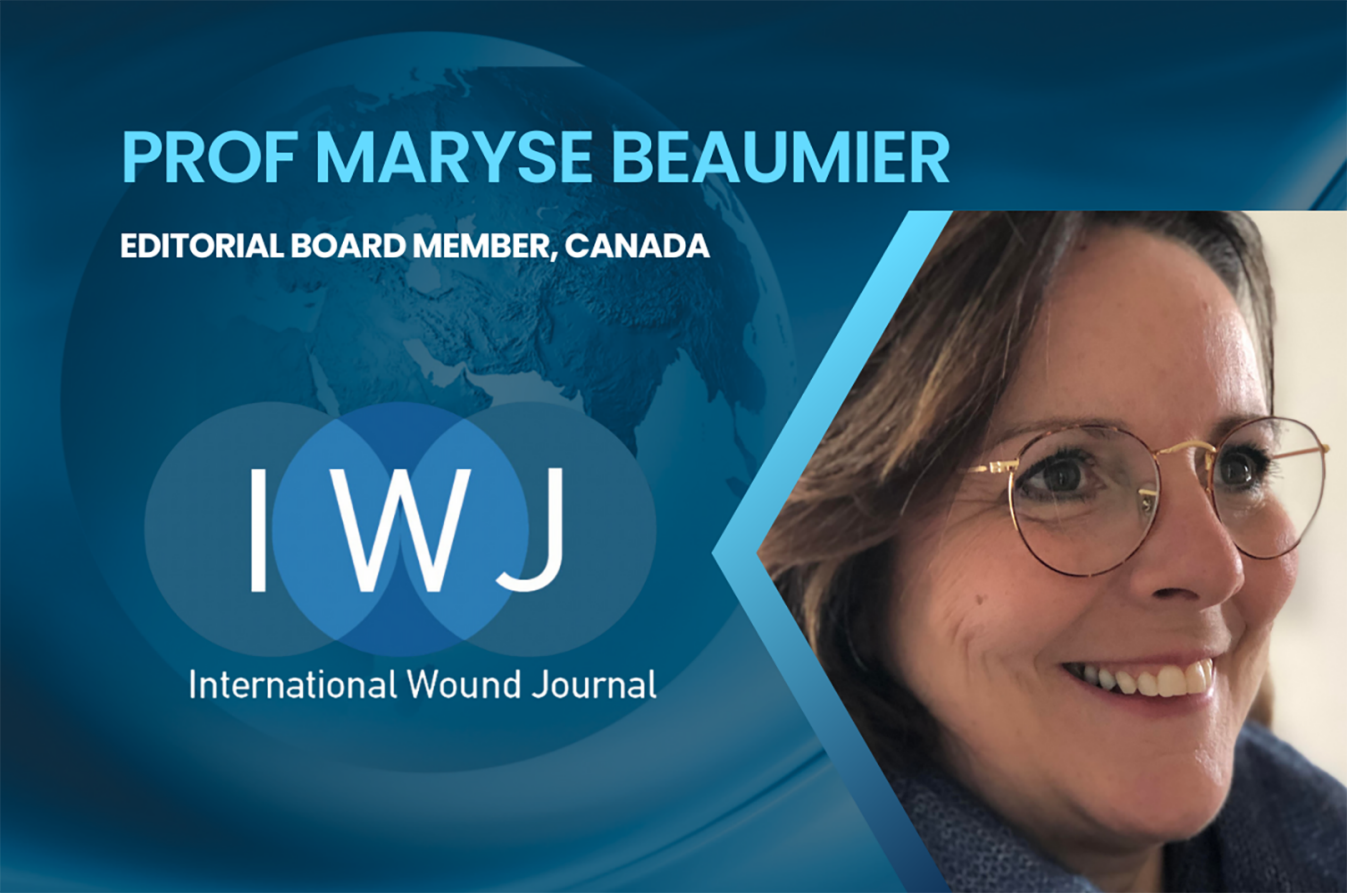

Professor in the Department of Health at the University of Quebec at Rimouski (UQAR), Campus Lévis, since August 2020, after 11 years in the Department of Nursing at the University of Quebec at Trois‐Rivières. She completed her Bachelor of nursing science at the University of Montreal and her Master's in nursing science at UQTR. She has completed a Ph.D. in biomedical sciences at the Faculty of Medicine at the University of Montreal (May 2016–January 2019) after an almost complete Ph.D. in Community Health at Laval University (2010–2015) (doctoral exams completed with success). Scientific director and researcher at the CISSS Chaudière‐Appalaches Research Center. Involved in the development of wound care for more than 20 years, author of Best Practice Recommendations for the Prevention and Management of Peripheral Arterial Ulcers, Director of the Board at the Canadian Association of Wound Care 2009–2012, member of the editorial board of Wound Care Canada 2010–2020 and for Limb Preservation in Canada de Wounds Canada since 2021, government advisor at the RQSP 2012–2015 and now member of the Groupe international de la francophonie en soins des plaies of Société française et francophone des plaies et cicatrisations (SFFPC) in France. Amputation's prevention, instrument development/validation, the organization of health services and legislative and ethical aspects support his research interests.

**Dr Dan Berlowitz, USA**

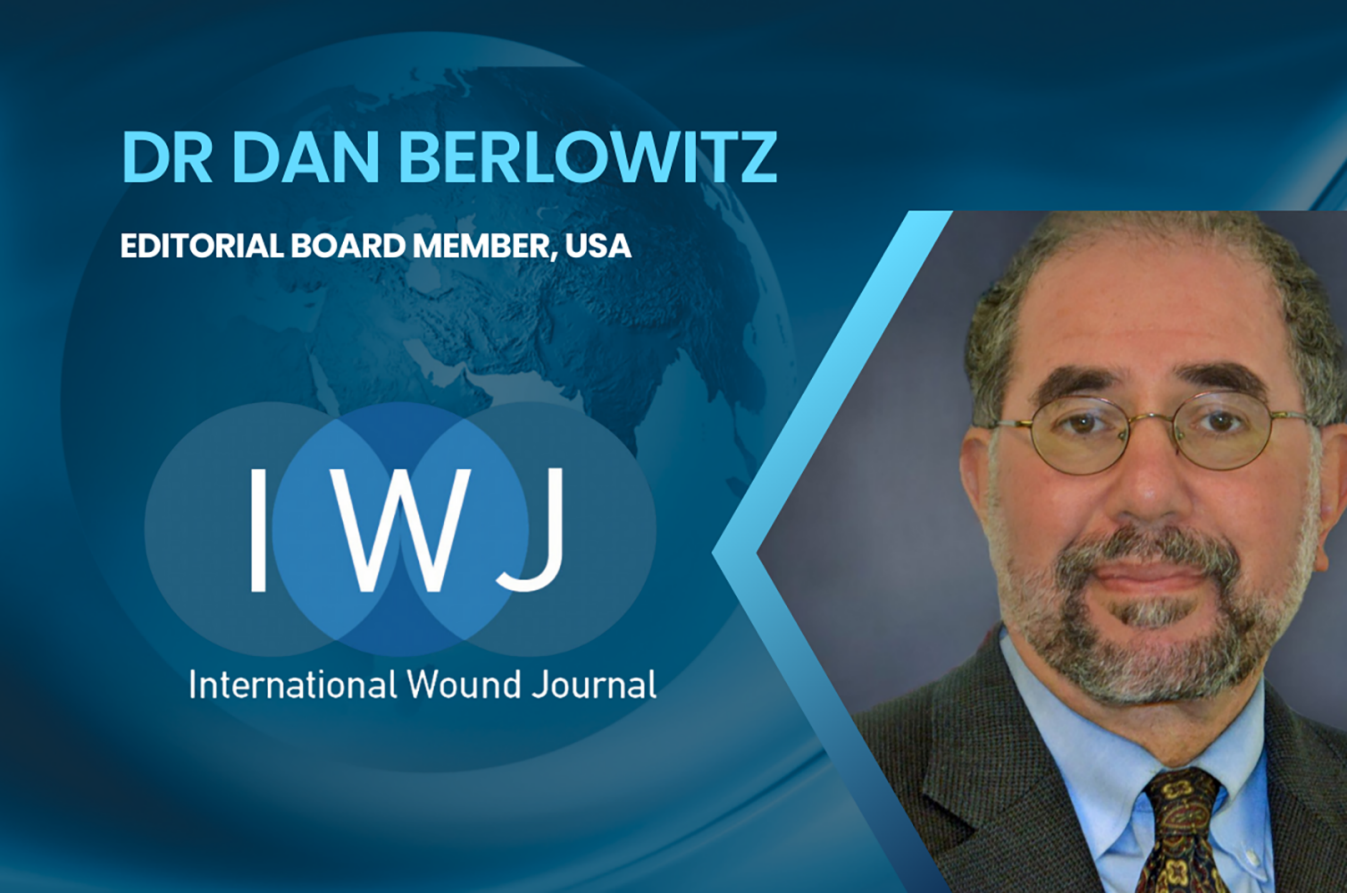

Dan Berlowitz, the chair of the Department of Public Health, has decades of experience as a physician, researcher, educator and administrator while serving at the Department of Veterans Affairs. His research interests centre on assessing and improving the quality of medical care in ambulatory and long‐term care settings. With strong methodological expertise in the areas of quality assessment, risk adjustment and the use of large databases, his research findings have led to improvements in uncontrolled hypertension and pressure injuries. A prolific author with more than 220 publications, Dr. Berlowitz's most recent article in the Journal of the American Geriatrics concluded that intensive blood pressure therapy is not associated with fear of falling among older hypertension patients.

**Professor Joyce Black, USA**

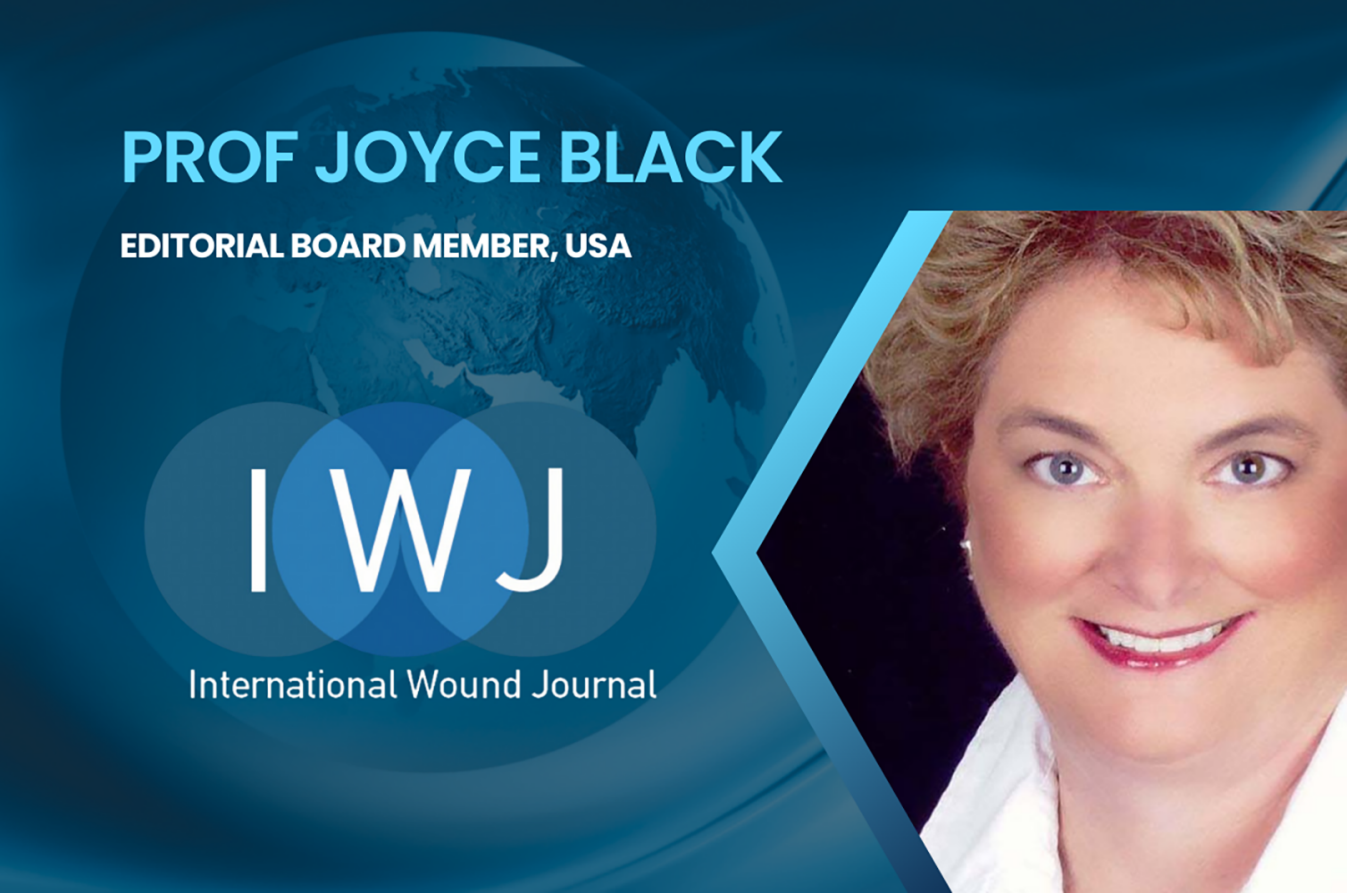

Joyce M. Black, PhD, RN is an Associate Professor in the College of Nursing at the University of Nebraska Medical Center in Omaha, Nebraska. She is a past president of the National Pressure Ulcer Advisory Panel. Dr Black served as the co‐chair of the task force to define deep tissue injury and as the chair of the task force to update the definitions of the stages of pressure ulcers.

**Professor Kath Bogie, USA**

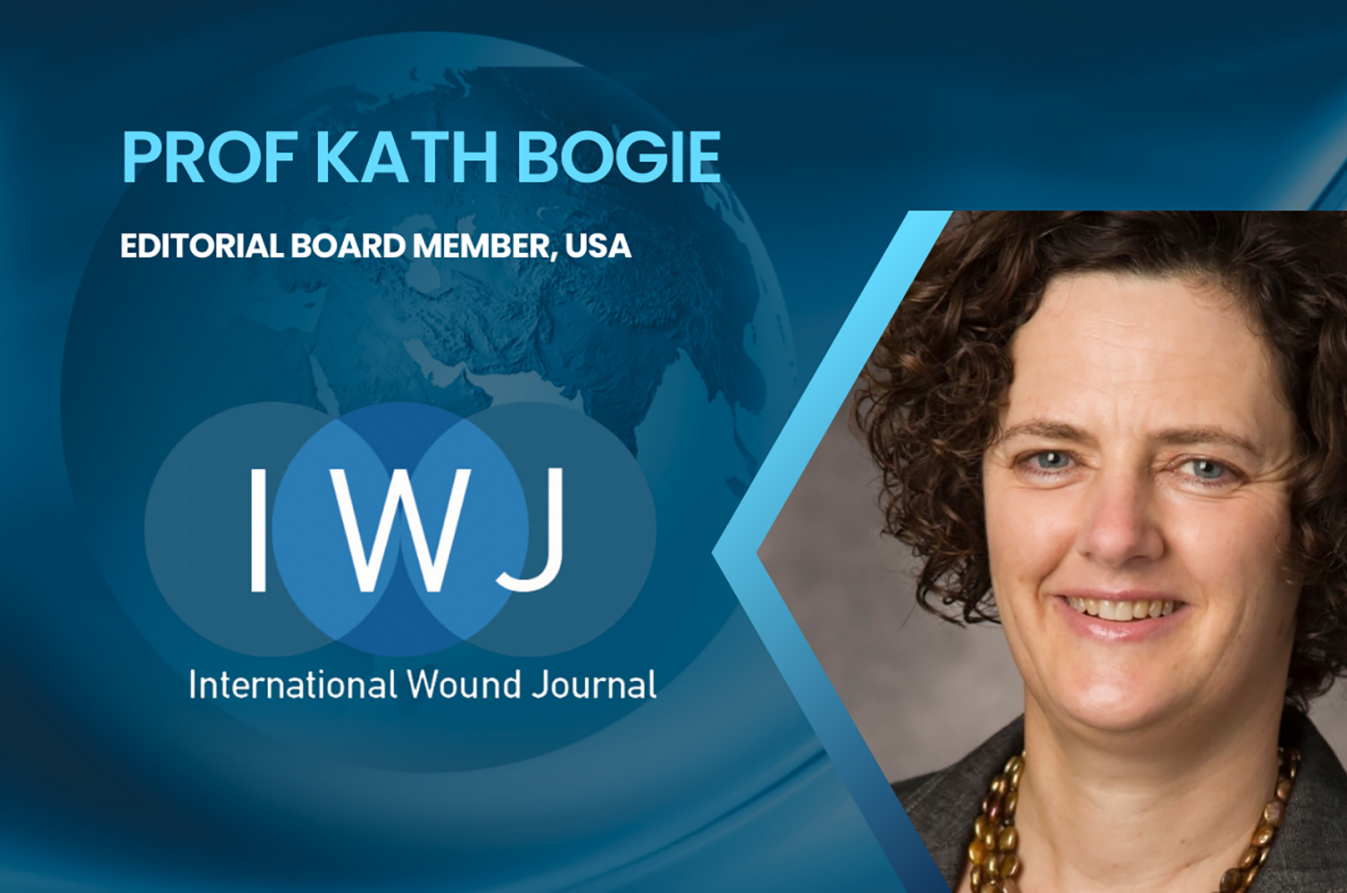

Professor Bogie is a Research Career Scientist at the Louis Stokes Cleveland VA Medical Center and a Professor in the Department of Orthopaedics at Case Western Reserve University, with an international reputation in translational research, leading interdisciplinary teams to develop patient‐centred interventions that meet the complex, multifactorial problems of wound management. Her contributions include evidence‐based preventive measures, personalized biomarkers and bioinformatics for better understanding of risk, complemented with smart and cost‐effective technology development.

Dr. Bogie is an elected Fellow of the American Institute for Medical and Biological Engineering, recognized for outstanding contributions to wound biotechnology development and patient‐centred pressure injury management. Dr. Bogie is the Editor‐in‐Chief for the Journal of Rehabilitation and Assistive Technologies Engineering and serves on the Board of Directors for the National Pressure Injury Advisory Panel, where she also co‐chairs the Research Committee and serves on the Standards Committee. She also currently chairs the Education Committee of the Wound Healing Society, having completed a 4‐year term on the Board of Directors. Dr. Bogie is co‐chair for the International Organization for Standardization microclimate standards development and serves on the RESNA Standards Committee on Wheelchair and Related Seating.

She has received continuous funding for over 20 years, from the DoD SCI Research Program, VA Rehabilitation Service, the Craig H. Neilsen Foundation and the Paralysed Veterans of America. She has published over 80 papers and chapters and holds 10 patents.

**Dr Scott Bolhack, USA**

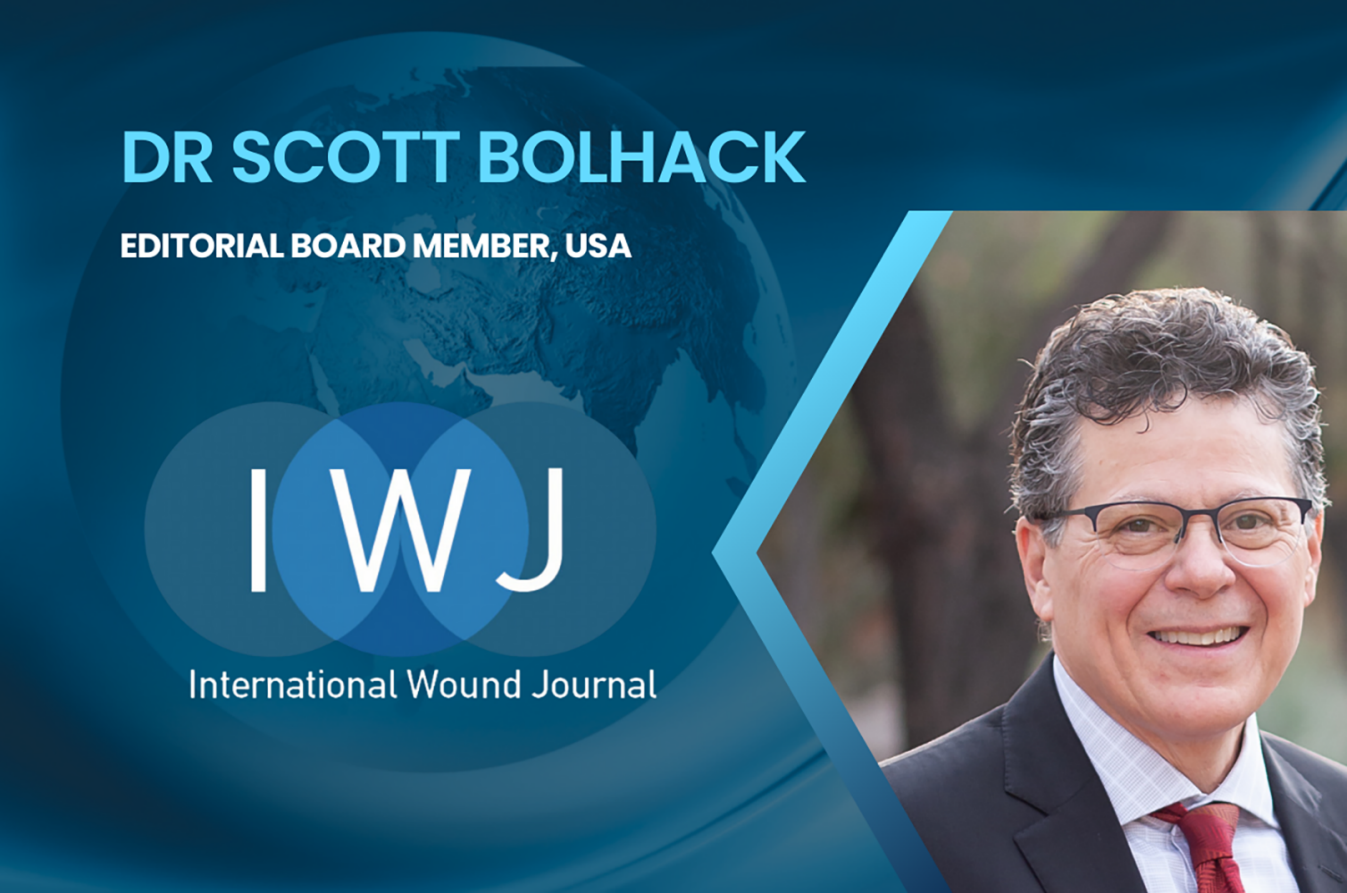

Dr. Bolhack is board‐certified in Internal Medicine and Hospice and Palliative Medicine, with additional credentials as a Certified Wound Specialist Physician and a Certified Medical Director in long‐term care. He has experience as a medical director for skilled nursing homes, assisted living facilities, hospices, home health agencies and wound centres. He has spoken on many topics nationally and has presented over 35 scientific posters in the areas of wound care, quality improvement and post‐hospital care.

**Dr Mariam Botros, Canada**

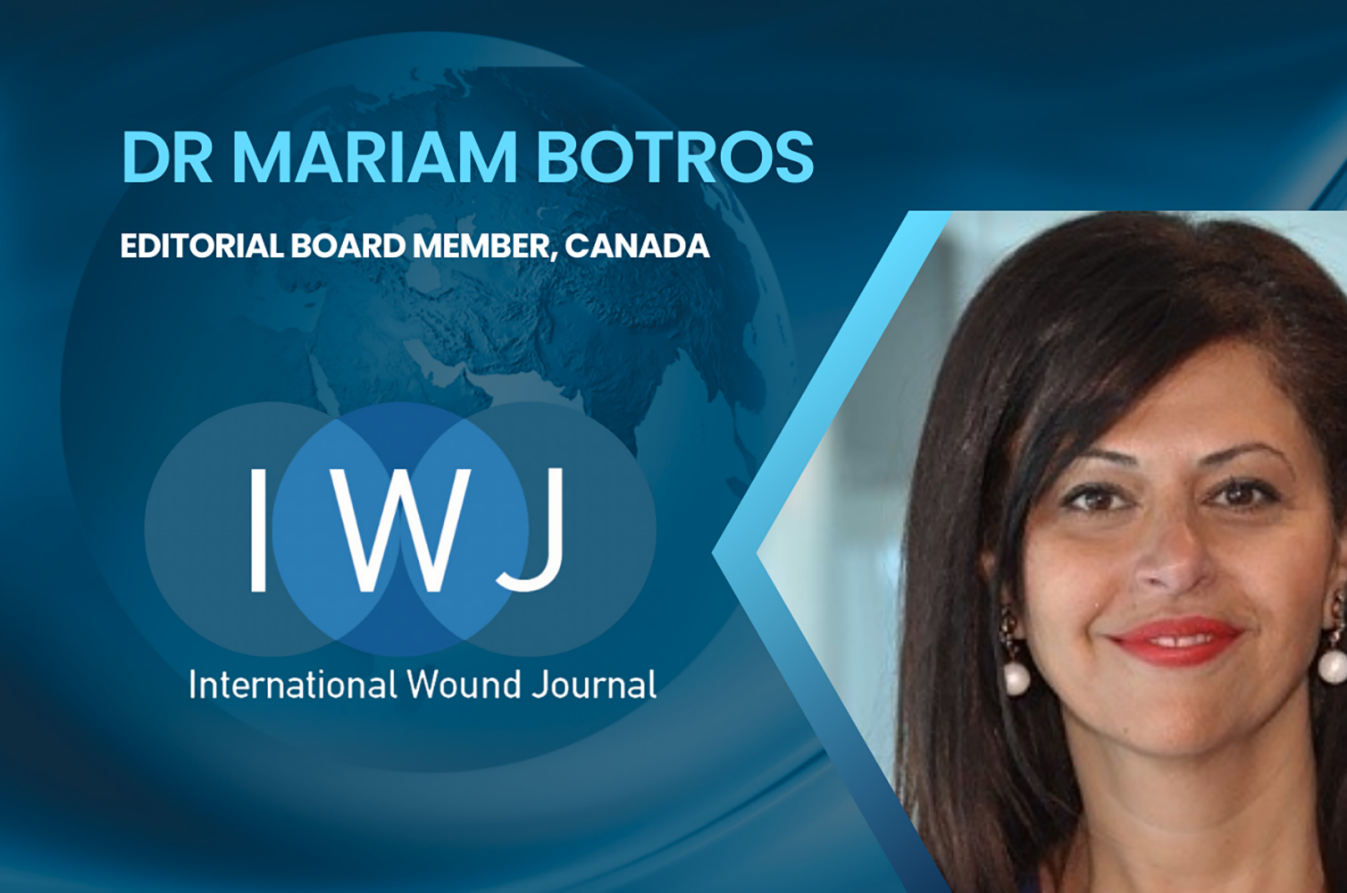

As chief executive officer of Wounds Canada, Mariam is responsible for both the implementation of the strategic direction and day‐to‐day operations of the organization. She is a chiropodist and diabetes educator by training and completed her Master's in Educational Leadership. Mariam has also published, developed and lectured in multiple programmes both nationally and internationally. She is also the vice president of D foot International. Through her different roles as an executive director, healthcare practitioner and educator, researcher and faculty member for many well‐recognized organizations, Mariam has extensive practical and professional experience in advancing public health policy and is respected as a strategic, visionary, system thinker.

**Professor David Brienza, USA**

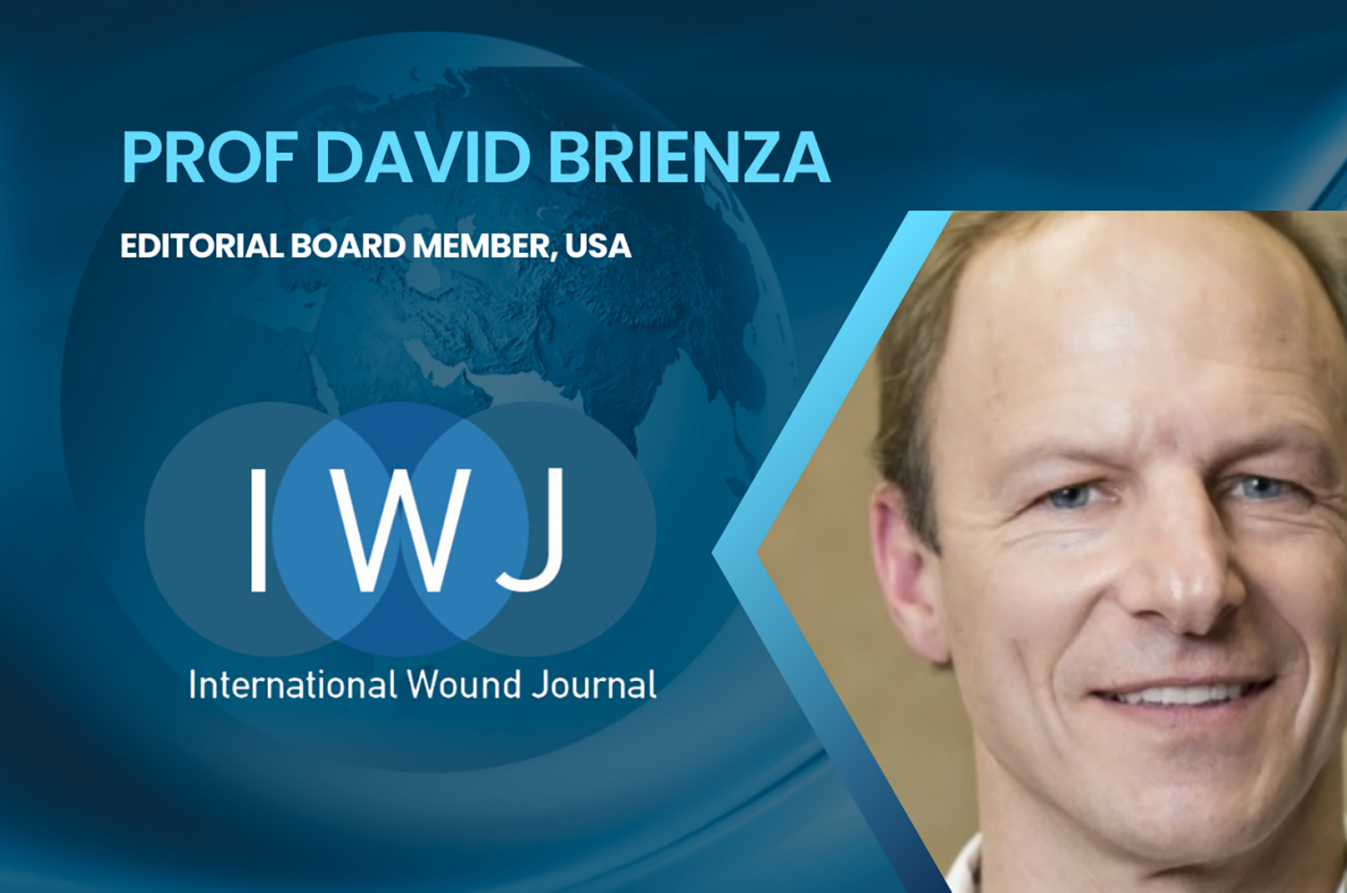

Associate Dean for Technology and Innovation at the School of Health and Rehabilitation Sciences (SHRS) and Professor in the Department of Rehabilitation Science and Technology (RST). Dave Brienza is an electrical engineer and has been investigating, developing and evaluating technology for mobility (wheelchairs) and tissue integrity management (seat cushions and mattresses) for the last 28 years. In addition to his role as Associate Dean for Research in the SHRS, he holds additional faulty appointments in the Department of Bioengineering and the McGowan Institute for Regenerative Medicine. He has served as the Director of the Rehabilitation Engineering Research Center (RERC) on Wheeled Mobility (2001–2004), Director of the RERC on Telerehabilitation (2004–2015) and Director of the RERC on Spinal Cord Injury (2007–2013). Brienza has an extensive record of federal funding and publication in the area of rehabilitation technology.

The scope of his work ranges from technology development in the seating and mobility to investigations on the physiological responses to mechanical and heat loading, to clinical effectiveness research in the form of large‐scale randomized controlled trials (RCTs). He is the inventor on seven patents related to technology for people with disabilities.

**Dr Karen Campbell, Canada**

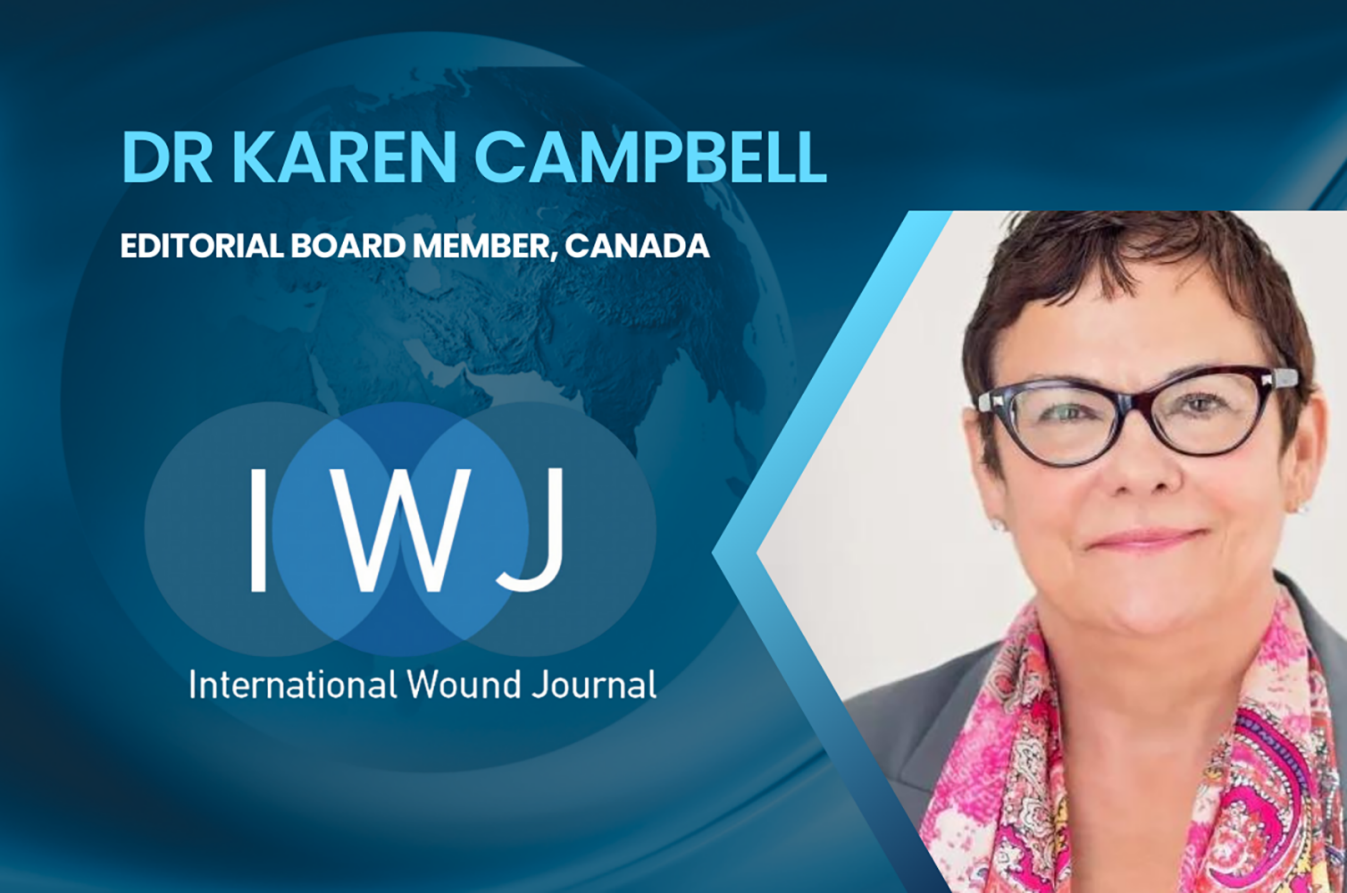

Dr Campbell is a nurse and current Field Leader for the Master of Clinical Science in Wound Healing at Western University in London, Ontario. As well, she is an Associate Scientist at the Lawson Health Research Institute and President‐Elect of the International Skin Tear Advisory Panel (ISTAP). She has functioned as an advanced practice nurse in wound care, continence and geriatrics.

Karen was the co‐chair of the RNAO's new Best Practice Guide (BPG) on Pressure Injuries and contributed to the Canadian BPG on Pressure Ulcer Prevention and Treatment in the spinal cord injury populations. She has been a panel member on the International Skin Tear Advisory Panel and Incontinence Associated Dermatitis International Best Practice Principles. Karen has a strong interest in skin health and frequently speaks and publishes on this topic.

**Dr Virginia Capasso, USA**

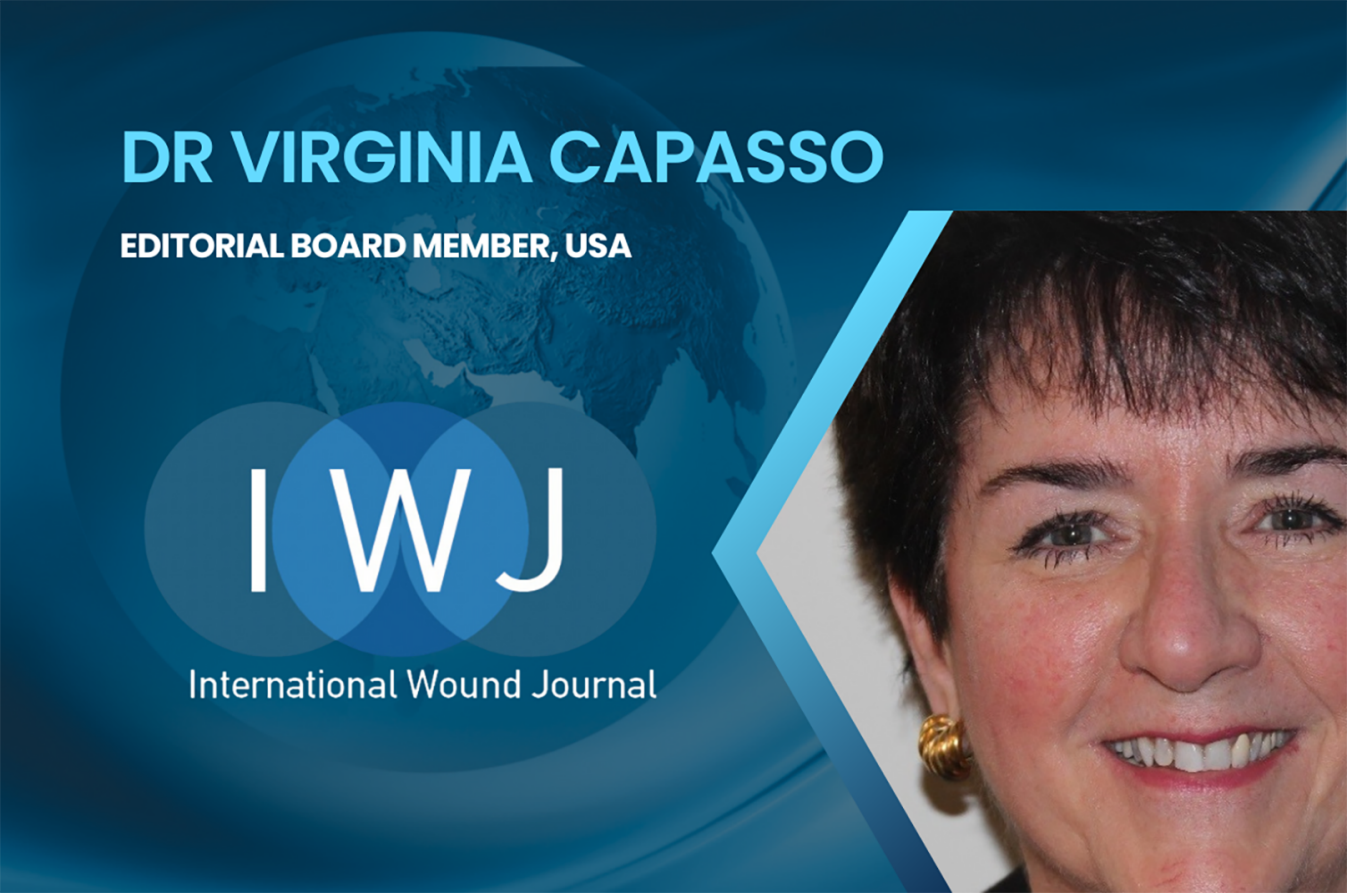

Dr. Virginia Capasso, is an Advanced Practice Nurse for Patient Care Services, Office of Quality, Safety, Informatics and Practice and a Nurse Scientist, Yvonne L. Munn Center for Nursing Research, at Massachusetts General Hospital (MGH) and an Instructor in Surgery at Harvard Medical School. She is a Fellow of the American Academy of Nursing and the American College of Clinical Wound Specialists as well as a Diplomate of the American Board of Wound Management. She was a Director on the Board of Directors of the National Pressure Injury Advisory Panel (NPIAP) from 2018 through 2023. Her other NPIAP roles include Co‐Chair of the Standing Committee on Research (June 2022–December 2023), member of Support Surface Standard Initiative (S3I) [2014‐present], the international Prophylactic Dressing Standards Initiative (PDSI) [2021‐present] and two PDSI subgroups focused on adhesiveness and mechanical properties / durability. Her research has focused on methods of measurement of wound volume and the cost and effectiveness of wound treatments.

**Dr Windy Cole, USA**

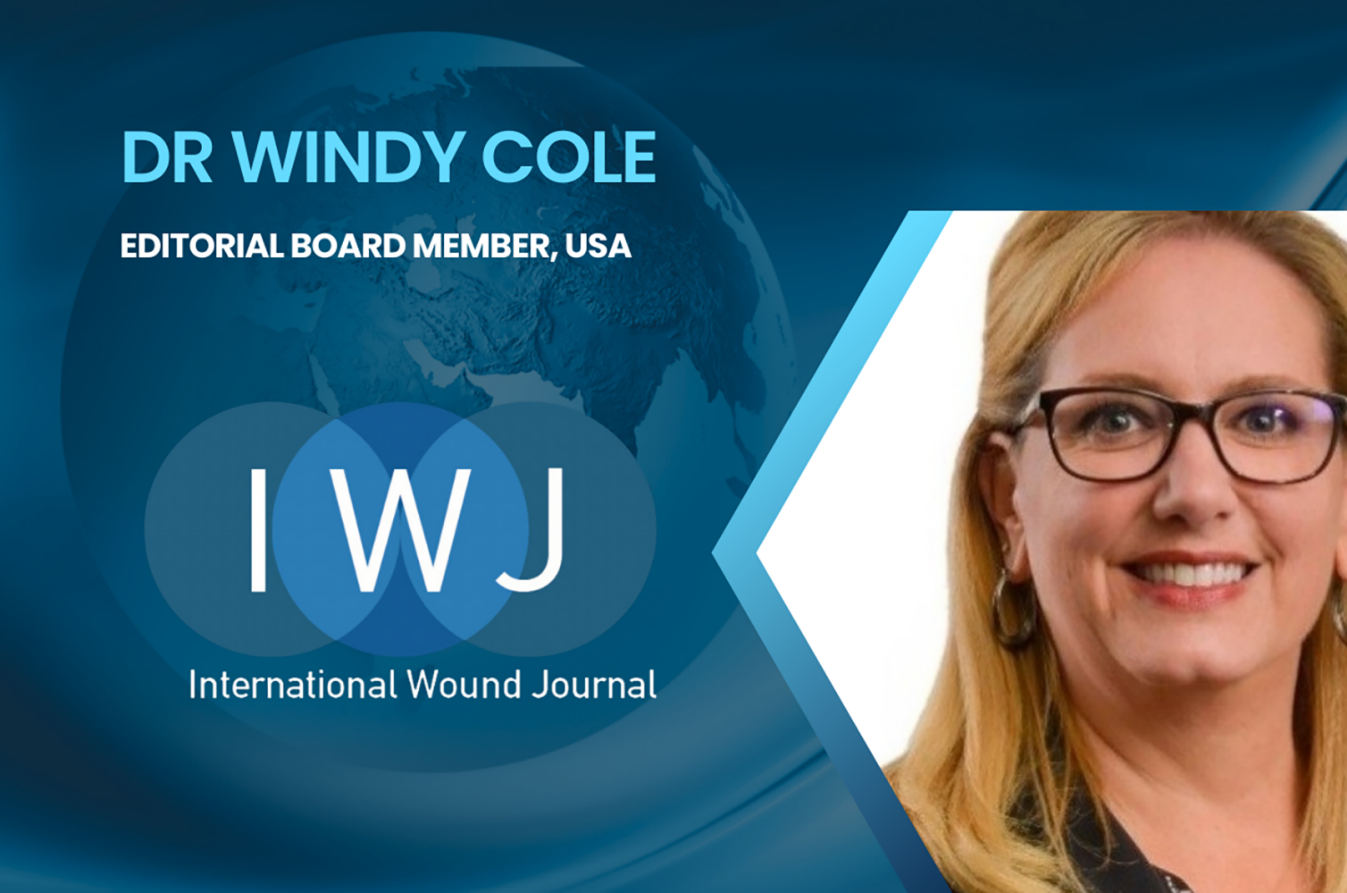

Dr. Windy Cole holds a bachelor's degree in Biology from the University of Cincinnati. She is also an honour graduate from the Kent State University College of Podiatric Medicine. Dr. Cole has practiced in Northeast Ohio for over 22 years. She is an adjunct professor and Director of Wound Care Research at Kent State University College of Podiatric Medicine. She is board‐certified by the American Board of Foot and Ankle Surgery and the American Board of Wound Management. She has been a dedicated wound care advocate for two decades with interests focused on medical education, diabetic foot care, wound care, limb salvage, & clinical research. Dr. Cole has published many peer‐reviewed and industry articles on these topics and is a sought‐after speaker both nationally and internationally. She is an Editorial Board member of Wound Management and Prevention, Podiatry Today, The Foot Journal, Wound Masterclass, Podiatry Management and Lower Extremity Review. She is also the Podiatry Section Editor for the ePlasty Journal and a Guest Editor for Foot and Ankle Quarterly. She is a wound care advocate on the forefront of wound research and was the 2020 World Union of Wound Healing Silver Medal Award recipient for her work in technology‐driven research and the 2022 recipient of the Kaplan‐Kanat Memorial Lecture Award. Dr. Cole is a member of the ACCWS BOD.

**Professor Kara Couch, USA**

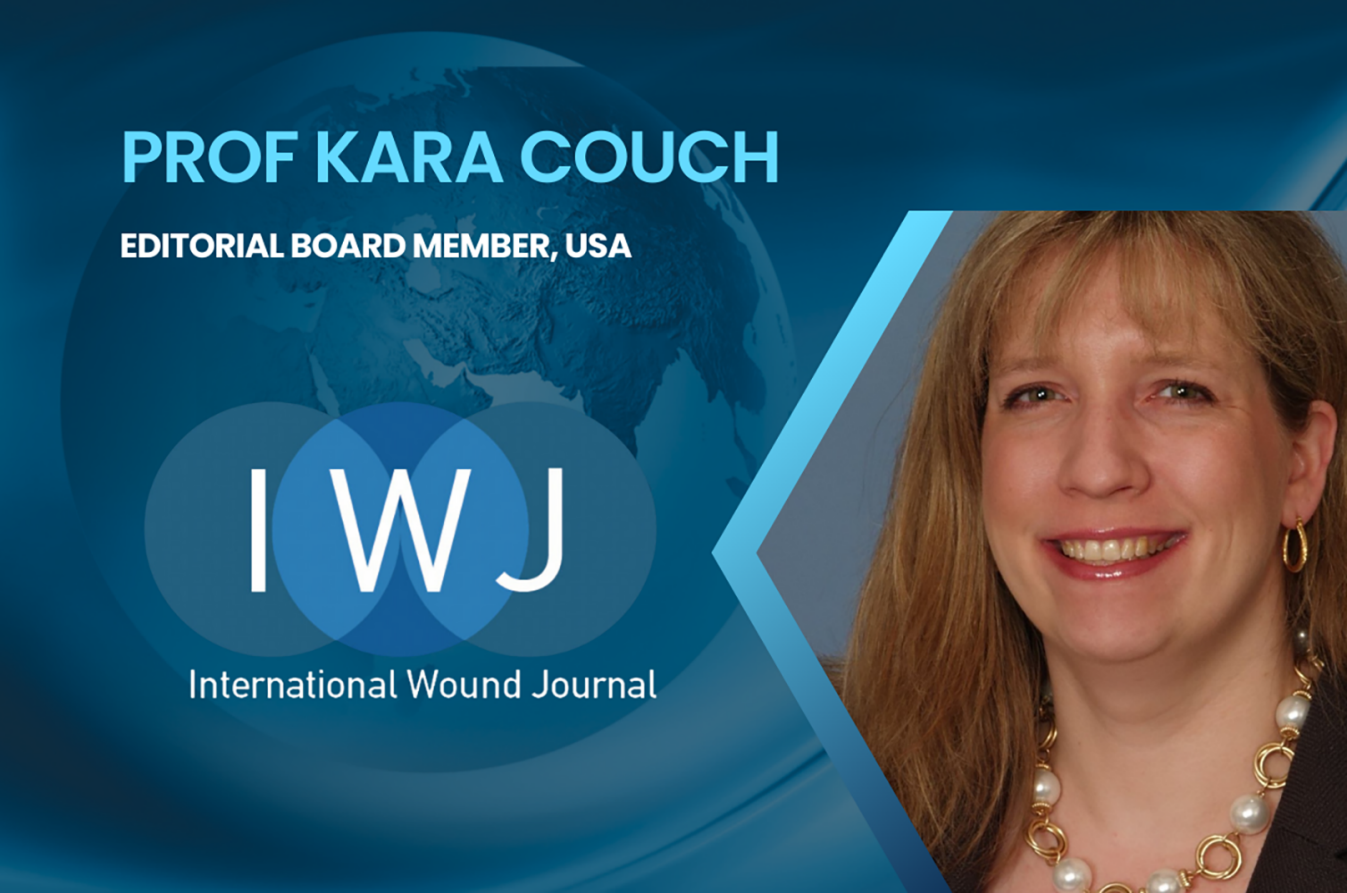

Ms. Couch graduated with her Master of Science in Nursing (FNP) from Georgetown University in 2002. Currently, she works as the Director of Wound Care Services at the George Washington University Hospital in Washington DC. She is an Associate Research Professor of Surgery at the School of Medicine and Health Sciences at George Washington University. Her primary wound interests are in amputee care, wound infection and venous ulcers. Ms. Couch is the current nurse board member of the Alliance of Wound Care Stakeholders and the Secretary of the Association for the Advancement of Wound Care (AAWC). She is an editorial board member of Wound Management and Prevention, Today's Wound Clinic and WoundSource 2021. Kara is the liaison for the American Association of Nurse Practitioners to the Alliance. In 2017, she was voted onto the Scientific and Medical Advisory Committee of the Amputee Coalition. She became Chair of the SciMAC in 2019. She is a member of the Prophylactic Dressing Standards Initiative, a joint collaboration between the NPIAP and EPUAP.

**Professor Janet Cuddigan, USA**

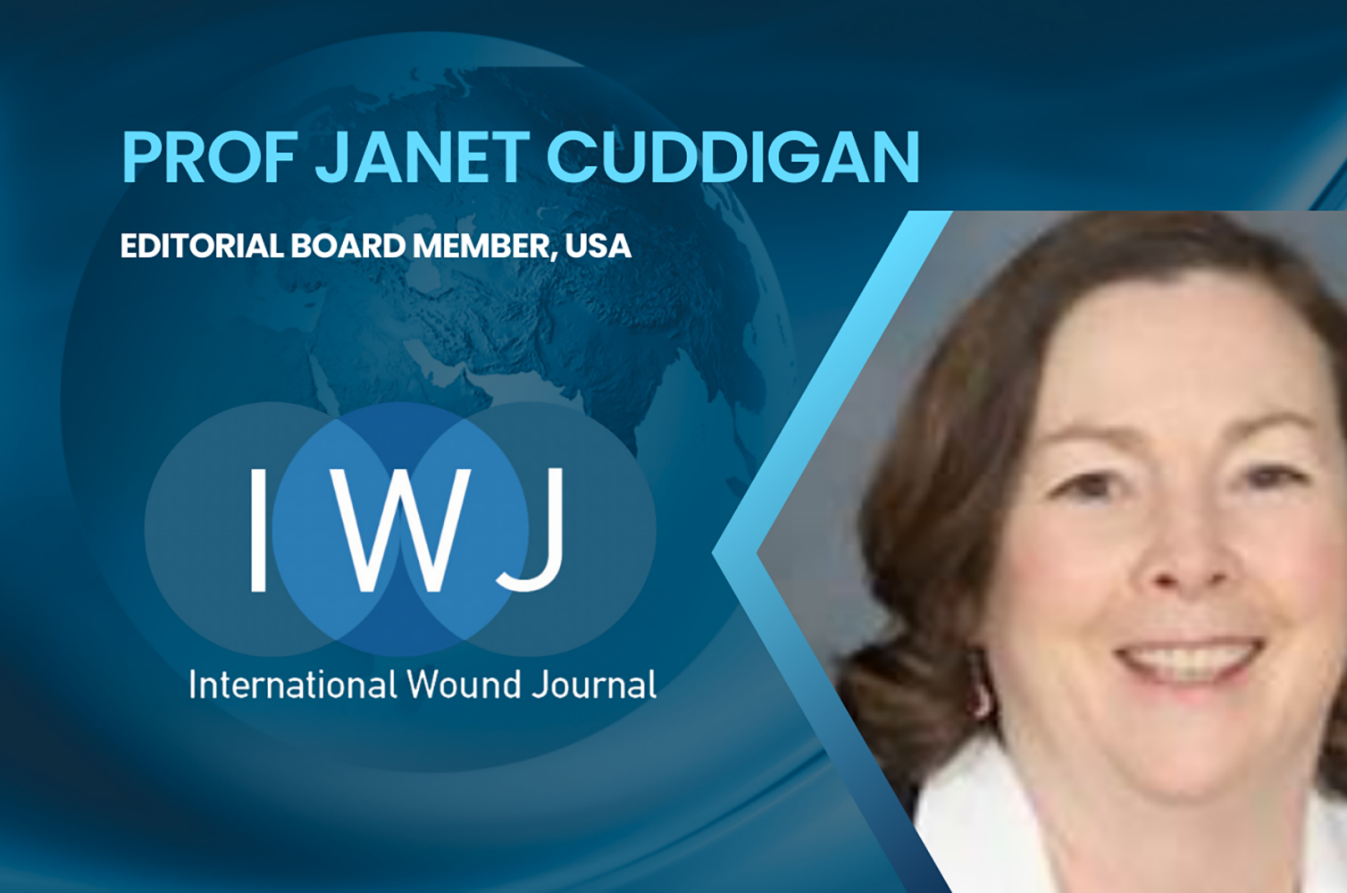

Dr. Cuddigan received her PhD in Nursing from the University of Nebraska Medical Center (1999) and completed a post‐doctoral fellowship at the Veterans Affairs Medical Center in Iowa City and University of Iowa (2003–2005). She has extensive clinical experience in critical care nursing and wound care. Her research interests focus primarily on pressure injury prevention, assessment and treatment, particularly in relation to prevention of device‐related pressure injuries; risk assessment and prevention in critically ill patients; and dissemination & implementation of evidence‐based pressure injury guidelines. She currently teaches interprofessional graduate students and DNP students in research methods, translational research and evidence‐based practice implementation.

Dr. Cuddigan also has extensive experience in the development of evidence‐based guidelines. She was the Panel Manager for the original Agency for Health Care Policy and Research (AHCPR, now AHRQ) guidelines on pressure injuries. She has been a member of three international pressure injury guideline development groups, serving as the Co‐Chair for two international guidelines and the Editor‐in‐Chief for one. She is a nationally and internationally known expert and speaker on pressure injuries. She has served in leadership positions on the National Pressure Injury Advisory Panel (NPIAP) including as the organization's President from 2019 to 2021. She has held several administrative positions at the University of Nebraska Medical Center College of Nursing including as a Department Chair (2006–2014), Interim Associate Dean for Administration (2011) and Acting Dean of the College of Nursing (July–October 2011).

**Professor Stephen Davis, USA**

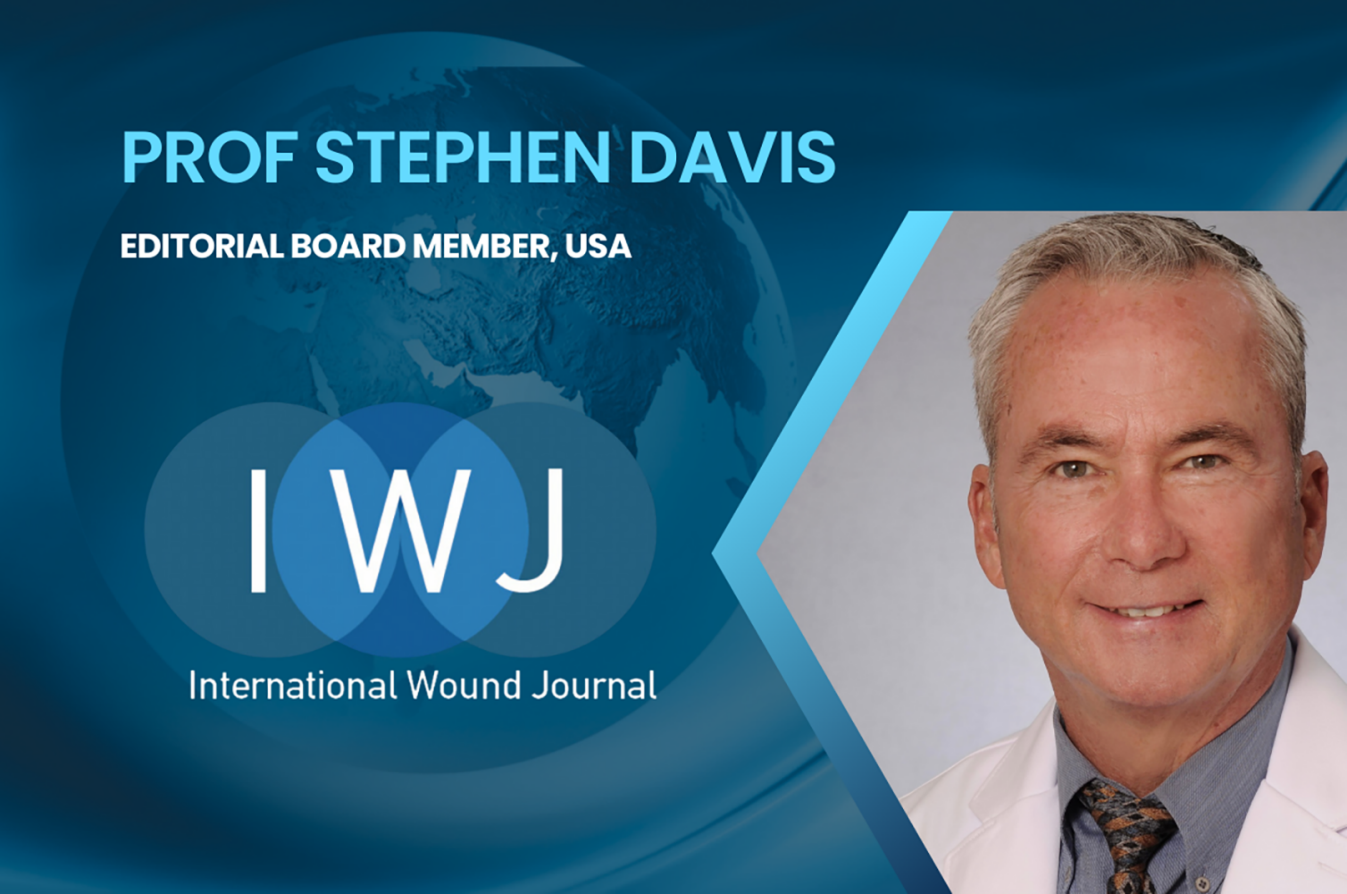

Professor Stephen Davis started his research career in 1984 at the University of Pittsburgh where he worked with Bill Eaglstein, MD and Patricia Mertz who pioneered the porcine models for wound healing and infection. He has been in the Dr. Phillip Frost Department of Dermatology & Cutaneous Surgery at the University of Miami for over 38 years and has been Principal Investigator (PI) or Co‐PI on research grants totaling over $28 million from DARPA, Canadian Defence, US Army, Office of Naval Research, NIH, NSF and various industry. Professor Davis has worked with well over 200 companies in research and development of various products that are on the market today. His research over the years has focused on the role of occlusive therapy, debridement, biofilms, topical oxygen and electric stimulation.

**Professor Anna de Jesus, USA**

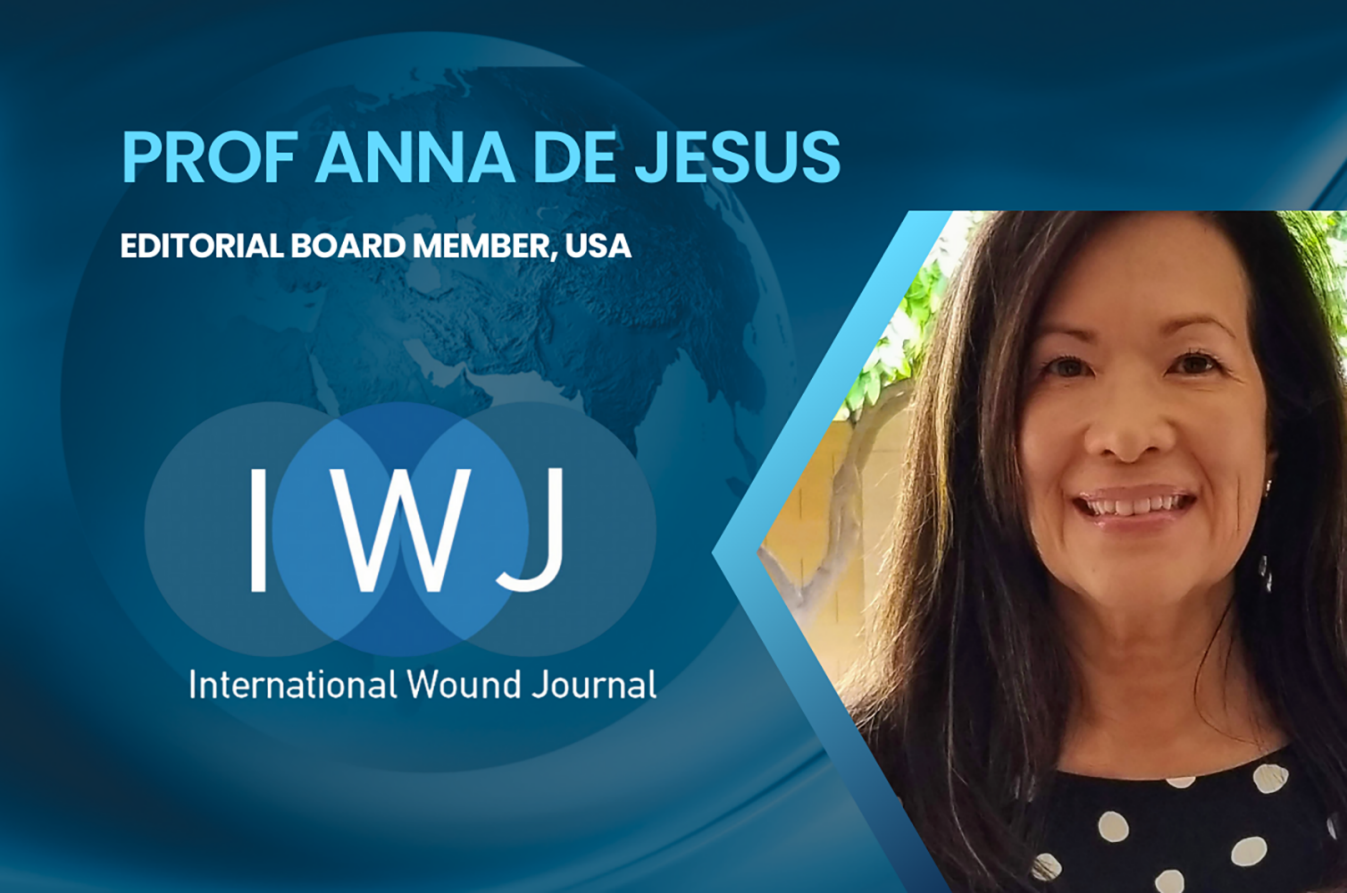

Anna de Jesus is the President and Owner of Food and Nutritional Solutions, LLC providing consultant services to business and industry and public speaking engagements to healthcare clients. She is also the Founder of Nutrition Alliance, LLC, the largest provider of dietitian services for skilled nursing and behavioural hospitals in AZ. Prior to these, Anna was Regional Dietitian for Sunbridge healthcare where she directed the clinical nutrition and food service operations of skilled nursing & assisted living communities in AZ and California. Anna received her Master's degree in Business Administration at Keller Graduate School of Management where she graduated with distinction and her BS in Community Nutrition from the University of the Philippines.

Anna has published several operational and training manuals on clinical nutrition services and food service operations. She developed the Patient‐Driven Payment Model (PDPM) nutrition‐specific process to maximize reimbursement for nutrition‐related triggers for long‐term care communities. She is one of the authors of the newly published, SPIPP 2.0 by the National Pressure Injury Advisory Panel.

Anna is a sought‐after speaker and has presented at various state and national conferences, both onsite and virtually. She has served in various committees for the Dietitians in Health Care Communities, Arizona State University's Internship, Maricopa County Internship, Arizona Health Care Association AZ Chapter's Quality Committee and is currently the Treasurer for the National Pressure Injury Advisory Panel (NPIAP).

**Professor Vickie Driver, USA**

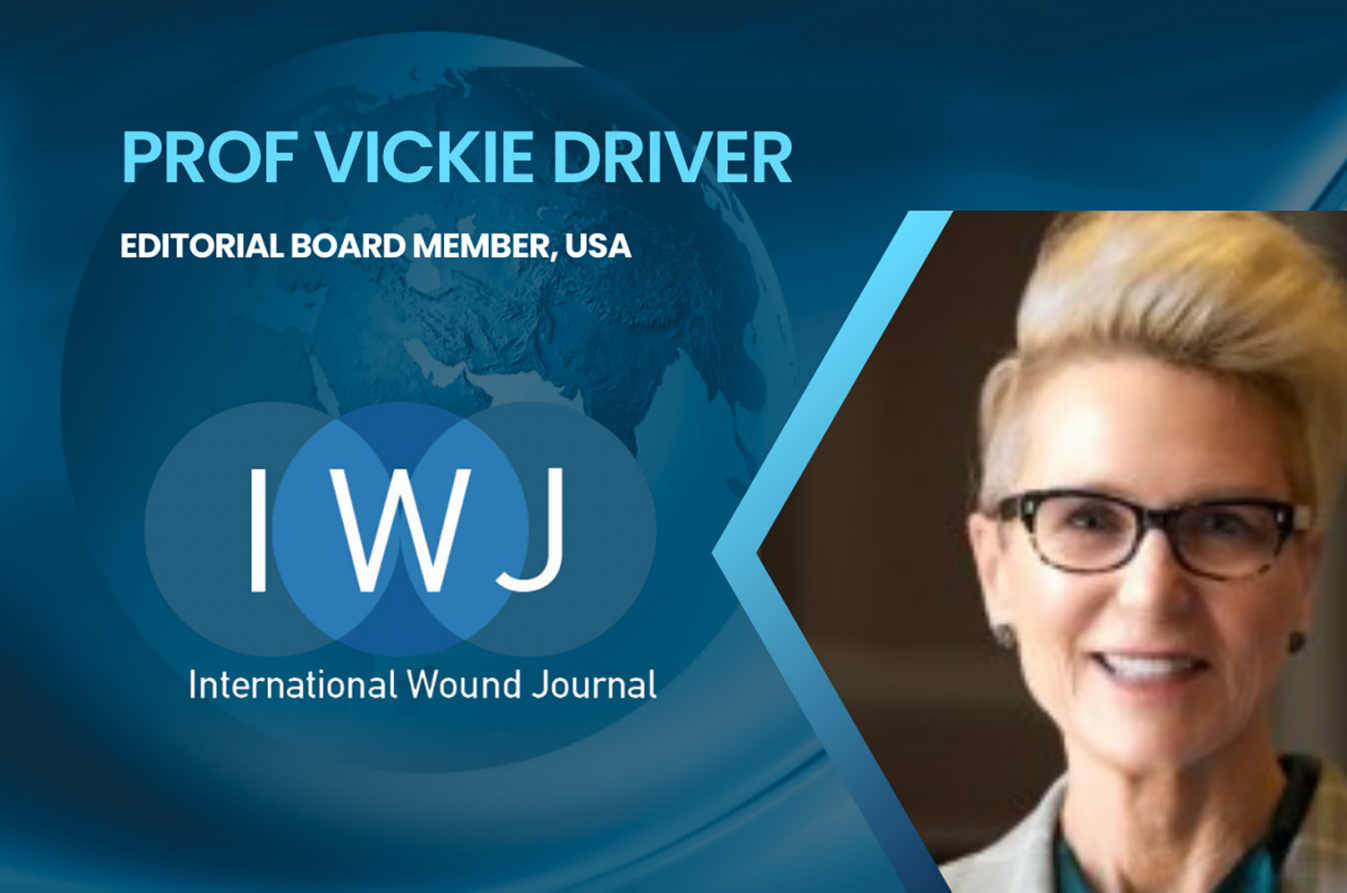

Professor Driver is Professor, Washington State University, School of Medicine and is the Founding Chair, Board of Directors for the Wound Care Collaborative Community, an important collaboration with the FDA, CMS and the NIH. She is also Fellow, Royal College of Physicians and Surgeons‐Glasgow, PM and Inaugural Fellow, Association for the Advancement of Wound Care, FAAWC. Dr Driver received the prestigious honour of receiving The Robert A. Warriner III, MD Memorial Award. She serves as Honorary Visiting Professor at Cardiff University (UK) in the Department of Medicine and Professor‐affiliate at Barry University (USA). Dr Driver serves in multiple key leadership positions across national and international sectors of medicine, academia and the arts.

Her career has a special emphasis on wound healing and limb preservation, and she is proud to be an outspoken ambassador for improving care to patients that face the burden of limb loss.

As lead investigator, she has served on and initiated more than 70 important multi‐centre randomized clinical trials, as well as developed and supervised multiple research fellowship training programmes. She has co‐authored well over 150 publications and abstracts and is former Director, Translational Medicine at Novartis Institute for BioMedical Research. Her love for teaching has transcended her career and continues to be an important focus of her professional attention.

Dr. Driver is very involved in both clinical and translational medical research in the field of wound healing and limb preservation.

She has proudly served as a member of the Wound Healing Society (WHS) Board of Directors and Board of Directors for the Critical Limb Ischemia (CLI) Global Society. She completed her tenure as president for the Advancement of Wound Care Association (AAWC) and served for 9 years on the Board of Directors.

She is Board Certified with American Board of Podiatric Surgeons and is a Fellow at the American College of Foot and Ankle Surgeons, licensed in VA, MA and RI.

**Professor Patricia Dykes, USA**

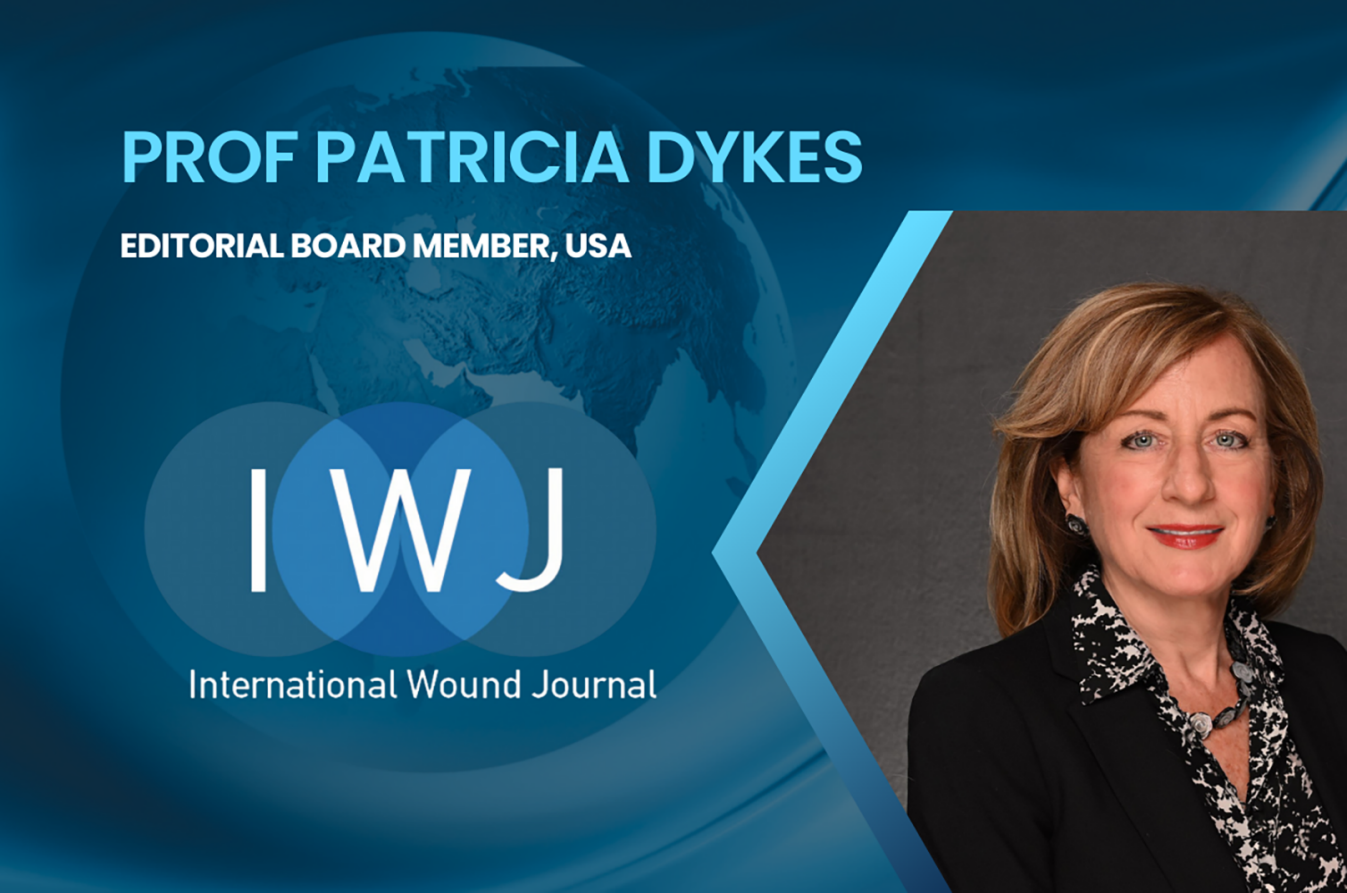

Patricia Dykes is President/Board Chair of AMIA and elected fellow of ACMI. She is an Associate Professor of Medicine at Harvard Medical School and Program Director of Research in the Center for Patient Safety, Research and Practice at Brigham and Womenís Hospital. Her research aims to improve quality and safety through patient engagement and CDS. To reduce falls and injuries, she developed the Fall TIPS Toolkit (www.FallTIPS.org) which has been shown to reduce falls and injuries and is adopted in over 250 hospitals in the United States including the VA and DOD. Currently, she is leading two federally funded projects to improve fall prevention CDS in primary care including developing a care plan collaboration tool and personalized exercise prescriptions. Dr. Dykes is also leading the development of a set of eCQMs for CMS and for the Gordon and Betty Moore Foundation. She is the author of two books and over 150 peer‐reviewed publications.

**Professor Laura Edsberg, USA**

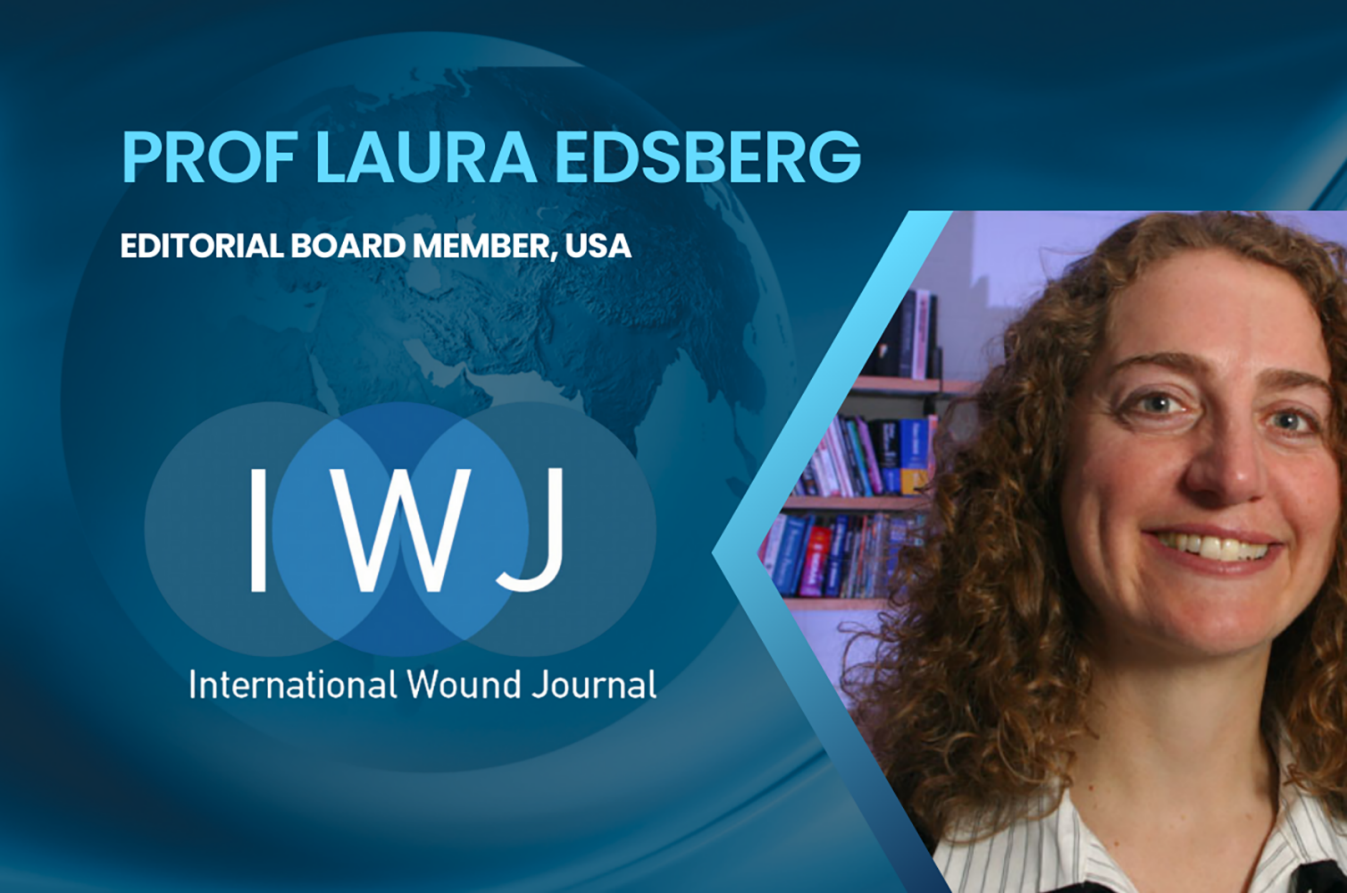

Dr. Laura Edsberg is Professor of Natural Sciences and Director of the Center for Wound Healing Research at Daemen University in Amherst, NY. She is also Co‐Founder and Co‐Director of the Daemen University Institute for Mobility Innovation & Technology (IMIT). Dr. Edsberg is a past‐President of the National Pressure Injury Advisory Panel (NPIAP). She is currently serving on the NPIAP board of directors. She co‐chaired the 2016 NPIAP Staging Task Force, as well as the NPIAP Unavoidable Pressure Injury Consensus Conferences. She has written numerous articles and chapters about the microstructural and mechanical properties of pressure injury tissue and the proteomics of wound healing. Dr. Edsberg was awarded the Kosiak Award for her work in the field of pressure injuries. Dr. Edsberg's research is focused on the proteome of healing and non‐healing wounds. Currently, her research group is working to identify biomarkers associated with healing in wounds.

**Professor Mohamed El Masry, USA**

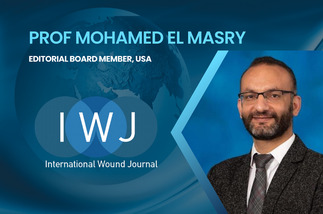

Dr. Mohamed Salah El Masry has received his medical degree from Zagazig University, School of Medicine (Egypt) where he also completed his general surgery residency and plastic surgery training. He earned his doctoral degree in Plastic Surgery from Zagazig University, School of Medicine and The Ohio State University via a jointly sponsored programme.

Prior to joining the McGowan Institute and the University of Pittsburgh, Dr. El Masry was an Assistant Professor, Division of Plastic Surgery, Department of Surgery at Indiana University, as well as an investigator at the Indiana Center for Regenerative Medicine and Engineering (ICRME).

Dr. El Masry's primary research focus is regenerative medicine in wound healing. He developed a clinically relevant swine model that reproducibly recapitulates features characteristic of human critical limb ischaemia (CLI) that is validated comprehensively by both non‐invasive and invasive assessment and that lends itself to detailed mechanistic studies and interventional drug testing.

Additionally, Dr. El Masry has studied the effect of tissue scaffolds on the wound microenvironment by utilizing a stabilized collagen matrix dressing. Dr. El Masry's laboratory is currently working on a novel wound care dressing to combat multidrug‐resistant biofilm.

Dr. El Masry has published in peer‐reviewed journals such as Molecular Therapy and The Federation of American Societies for Experimental Biology Journal (FASEB J). He serves as a co‐investigator on several grants funded by the National Institutes of Health (NIH) and the U.S. Department of Defence (DoD). He serves on the editorial board of Advances in Wound Care and is on the education committee of the Wound Healing Society.

**Elizabeth Faust, USA**

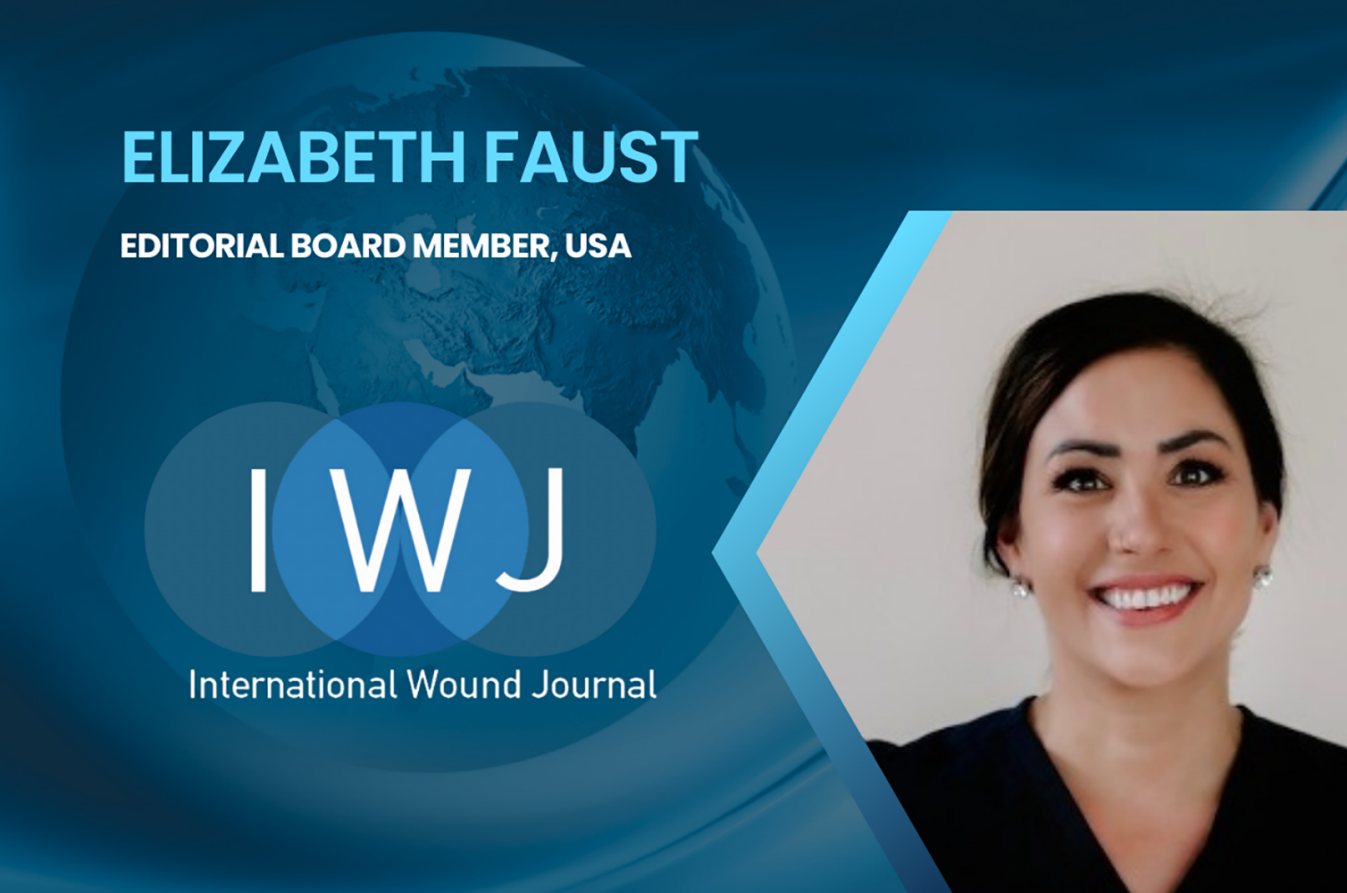

Elizabeth Faust, known as Liz or Lizzie, is a Nurse Practitioner and Wound, Ostomy and Continence specialist. She graduated from Gwynedd Mercy University with her MSN in Adult Nurse Practitioner in 2009 and went on to become a CWS, CWOCN, CWOCN‐AP and CSWS. She served for 13 years at Tower Health System in West Reading, PA in a variety of roles including inpatient CWOCN, system administrative lead for Wound Ostomy practices and department head of inpatient wound care under plastic surgery at a 700+ Level 1 Trauma Center in Eastern Pennsylvania. She previously served as a Nurse practitioner in an outpatient wound care centre with a physical therapy model. She has a passion for education, particularly with NPWT and Pressure injury prevention and management. She has lectured internationally, in addition to facilitating Bioskills laboratories across the country. She presently runs a consulting business, Lizzie Wounds, LLC, that focuses on education, product development, legal case reviews and research. She also serves as a co‐host for an educational channel for wound care clinicians called The Frank & Lizzie Show.

**Professor Robert Frykberg, USA**

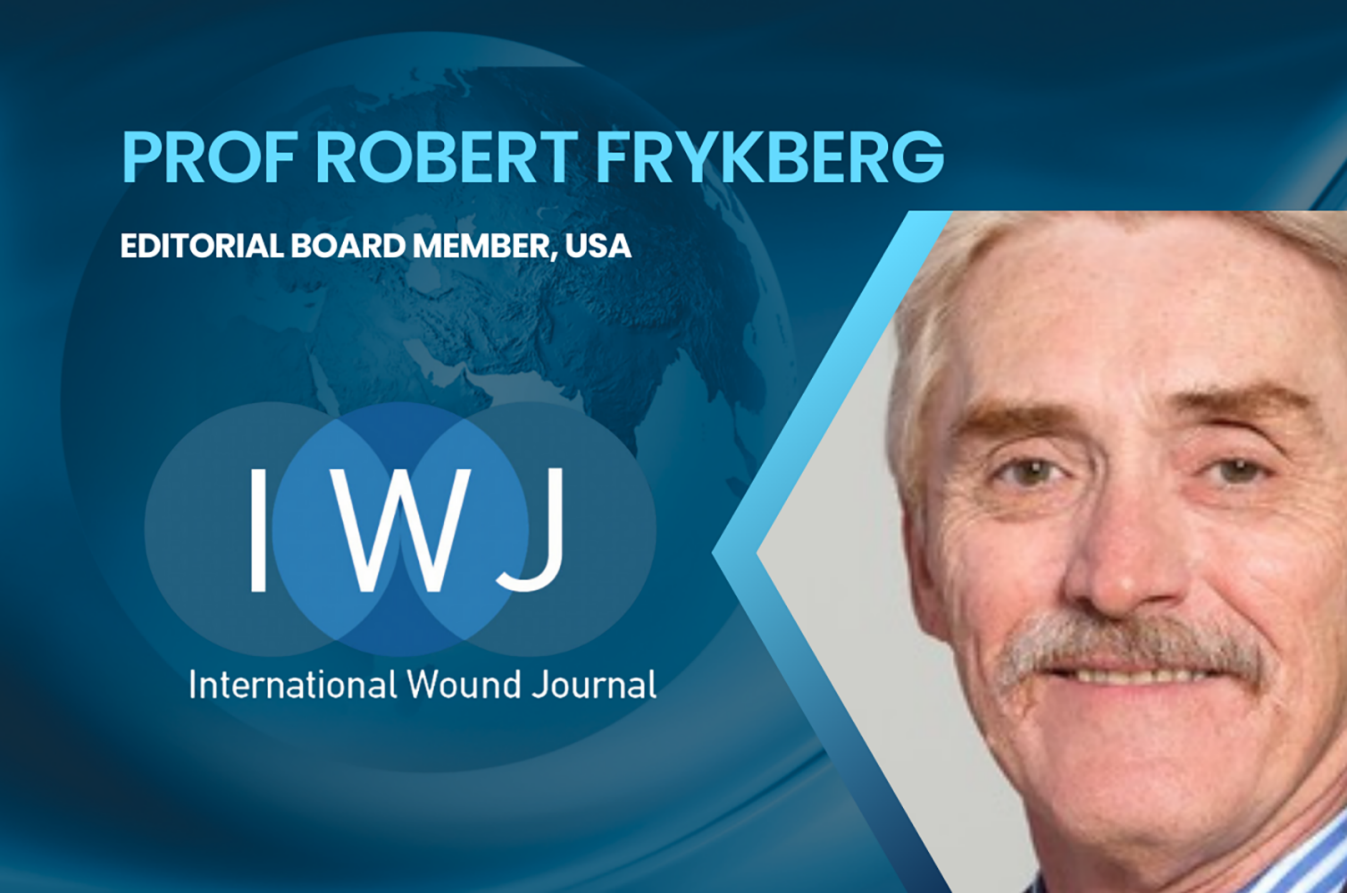

Professor Frykberg is the Senior Medical Advisor to McCord Research and Chief Research Officer of Open Wound Research. Dr. Frykberg was the former Chief of the Podiatry section at the Phoenix Veterans Affairs Medical Center in Phoenix, Arizona. He holds Faculty ranks as Adjunct Professor, Midwestern University Program in Podiatric Medicine as well as Honorary Professor of Podiatric Medicine at the University of Galway.

A former Chair of the Foot Care Council of the American Diabetes Association and Past President of the American College of Foot and Ankle Surgeons, Dr. Frykberg was the 2011 recipient of the prestigious Roger Pecoraro Award from the Foot Care Council of the American Diabetes Association.

He received his Master of Public Health degree from the Harvard School of Public Health in 1994 with a concentration in quantitative methods.

Dr. Frykberg's research and writing interests have included all facets of lower extremity disorders, but specifically focused on diabetic foot ulcers, infections, Charcot foot, Surgery and Wound care. The author of over 100 peer‐reviewed publications and textbook chapters, he has also been the Editor of two textbooks. Prof. Frykberg is currently a Director of the Association of Diabetic Foot Surgeons, a Fellow of the Faculty of Podiatric Medicine of the Royal College of Physicians and Surgeons of Glasgow and a Fellow, Royal Society of Medicine.

**Professor Susan Garber, USA**

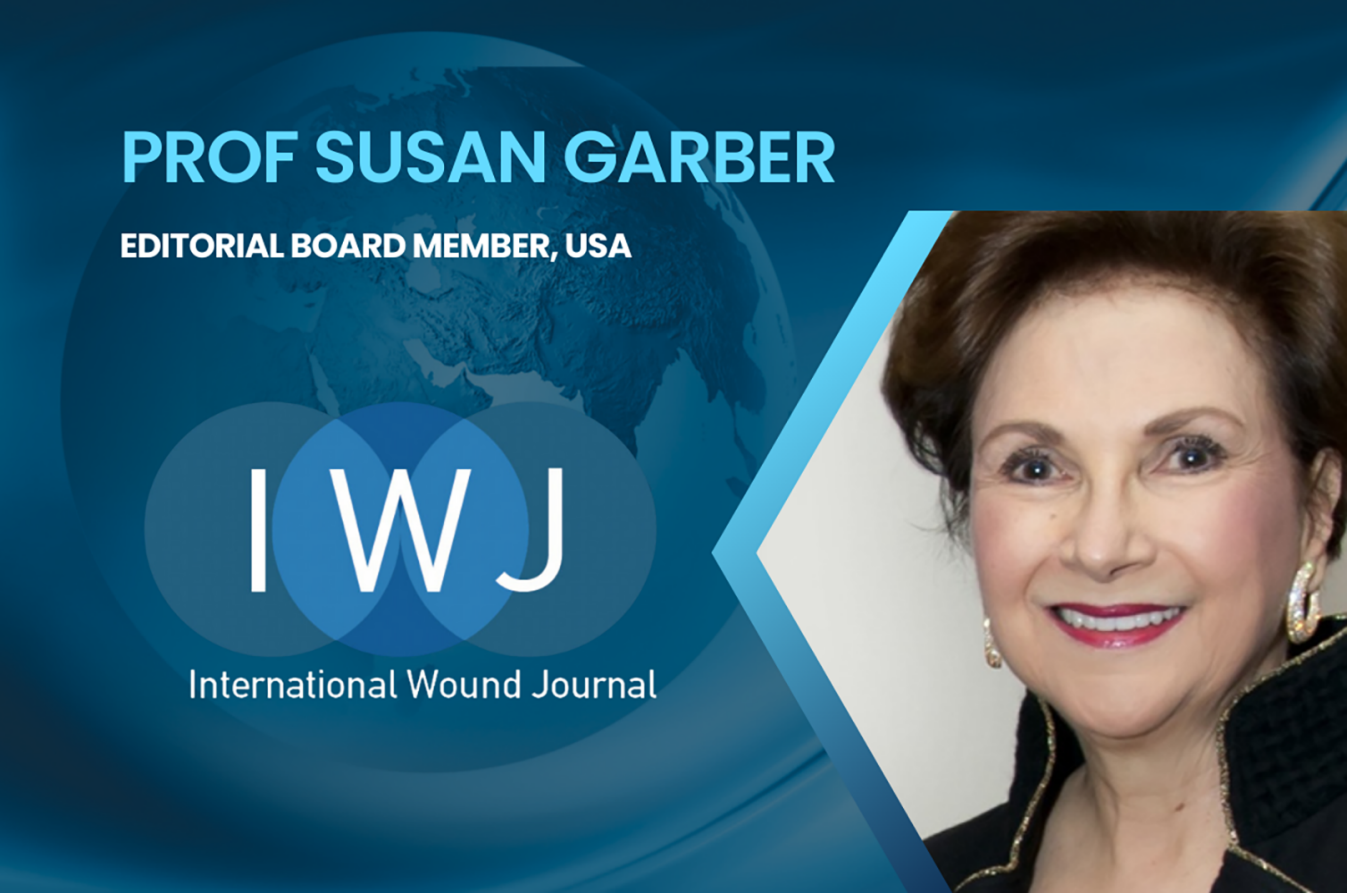

Ms. Garber is a Professor in the Department of Physical and Rehabilitation Medicine at Baylor College of Medicine in Houston, Texas. She has dedicated her career to researching prevention and treatment of pressure ulcers in people with spinal cord injuries and has made significant contributions to this field. She joined the faculty at Baylor after working as a full‐time clinical occupational therapist. She spent 19 years conducting research at The Institute of Rehabilitation and Research (TIRR) as assistant director for research and education in the department of occupational therapy and then 10 years conducting research through the Michael E. DeBakey Veterans Affairs Medical Center. Her research interests are in the areas of spinal cord injury, prevention and treatment of pressure ulcers, rehabilitation outcomes, technology and rehabilitation and patient and family education.

She received her Bachelor of Science in occupational therapy from Columbia University and her Master of Arts in occupational therapy at Texas Women's University. Ms. Garber is a recipient of the AOTA Eleanor Clarke Slagle Lectureship Award, and is a Fellow of the American Occupational Therapy Association as well as the American Congress of Rehabilitation Medicine. Ms. Garber was named one of the 100 Influential People of Occupational Therapy by AOTA.

Ms. Garber is a member of the AOTF Board of Trustees and was named one of the 100 Influential People of Occupational Therapy.

**Professor Allen Holloway, USA**

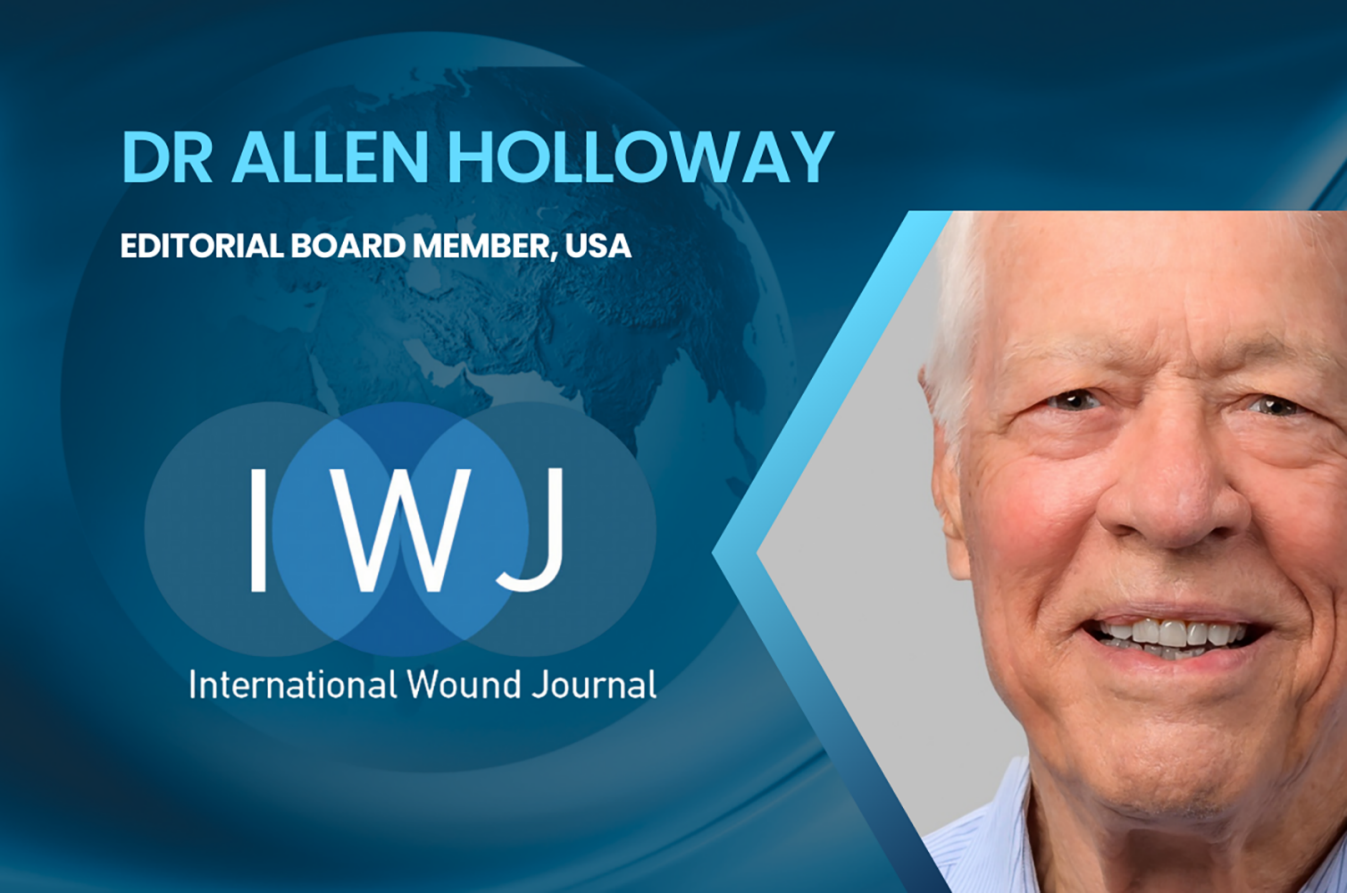

Dr. Holloway is a specialist in wound care. He graduated from Harvard Medical School, and received post‐graduate training in pathology, medicine and nephrology at Harvard, UCLA and the University of Washington where he also served on the faculty of the Center for Bioengineering where he was the recipient of a Research Career Development Award by the National Institutes of Health. Recently, he has served in the Department of Surgery at Maricopa Medical Center (now Valleywise Health), and was Director of the Burn Clinic in his last 12 years there. He has been published in many medical journals and serves on the Editorial Board of ‘Wounds’. He lectures nationally and internationally and has served as President of the Wound Healing Society. His current practice is in providing wound care, in which he has had clinical and research interests for many years, to institutionalized and home‐bound patients.

**Professor Chantal Labrecque, Canada**

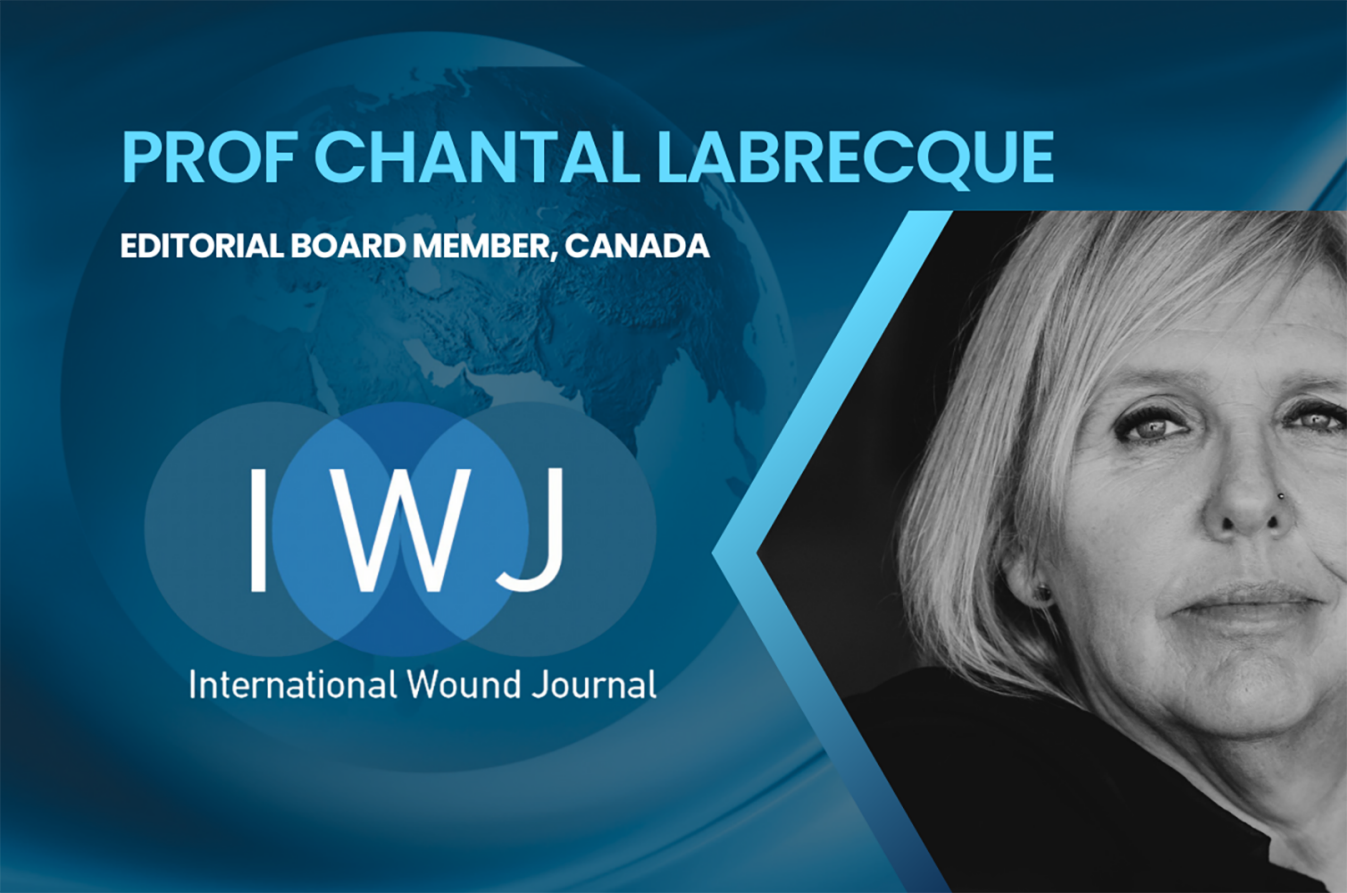

Chantal Labrecque has been a nurse for over 30 years and has specialized in the area of wound healing and wound care for over 25 years. She holds a master's degree and a PhD in nursing sciences. Her doctoral studies focused on body image and wounds. She was a lecturer for 26 years in several universities and she is currently a regular professor in the Department of Nursing at the Université du Québec en Outaouais (UQO). She is actively involved in the field of wound care at provincial (Quebec) and national (Canada) levels and sits on various advisory committees as well as expert committees. She also founded the Regroupement Québécois en Soins de Plaies (RQSP) in 2012. She owns her own consulting and clinical project management firm in wound care – CliniConseil Inc. She is also an author, researcher and has numerous publications, conferences and courses on the theme of wound care.

**Professor John Lantis, USA**

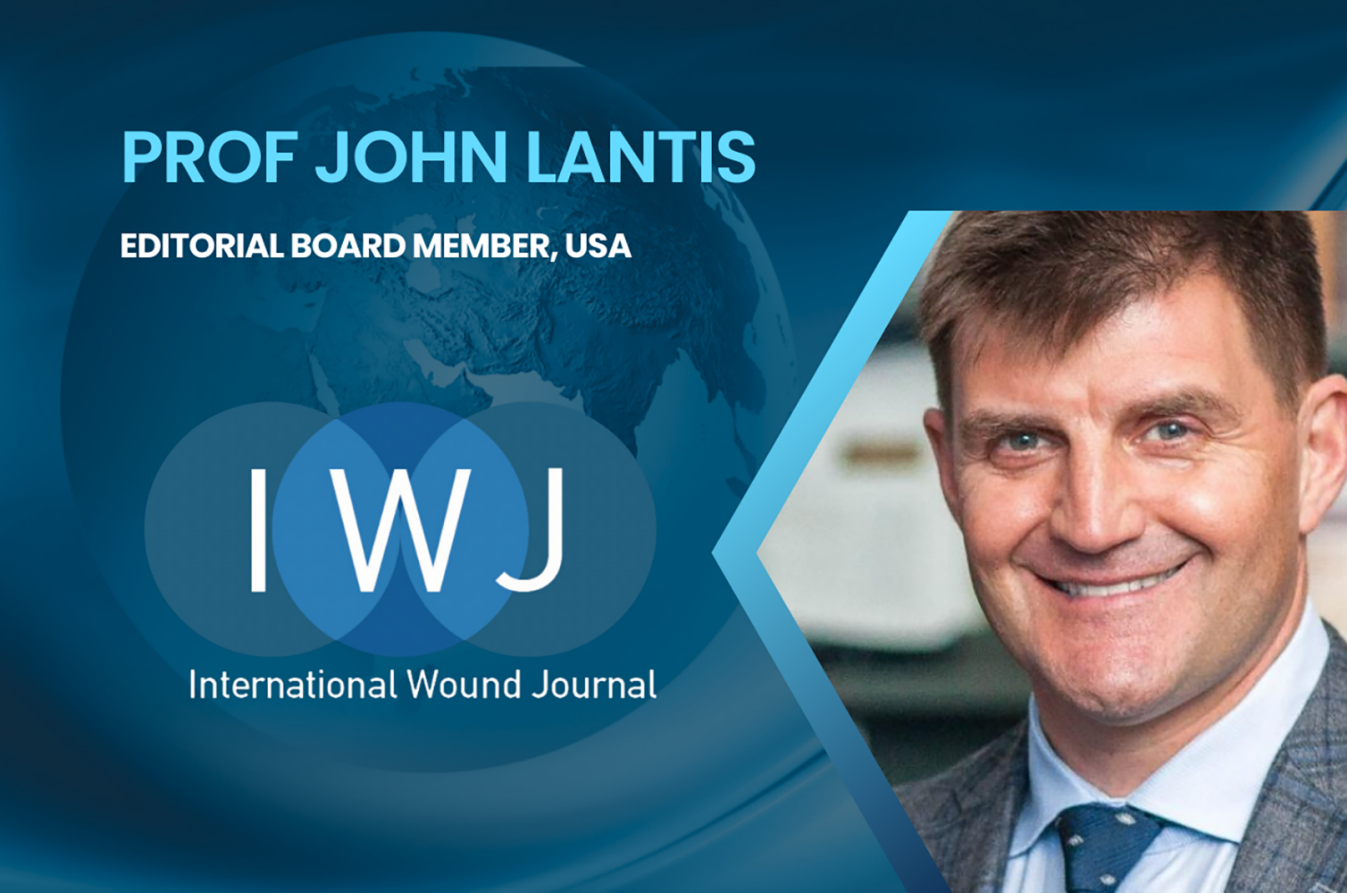

Dr. John Lantis is the Site‐Chief of Surgery at the Mount Sinai West Hospital and Professor of Surgery at the Icahn School of Medicine. Previous to October 2020, he served as the Vice Chairman and Professor of Surgery at Mount Sinai West and Morningside Hospitals from 2013; and the Chief of Vascular and Endovascular Surgery since 2007. He is the past president of the New York Vascular Society and a founding member of the Vascular Study Group of Greater New York and the American Board of Wound Medicine and Surgery.

He is on the editorial boards of numerous peer‐reviewed journals and to date, he is/or has been the principal investigator on more than 70 multi‐centre and single‐centre chronic wound and vascular surgery trials.

Dr. Lantis is an active vascular surgeon treating all facets of vascular disease with the full array of open and endovascular techniques. He serves and has served on the steering committee of many national and international wound healing and limb salvage trials. He currently directs an active Wound and Vascular Surgery research fellowship.

**Professor Lawrence Lavery, USA**

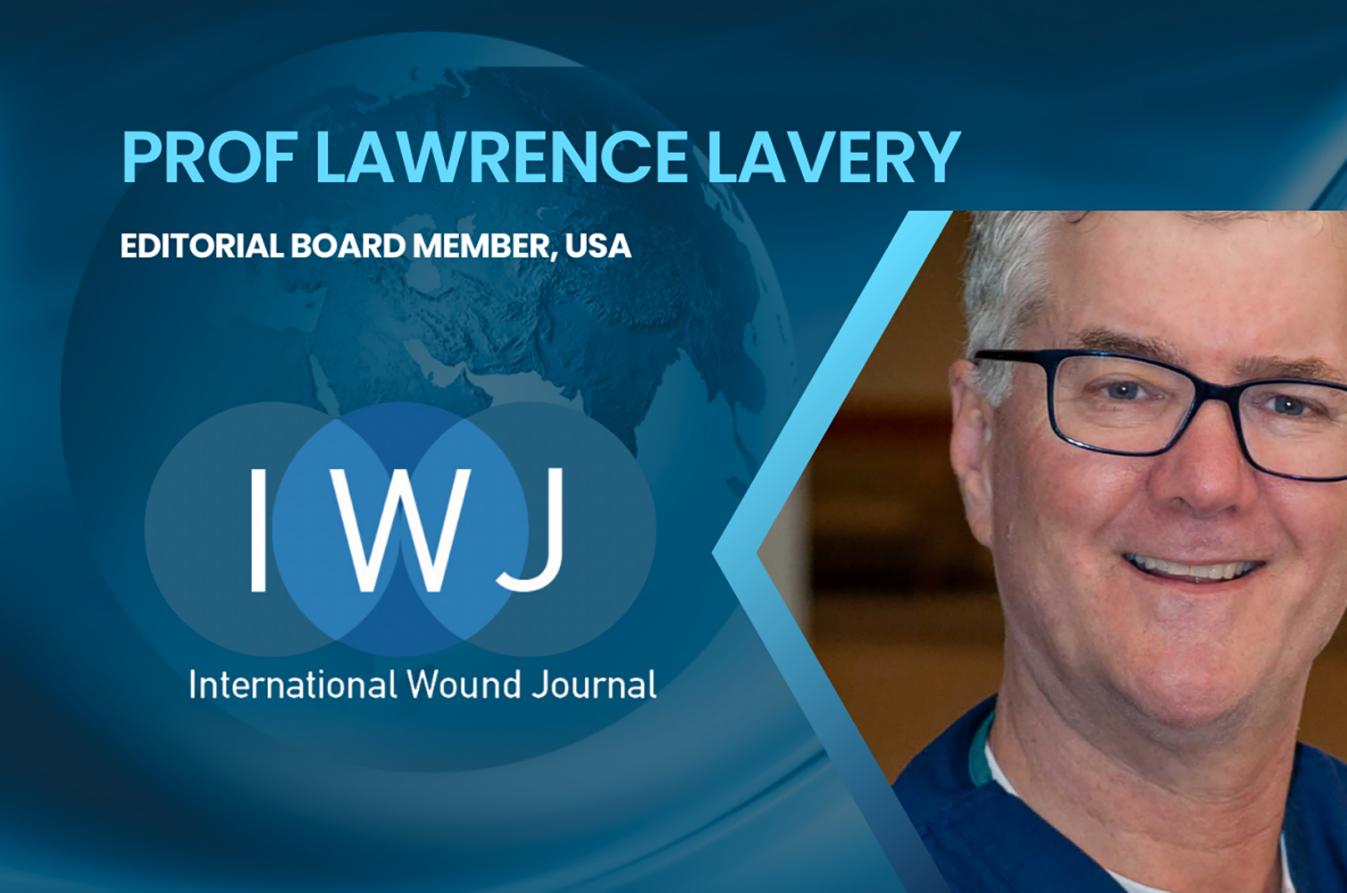

Lawrence A. Lavery, is a Professor in the Department of Plastic Surgery at UT Southwestern Medical Center. He is also the Medical Director of the Diabetic Limb Salvage (DLS) programme at Parkland Memorial Hospital and works as part of the DLS team at William P. Clements Jr. University Hospital. Dr. Lavery's clinic and research interests involve diabetic foot complications, infections and wound healing.

Dr. Lavery completed his undergraduate studies at Indiana University and then earned his medical degree at the Rosalind Franklin University of Medicine and Science, Dr.

William Scholl College of Podiatric Medicine in Chicago. He completed a residency in podiatric medicine and surgery at the University of Texas Health Science Center in San Antonio, where he also earned a Master's in Public Health.

He is board‐certified by the American Board of Podiatric Surgery and a Fellow of the American College of Foot and Ankle Surgeons and the Royal College of Surgeons (Glasgow).

Dr. Lavery's research group has published over 320 peer‐reviewed scientific papers and textbook chapters. His H‐index is 91. They have received research funding from the National Institutes of Health, Agency for Health Care Policy and Research, American Diabetes Association, Veterans Administration, Qatar National Research Foundation, American College of Foot and Ankle Surgeons, American Podiatric Medical Association and private industry.

**Dr Kimberly LeBlanc, Canada**

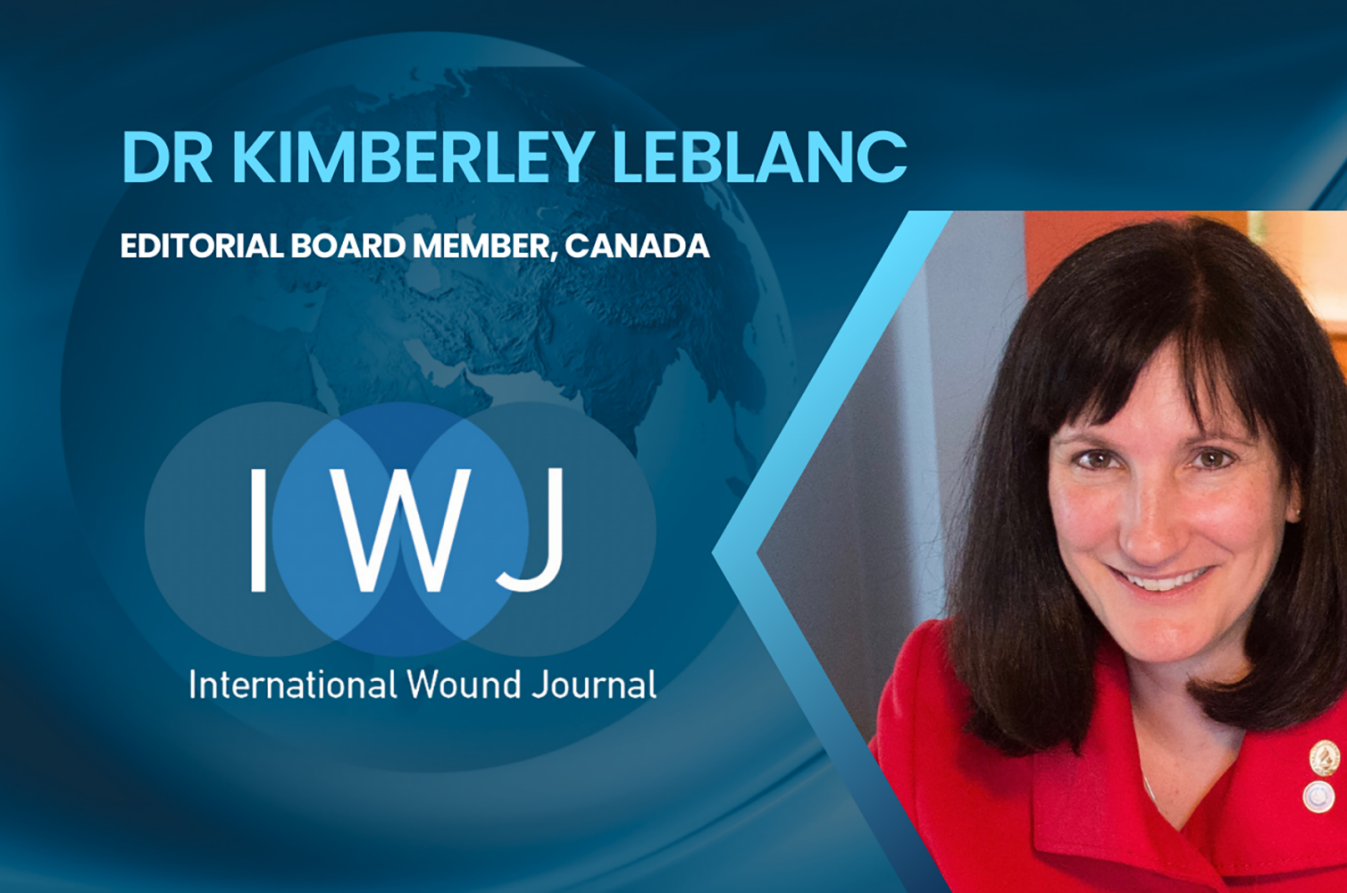

Kimberly LeBlanc is known globally as an innovative nurse leader. She is the current Academic Chair of the Association for Nurses Specialized in Wound Ostomy and Continence Canada's (NSWOCC) Wound Ostomy and Continence Institute (WOC Institute), and an Advanced Practice and Certified Wound, Ostomy and Continence nurse working with KDS Professional Consulting. Kimberly is the President (2024–2026) of the Canadian Nurses Association. She holds a Doctorate of Philosophy in Nursing from Queen's University. In 2020, she was inducted as a Fellow of the Canadian Academy of Nursing (FCAN) and is a past recipient of the Canadian Nurses Association Order of Merit for Nursing Education. Kimberly is an Adjunct Professor at Curtin University, an Affiliate Lecturer at McGill University and an Honorary Senior Lecturer at Cardiff University.

Kimberly is a past president and a founding member of the International Skin Tear Advisory Panel and the past co‐chair of the Canadian Pressure Injury Advisory Panel. She is currently a board member of the International Wound Infection Institute (IWII) and the Commonwealth Wound Care Resource Alliance (CWCRA).

Kimberly has lectured extensively on wound and ostomy care and is considered a global expert on wounds and ostomy issues in the aging population and has numerous publications and book chapters on wound, ostomy and continence‐related topics. She sits on the editorial boards of the International Wound Journal, Advances in Skin & Wound Care and Canadian Nurse. Kimberly maintains an active clinical practice addressing the wound, ostomy and continence needs of individuals across the spectrum of care.

**Professor Jeffrey Levine, USA**

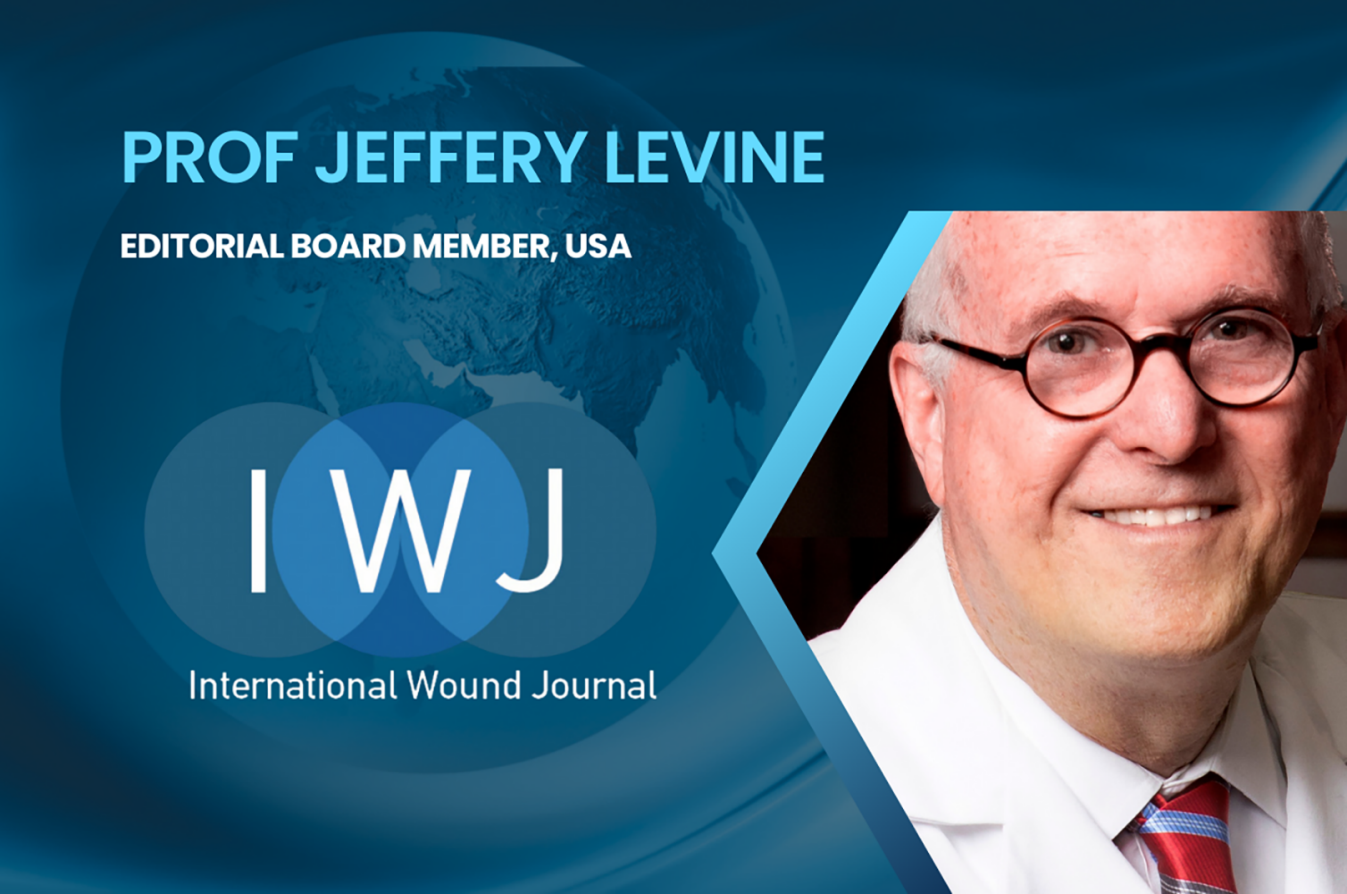

Prof. Levine is a Clinical Professor of Geriatric Medicine and Palliative care at the Icahn School of Medicine at Mount Sinai. Dr. Levine's experience in wound care includes the entire continuum of outpatient, hospital and post‐acute long‐term care. Dr. Levine is an alumnus of the National Pressure Injury Advisory Panel (NPIAP). His many publications include the wound care chapter in the Geriatric Review Syllabus published by the American Geriatrics Society and explorations into the fascinating history of wound care. He is also an accomplished artist and photographer whose images have appeared in numerous publications including The Gerontologist, the Journal of the American Medical Directors Association and the Journal of the American Geriatrics Society.

**Professor Brock Liden, USA**

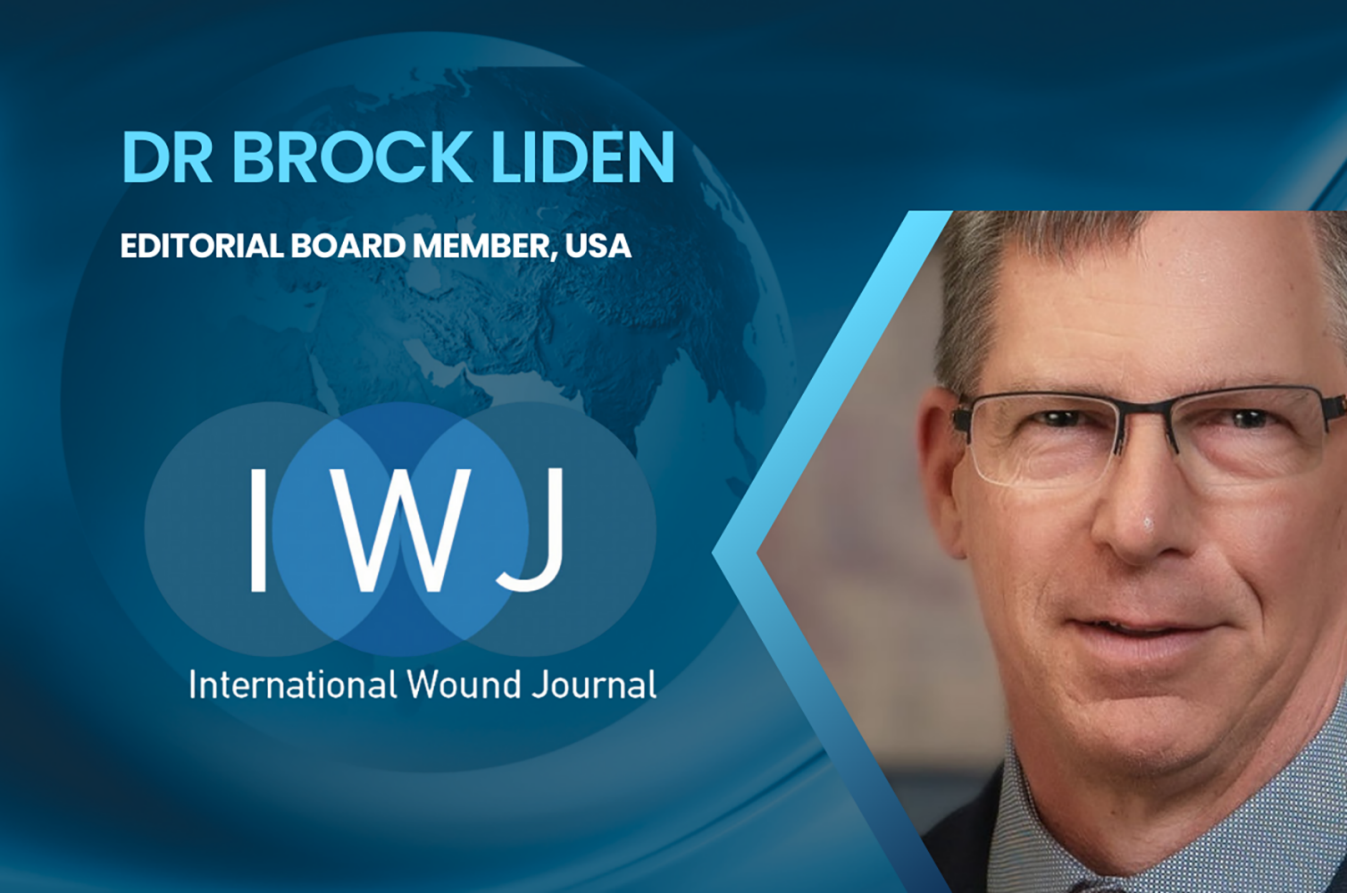

Dr. Liden received his degree from the Ohio College of Podiatric Medicine and served his residency at the Veterans Medical Center in Cleveland, Ohio. He is board‐certified in Podiatric Medicine, Wound Care, Diabetic Wounds and Diabetic Footwear.

Dr. Liden has worked in the Columbus, Ohio‐area for over 24 years. He sees patients in the OhioHealth Berger Hospital Wound Clinic, Hocking Valley Hospital Wound Clinic, as well as in his primary office located in Circleville, Ohio. He established and served as Co‐Medical Director of Berger Hospital's Wound Healing Center with Hyperbaric Oxygen Therapy for 4 years. His areas of specialty include Limb Salvage; Charcot Reconstruction; Diabetic Ulcer Care; Wound Care; Achilles Tendon Repair; Sports Medicine; Dermal Substitutes and Collagen Products.

Dr. Liden is actively engaged in clinical research, product research and product development. He is an international speaker on wound healing and skin substitutes for the advancement of wound healing. He has held faculty positions at multiple wound conferences and has multiple publications and posters. His research has focused on collagen products and limb salvage. He has played a critical role in numerous wound care products currently available. He designed and sold an off‐loading system for DFUs and evolved an autogenic stem cell system to support healing in limb salvage. Dr. Liden is an accomplished researcher and scientist who has dedicated his life to wound healing.

**Professor Benjamin Lipsky, USA**

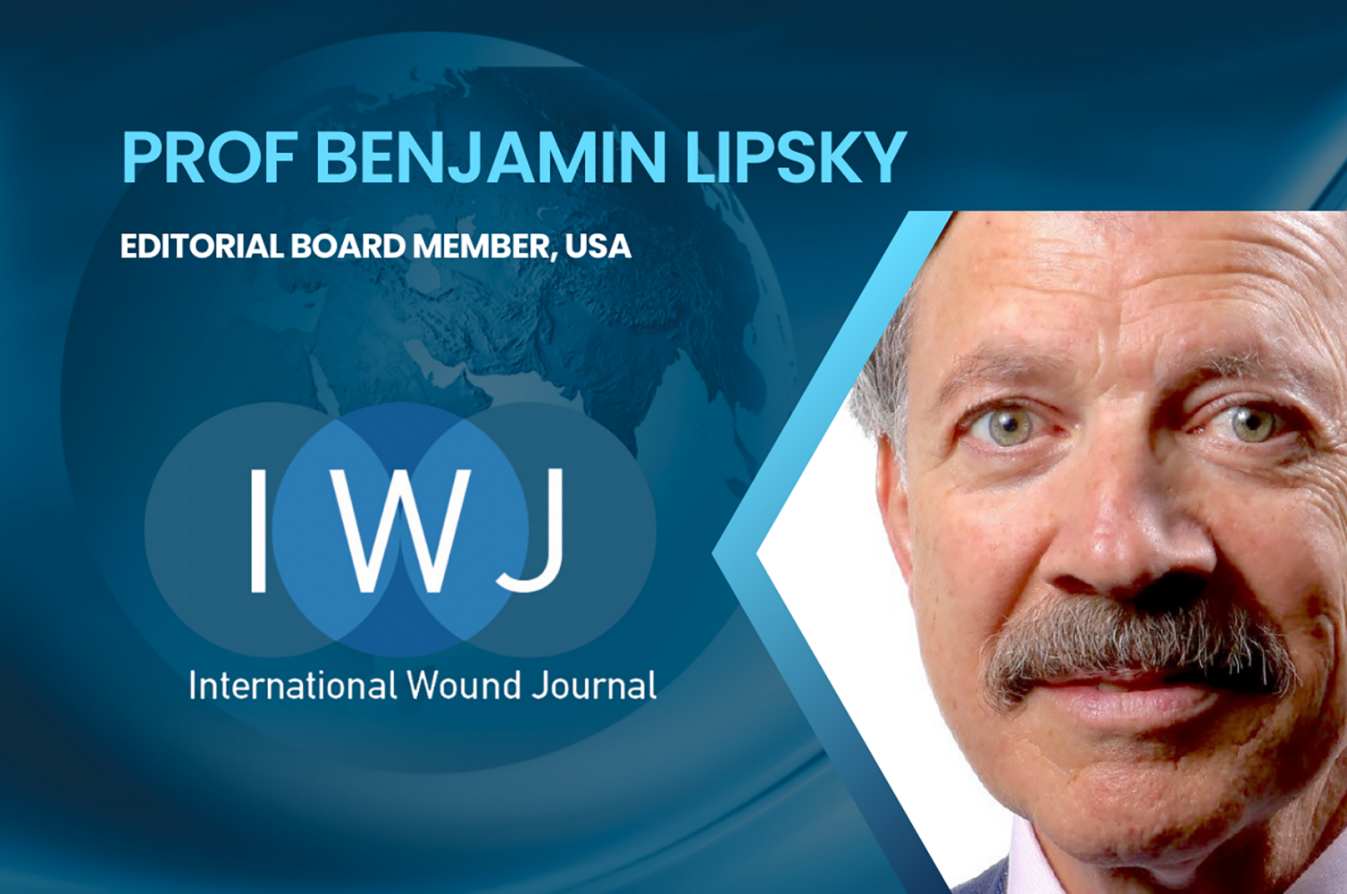

Benjamin A. Lipsky is a Professor of Medicine Emeritus at the University of Washington, where he specializes in infectious diseases. At the affiliated Veterans Affairs Medical Center he was Chair of Infection Control, Hospital Epidemiologist, Director Primary Care Clinic and directed a Wound Infection Research Clinic that conducted >60 randomized clinical trials. He has published >315 peer‐reviewed papers, >100 additional papers/chapters and two medical books. He has been a Visiting Professor of Medicine at the University of Oxford and the University of Geneva. He chaired the guideline committees on diabetic foot infections of both the Infectious Diseases Society of American and the International Working Group on the Diabetic Foot from their inception until 2020. Among the awards he has received are: the Diabetic Foot Global Conference's ‘Edward James Olmos Award’, The American Diabetes Association's ‘Roger Pecoraro Award’, the International Symposium on the Diabetic Foot's ‘Karel Bakker Award’ and MedStar Georgetown University's ‘Distinguished Achievement Award in Diabetic Limb Salvage’.

**Dr Ana Manzotti, Mexico**

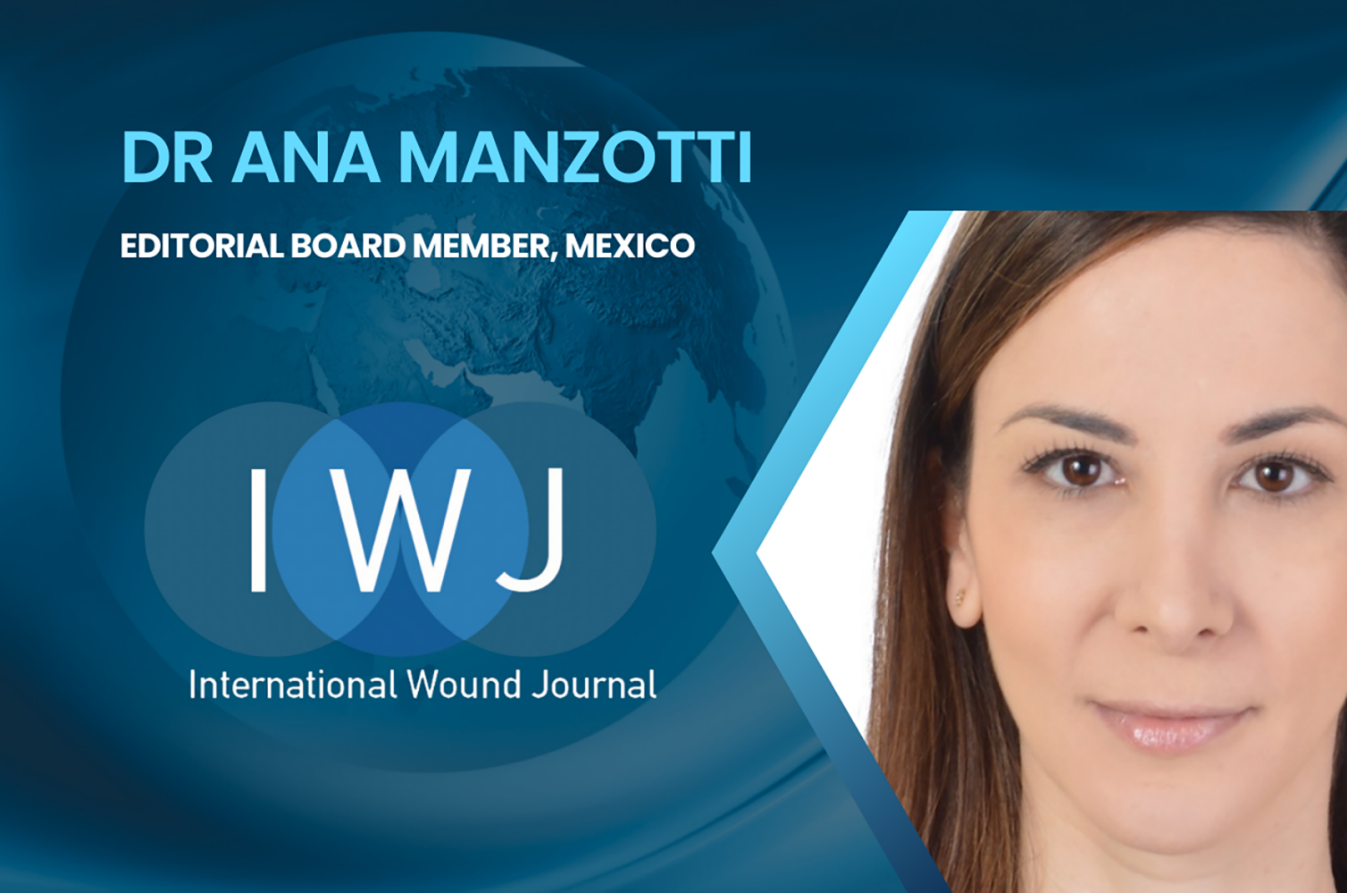

Dr. Ana Manzotti is a dermatologist in Mexico. While completing her training in dermatology in Guadalajara, Jalisco she discovered the field of wound care and later went on to complete postgraduate studies in advanced wound and stoma care at Universidad Panamericana in Mexico City. She has a special interest in wounds related to dermatological conditions and in exploring the contributions that dermatology can make to multidisciplinary wound care teams. She has a private practice in Monterrey, Mexico specializing in clinical dermatology and advanced wound care.

**Dr Perry Mayer, Canada**

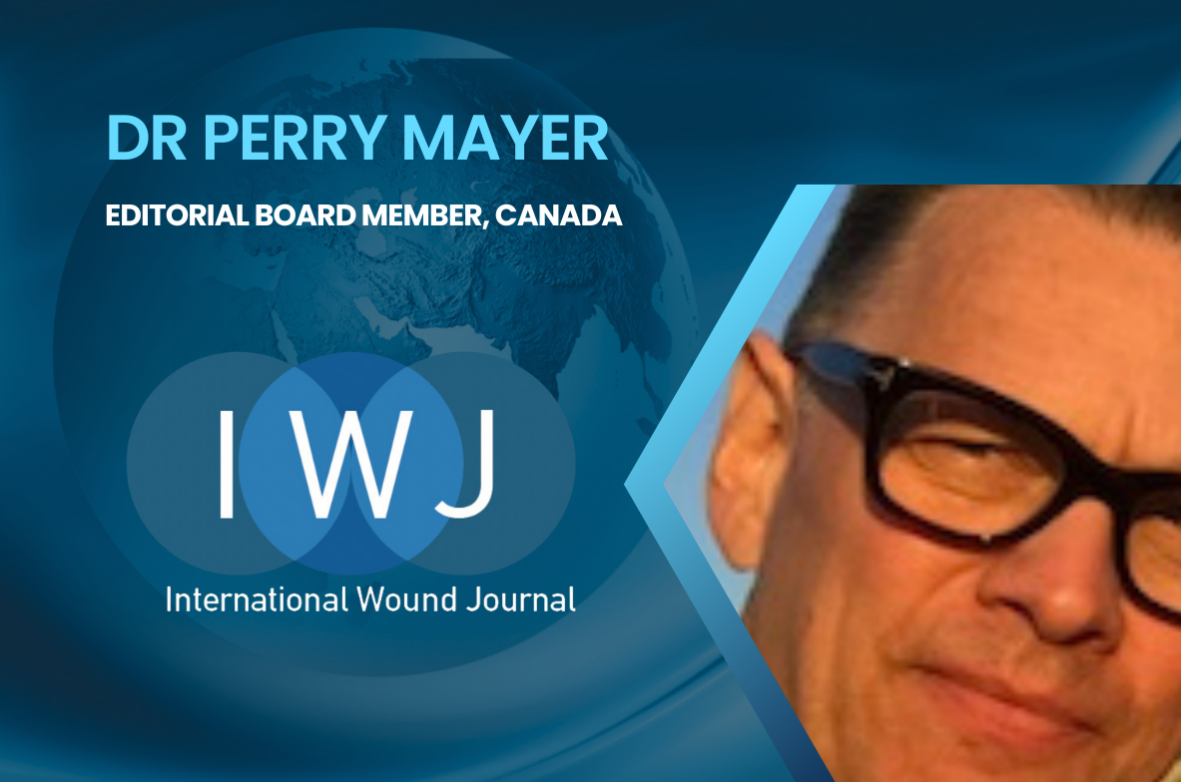

Dr. Perry Mayer is the Medical Director of The Mayer Institute (TMI), a centre of excellence dedicated to the treatment of the diabetic foot. He received his undergraduate degree from Queen's University in Kingston, Ontario, Canada and medical degree from the Royal College of Surgeons in Ireland. He returned to Kingston in 1993 to practice family medicine and establish the Quarry Foot Clinic. In 2003, Dr. Mayer moved to Hamilton, Ontario and concentrated his time solely on the treatment of the diabetic foot. He formed The Mayer Institute in 2006. In the past year, TMI had over 16,000 patient visits, with over 14,000 visits being for diabetic wound care. Dr. Mayer's current research projects include a trial investigating the effect of a novel delivery system that transports various molecules through skin and wound tissue and examining the effects of this transport system on the proliferative and cell migration phases of wound healing, control of neuropathic pain and most recently for local anaesthesia prior to surgical wound debridement.

**Professor James McGuire, USA**

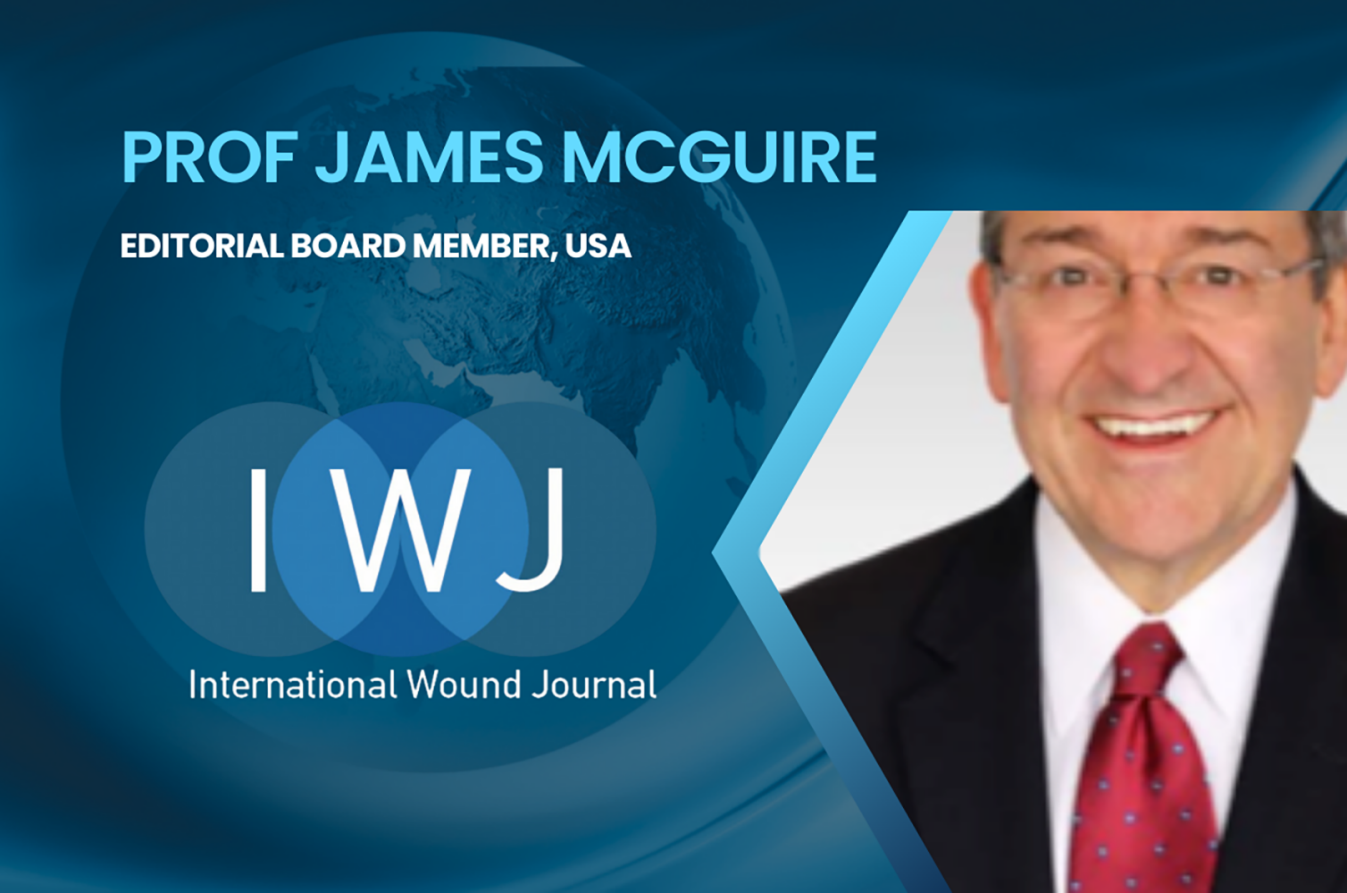

DPM, LPT, LPed, FAPWHc is the Director of the Leonard Abrams Center for Advanced Wound Healing and a Clinical Professor in the Departments of Podiatric Medicine and Biomechanics at the Temple University School of Podiatric Medicine, located in Philadelphia, Pennsylvania.

**Professor Gerit Mulder, USA**

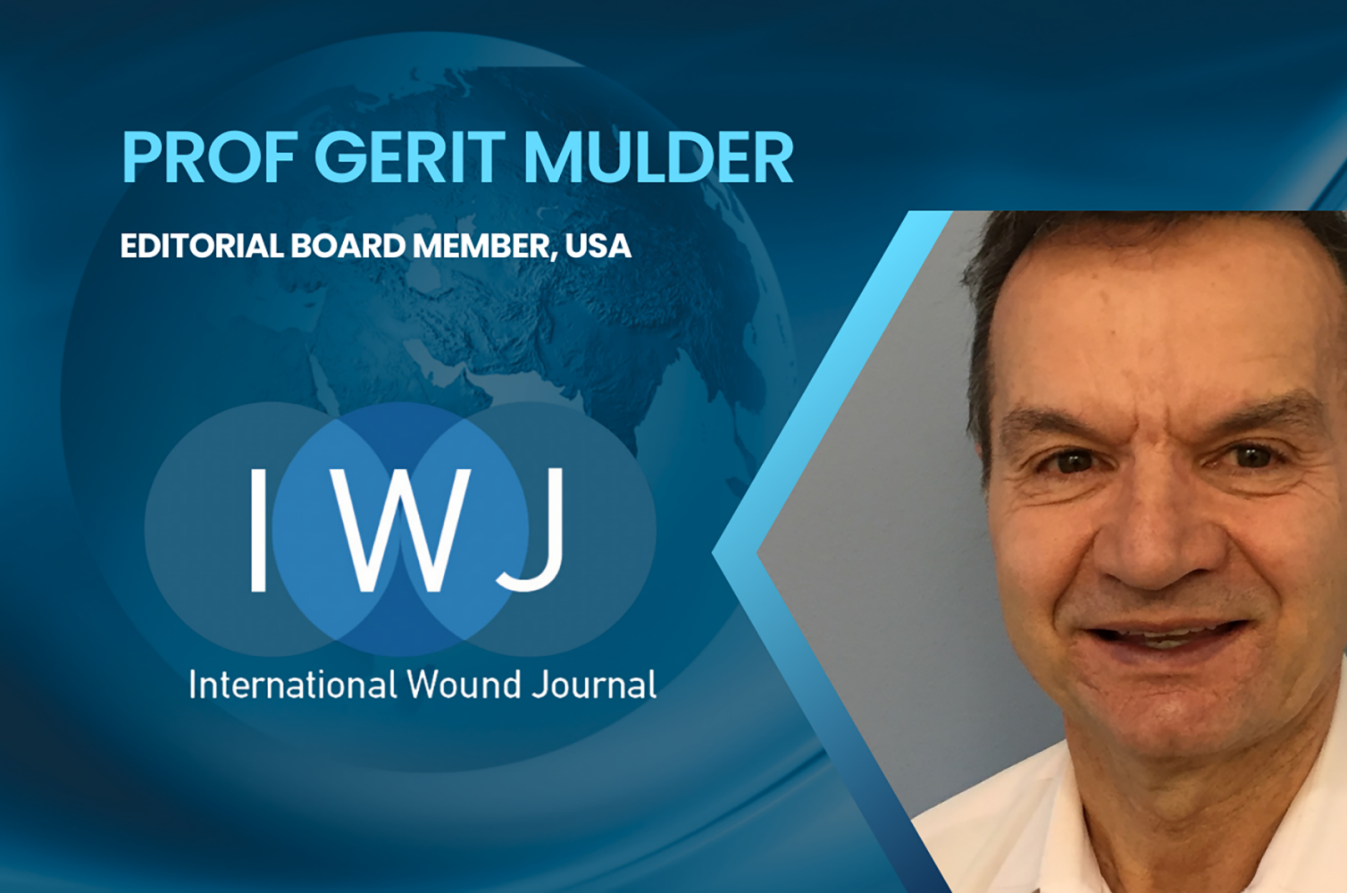

Dr. Mulder has over 41 years of experience in all aspects of wound care, including research, clinical and academic aspects. He has published over 200 publications, has been a Principal Investigator on our 100 research projects and has lectured internationally, helped open numerous wound clinics and conducted research on almost every continent. He was and still holds the title of Professor of Surgery and Orthopaedics at the University of California, San Diego. He is well recognized as a global expert in wound repair and regeneration.

**Dr Nancy Munoz, USA**

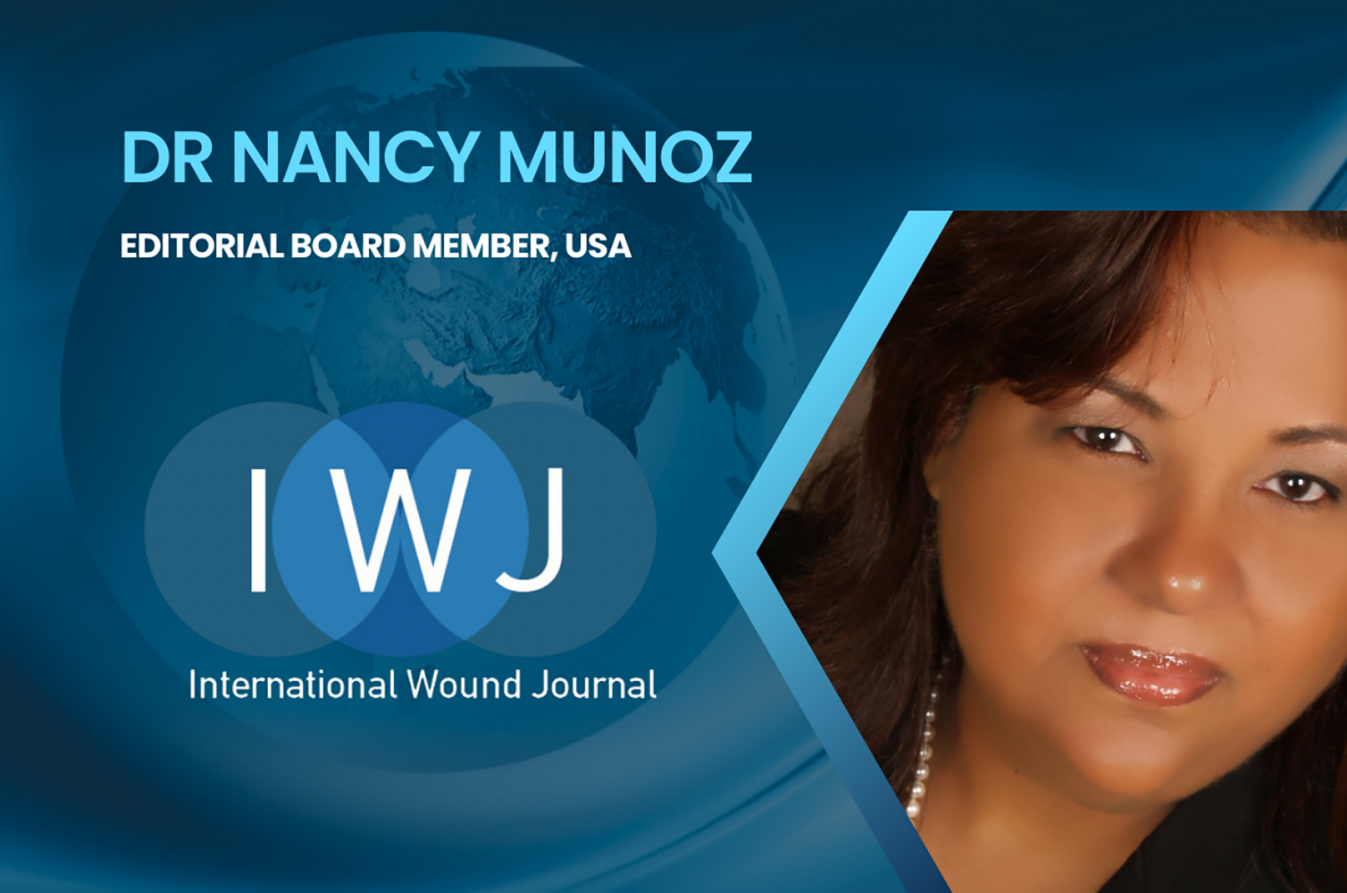

Dr. Nancy Munoz holds a Doctorate in Clinical Nutrition from Rutgers State University of New Jersey, a Master in Healthcare Administration from the University of Maryland and a Bachelor in Food and Nutrition from Marymount College in New York. Teaching healthcare practitioners the role of nutrition as a modifiable risk in the development of pressure ulcers is at the core of her practice. While the nutrition care of older adults has defined her career, she now provides services to the Veterans that have served our Nation. Dr. Munoz is a lecturer for the University of Massachusetts Amherst Nutrition Department and the Assistant Chief for Nutrition and Food Service at the Southern Nevada VA Healthcare System. Dr. Munoz has authored and served as expert reviewer for books and manuscripts for numerous professional publications, and the Academy of Nutrition Evidence Analysis Library. As a registered dietitian nutritionist and a member of the Academy of Nutrition and Dietetics, Dr. Munoz has a long history of professional service. She has served as past treasurer and president for the New Jersey Dietetic association, as well as a member of the Academy's Positions Committee. She currently serves as the Professional Development Chair for the Dietetics in Healthcare Communities DPG and as a Director on the board for the National Pressure Ulcer Advisory Panel.

**Professor Bijan Najafi, USA**

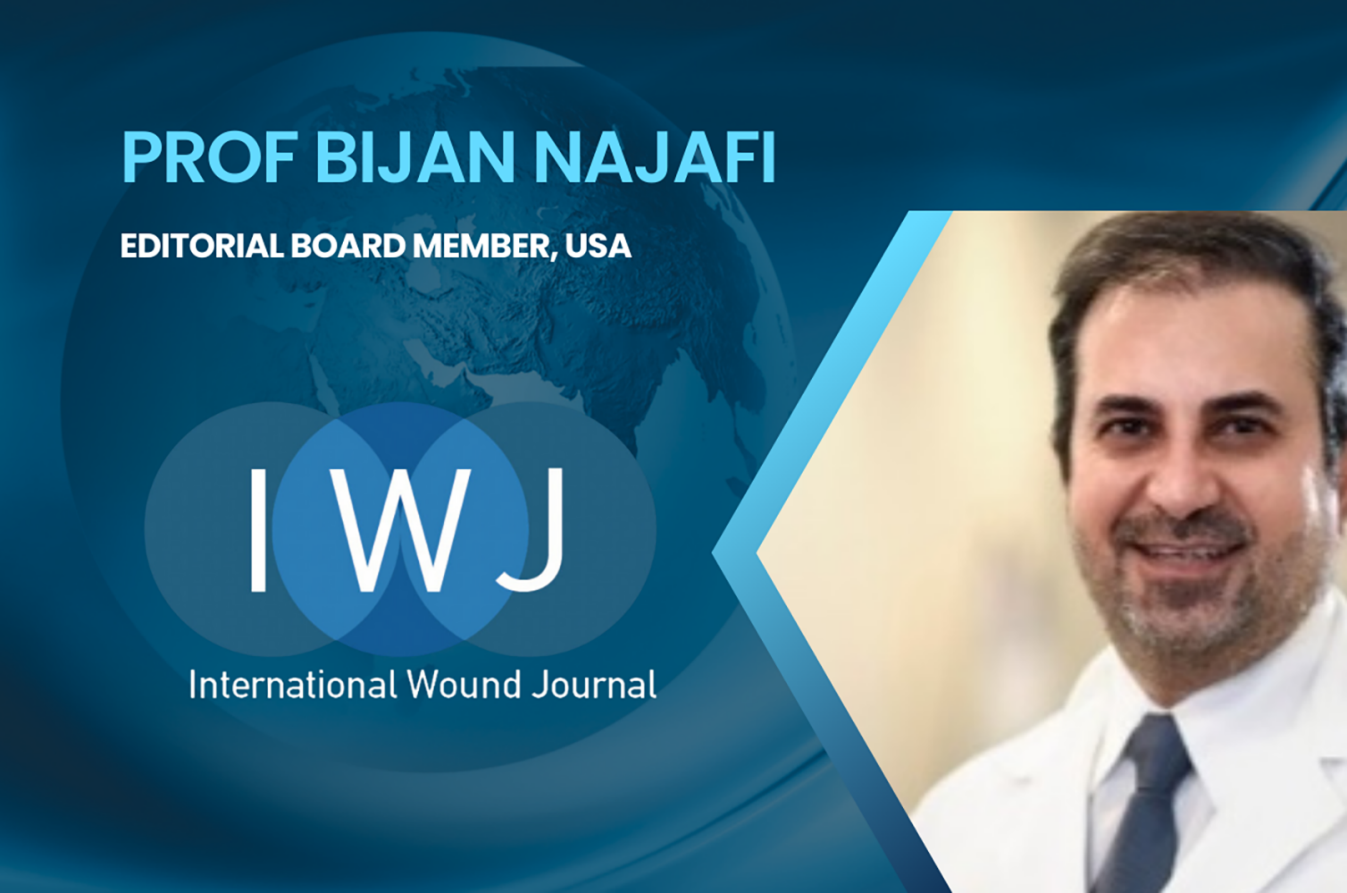

Dr. Bijan Najafi, a Tenured Professor of Surgery at Baylor College of Medicine, specializes in digital health and biotechnologies. He serves as the Director of Clinical Research in Vascular Surgery and as the Co‐Director of the Center to Stream Healthcare in Place (C2SHIP). Recognized by Tucson Local Media in 2014 as a top health leader, he earned his Ph.D. in bioengineering from EPFL and completed a postdoctoral fellowship at Harvard. Najafi has published over 250 articles, accumulating more than 16,000 citations. Ranked in the top 1% by Expertscape in 2021 in his field of research, he was inducted into the AIMBE College of Fellows in 2023 for his contributions to digital health technologies in prevention.

**Dr Daria Narmoneva, USA**

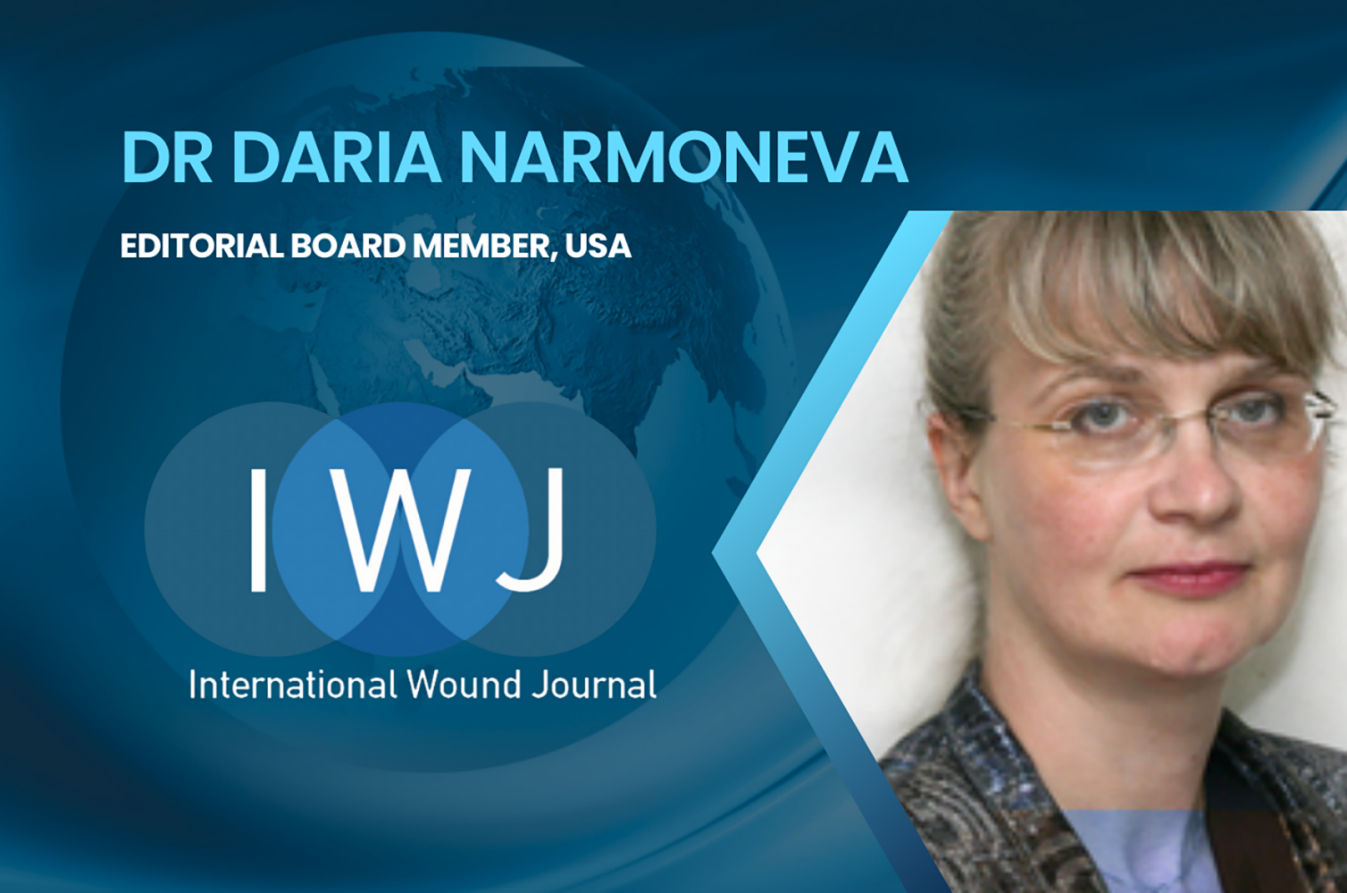

Chronic diabetic ulcers are the leading cause of non‐traumatic limb amputations in the United States and are a significant healthcare burden. To improve the healing of chronic diabetic wounds, we created a unique microenvironment using a novel hydrogel (based on self‐assembling peptide nanofibers) that mimics the native matrix in the wound and promotes healing. To activate unresponsive, diseased cells within the chronic wound, we have developed an electric field‐based technology that uses high‐frequency wireless electric field stimulation to activate capillary cells and enhance blood vessel formation in the wound, which results in much faster healing. This technology has been successfully tested in the mouse and pig models and is under active translational development in collaboration with UCRI and the UC Office for Entrepreneurial Affairs and Technology Commercialization.

**Dr Ann Marie Nie, USA**

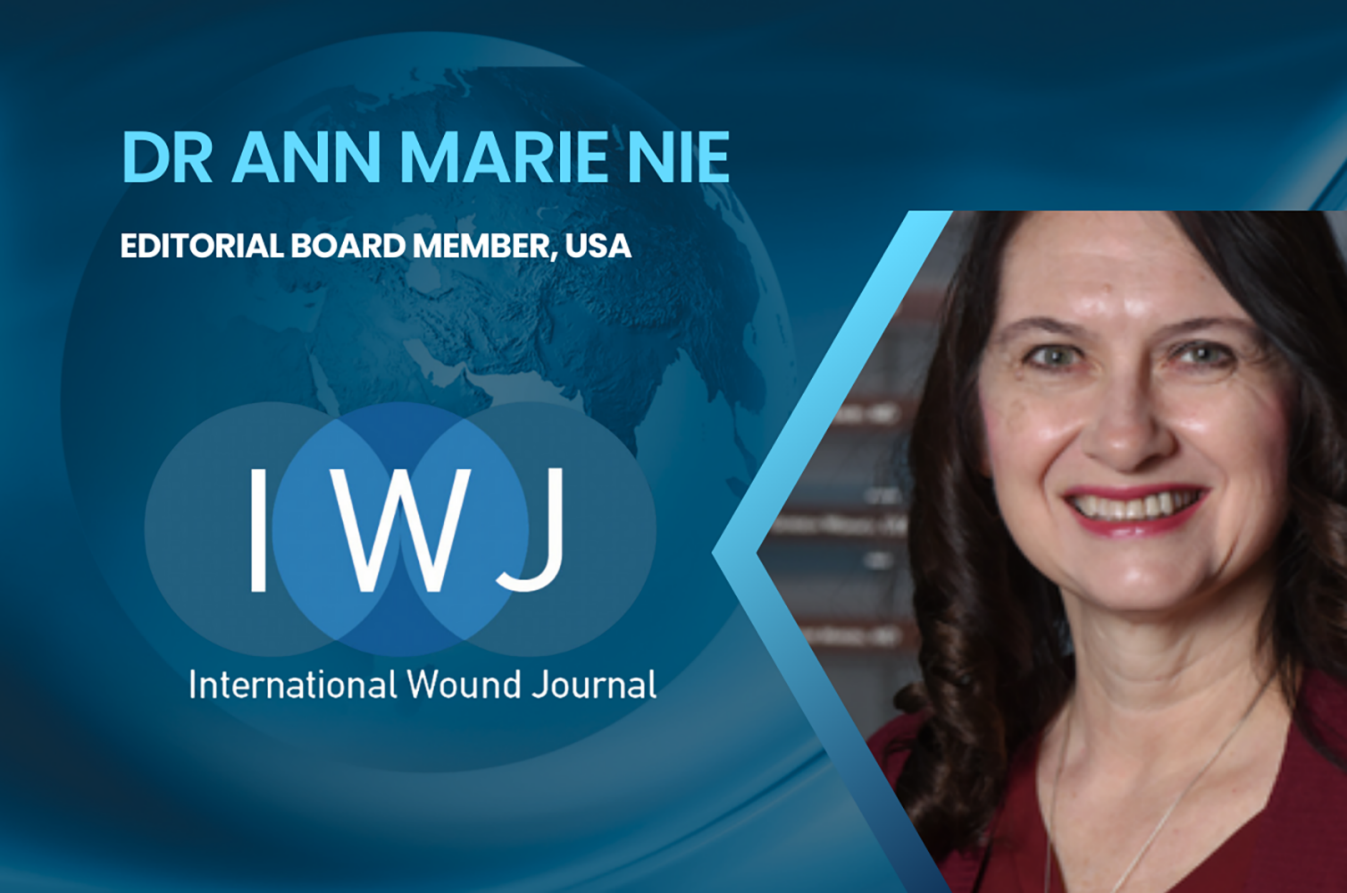

Ann Marie Nie is a globally recognized expert in wound, ostomy and pressure injury prevention. As a member of the paediatric surgery division at Dayton Children's Hospital. She works collaboratively with members of the inpatient care to prevent pressure injuries.

The Cincinnati native received her Bachelor of Science in Nursing at the College of Mount St. Joseph University in Cincinnati, Ohio. Following college, Ann Marie completed her certification in the Wound, Ostomy and Continence Nurses Society at Emory University School of Medicine. She then went on to obtain a Master of Science in Nursing, Family Nurse Practitioner at Northern Kentucky University. Ann Marie is now in the process of completing her nursing Ph.D. at The Catholic University of America.

Ann Marie chose to specialize in wound, ostomy and continence treatment because healing a wound gives individuals a sense of ‘being whole’. When there is an open area of the skin, it greatly affects a person's sense of self. Preventing pressure injuries not only helps the hospital, but it assists the patient in not having to deal with a wound that was created during hospitalization.

Her favourite thing about working with kids is how open and honest they are, and how much they love life!

**Simone McConnie, Barbados**

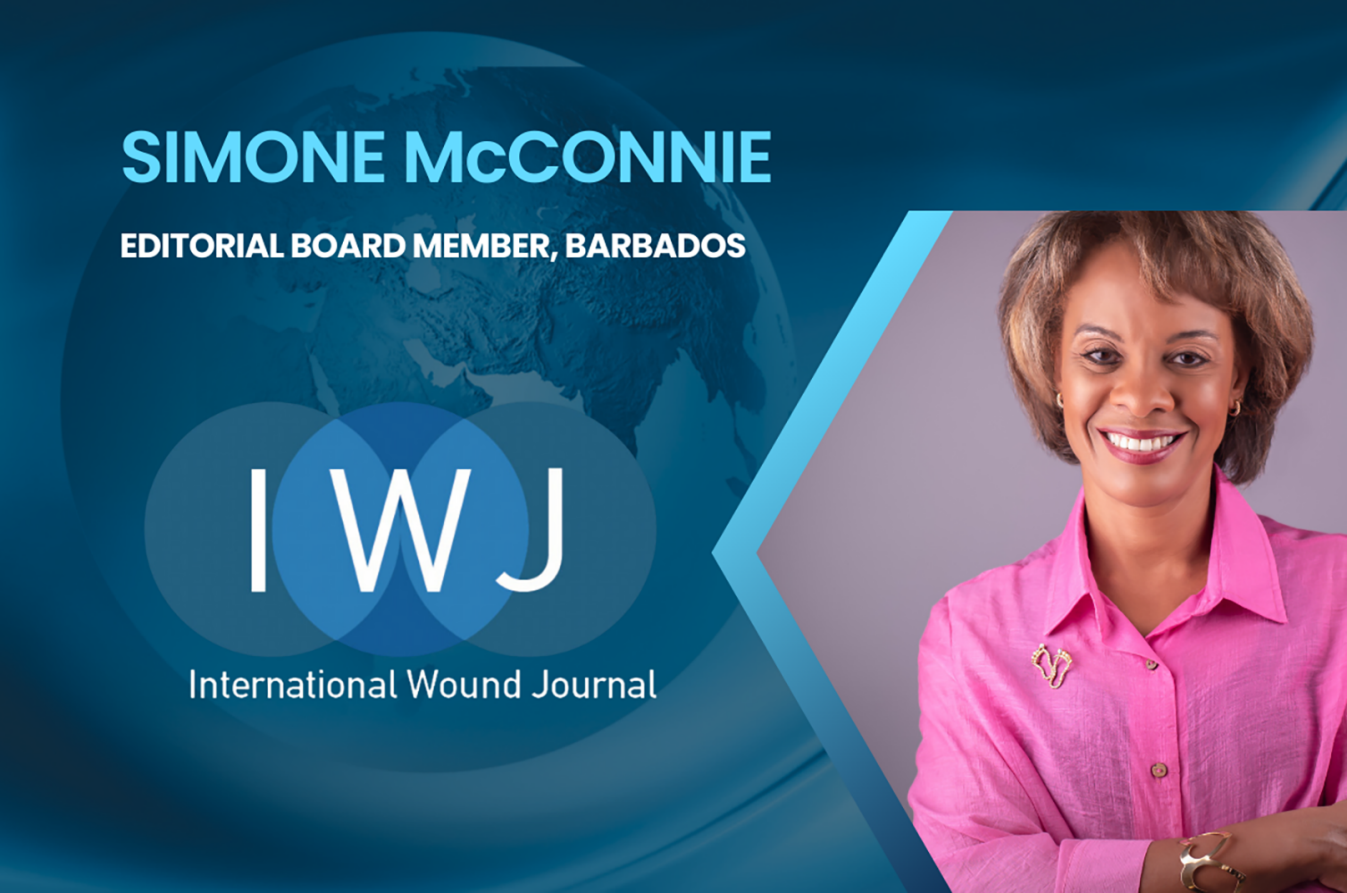

Simone was inspired to be a Podiatrist, as a call for Podiatrists from the Health Ministry in Barbados, due to high amputation rates. Little did she know the diabetic foot, wound care and foot biomechanics would become her passion. Podiatrists, play a vital role in limb salvage and foot health. Qualified from the University of Westminster with a BSc in Podiatric Medicine in 1993 she has been working in Barbados and then region since 1994, after a small research opportunity at King College Hospital in London, home of the first diabetic foot clinic in the United Kingdom. She worked as a research assistant of Alethea Foster who went on to be her mentor in the Diabetic foot upon her return to Barbados. She also holds her MBA from Durham University in 2011.

She went on to do specialist training in the field of diabetes and has worked extensively with the International Working Group on the Diabetic foot (now known as D‐foot International) in association with the Rotary Club of Ledbury, United Kingdom and local Rotary Club of Barbados South, in implementing the ‘step by step’ programme across the region, A founding member, and past Trustee at the Barbados Diabetes Foundation, located in the Maria Holder Diabetes Center for the Caribbean, she worked with a local team of Podiatrists to establish the first Diabetic Foot clinic in Barbados under the roof of the Center. She continues to represent Barbados on D‐foot International and is co‐chair for the North American and Caribbean region. She is a member of the American Podiatric Medical Association, College of Podiatrist, and is registered both in Barbados and United Kingdom with the Health Professions council. President of the Barbados Association of Podiatrist and founder of save our soles trust—a charity dedicated to education and advocating on behalf of diabetic patient. Founder and Managing Podiatrist of Comfeet footcare clinic. And a founding member of the international members group of SOCAP, she also continues to work with many other organizations to build capacity and advocate for the Podiatry profession. She has received the Oscar Jordan Award for your work with the Diabetic foot clinic, an Award from the Diabetes Foot centre group and most recently recognized by the Board of D‐ Foot international a commendation award for her work in the region and support of the D‐foot programmes. She has written extensively and appeared in local newspapers, magazines, on local television and other media outlets educating and promoting Podiatry and diabetes. She continues to develop clinical skills by attending annual seminars and conferences relevant to her practice.

**Professor Samuel Nwafor, USA**

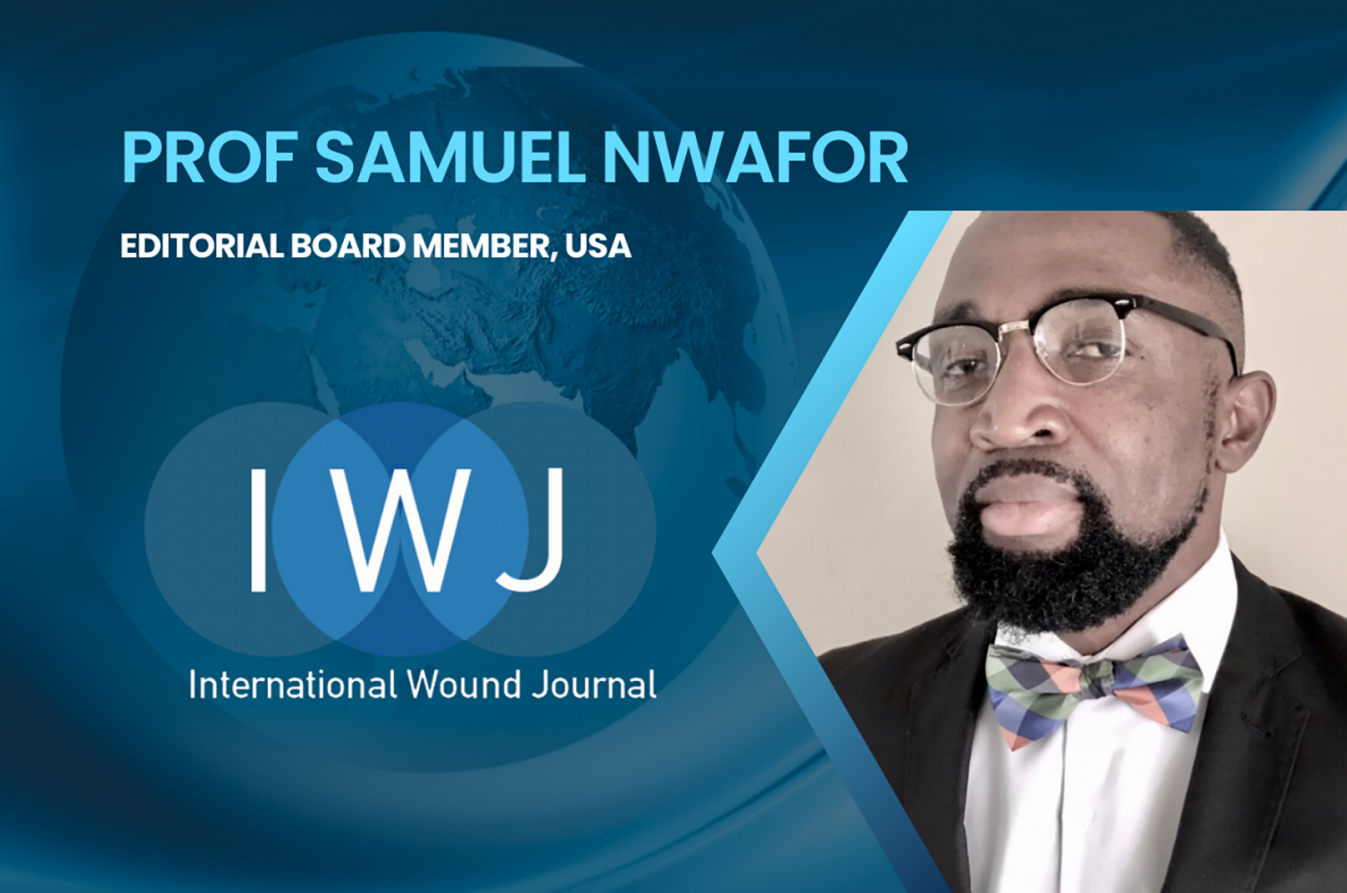

T. Samuel Nwafor is Chief of Staff and Medical Director at Promise Hospital in Phoenix, AZ. He is also a board‐certified wound and hyperbaric medicine specialist at Tempe St Luke's Wound & Hyperbaric Medicine Center in Tempe, AZ. He is the Chief of Staff & Medical Director of Promise Hospital in Phoenix AZ, USA.

He is the Founder and Chief Medical Officer of Hannah Medical Institute LLC and an Adjunct Clinical Professor of Medicine at Midwestern University, Glendale, AZ, USA. Dr Nwafor has worked in various consulting and advisory roles to some of the largest corporations and organizations in the United States across the spectrum of academia, government and professional sports.

He brings his expertise, leadership skills and passion for humanitarian endeavours to the HPCI board.

**Dr Laura Parnell, USA**

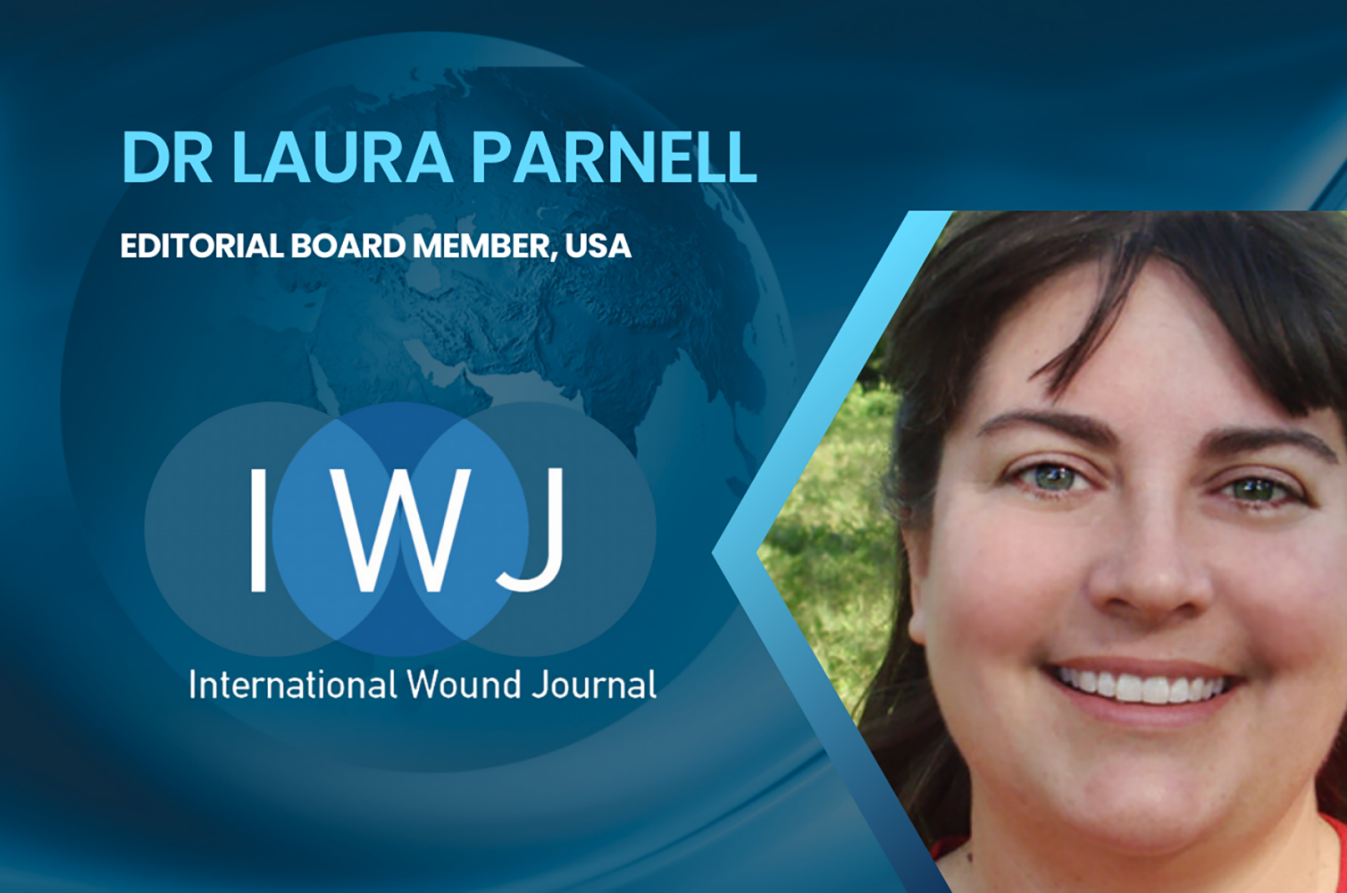

Laura K.S. Parnell founded Precision Consulting in 1998 and specializes in wound healing and burn research. Laura designs research protocols, develops scientific niche products and provides scientific knowledge, medical writing and regulatory background based on the needs and budget of her clients. She has an international clientele and has worked extensively with industry representatives and clinicians on research investigations.

Laura's career in wound healing began in 1990 at Texas A&M University while doing research in microbiology and immunology during graduate school. In 1992, Laura joined the Wound Healing Foundation. She was appointed to the Membership Committee in 1998. In 2006, Laura was elected to the first of two 3‐year terms on the Wound Healing Foundation Board of Directors, followed by a 1‐year term as a member at large. In 2012, she was elected to the Wound Healing Foundation Board of Directors and became President of the Foundation in 2013. Under her leadership, the Foundation updated its mission and has strived to broaden its fundraising efforts benefiting researchers and patients through awareness, education and research. Her personal goal is to see wound healing and associated problems be solved in more innovative and creative ways.

**Dr Laurie Parsons, Canada**

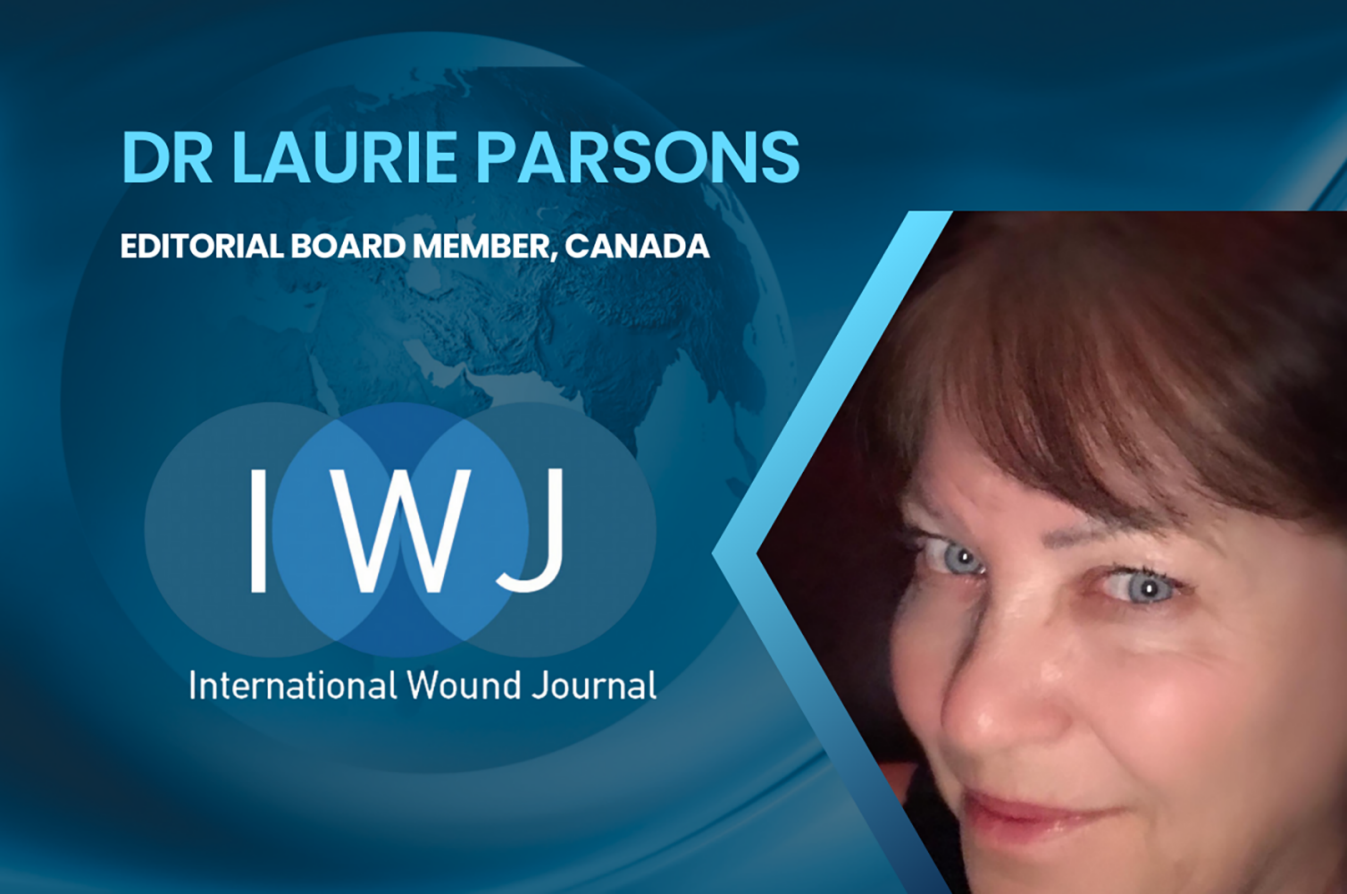

Dr Parsons is a Royal College trained dermatologist who has spent 25 years in the area of chronic wounds, contact dermatitis and general practice of skin disease. Originally a graduate of Memorial University of Newfoundland, she finished her post‐graduate training at the University of Ottawa. Currently, Dr Parsons is the Medical Director of the Calgary Zone Wound Clinic and Chair of the Dermatology Sub‐specialty Committee at the Royal College of Physicians.

**Professor Irena Pastar, USA**

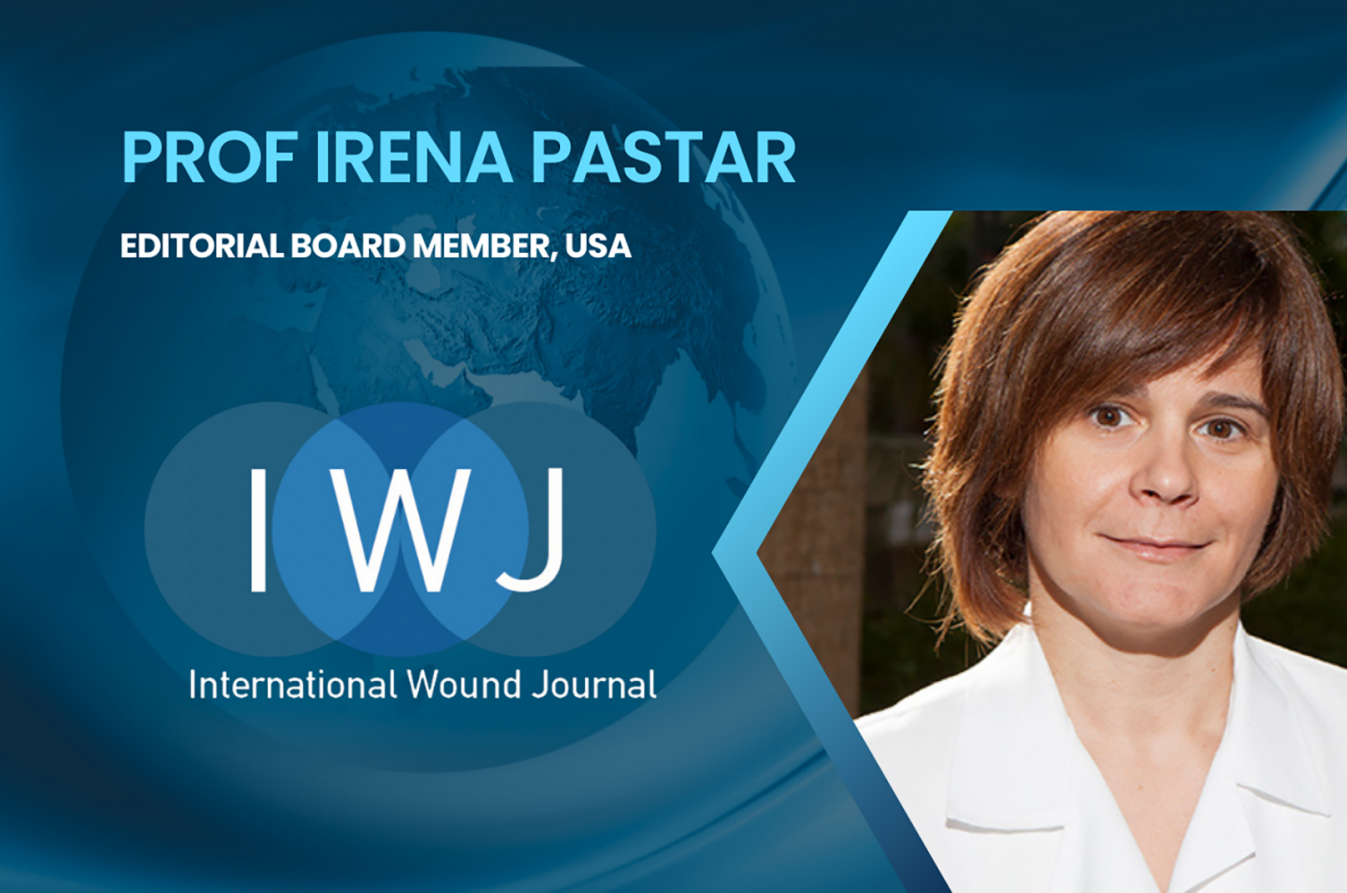

Dr. Irena Pastar received doctoral degree in molecular microbiology at the University of Belgrade, followed by postdoctoral trainings at The Rockefeller University and Cornell University Weill Medical College, Hospital for Special Surgery. She was an Assistant Professor at the New York University School of Medicine before joining the University of Miami faculty in 2011. Dr. Pastar's research focuses on molecular mechanisms of wound healing, cutaneous response to wound microbiome, mechanism of novel antimicrobial therapies and molecular pathology of chronic wounds, such as diabetic foot and venous leg ulcers. Dr. Pastar is leading multiple transnational projects on skin and wound infections funded by the National Institute of Health, US Department of Defence and Industry.

**Dr Harry Penny, USA**

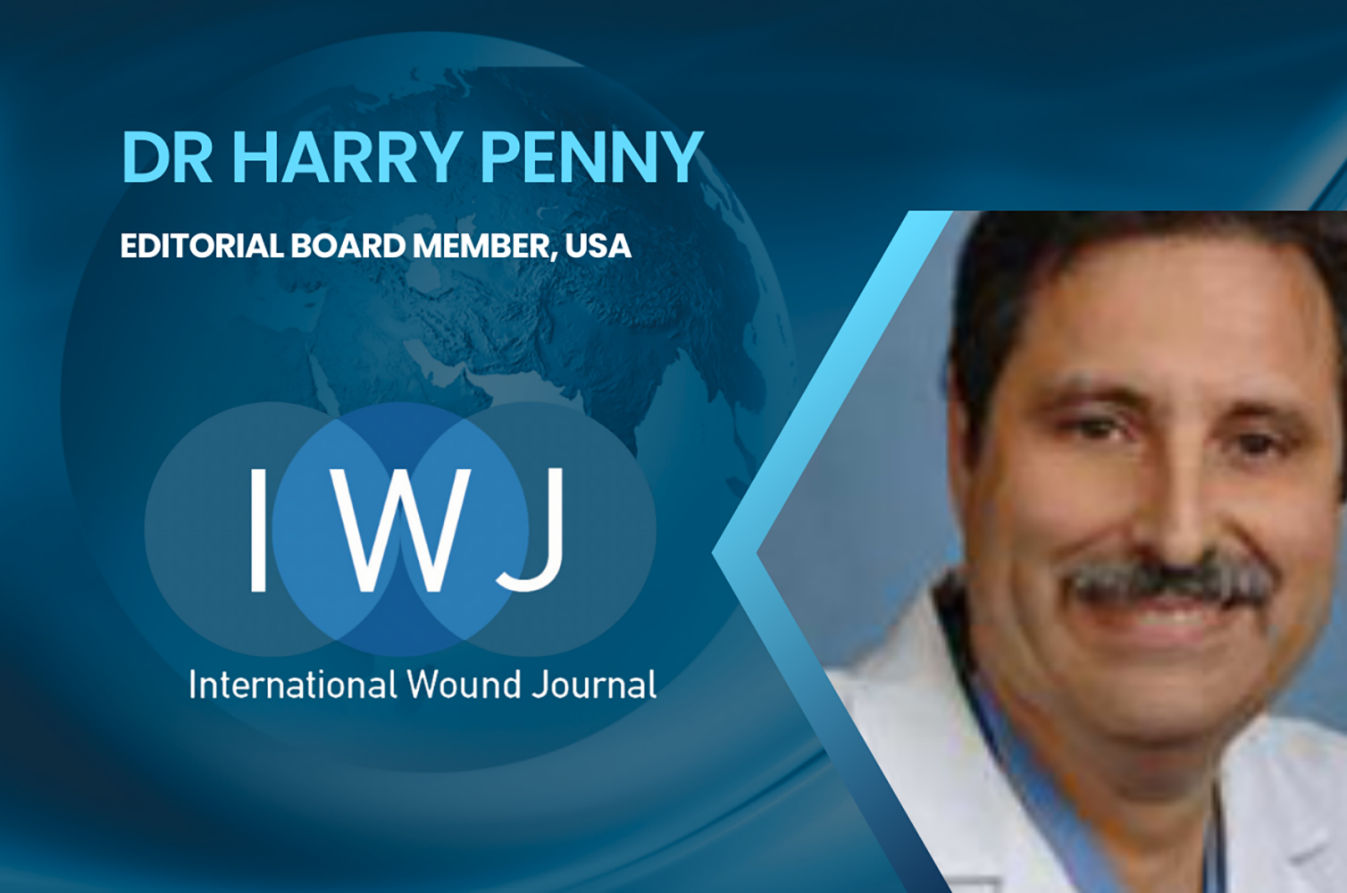

Dr. Harry Penny has published research articles in the Journal of Wound Care and serves on the editorial committee for that Journal. He has also published in the Journal of Foot and Ankle Surgery, Advances in Skin and Wound Care, Journal of American Podiatric Medical Association. He has served as Chairperson of the Penn State Altoona Hershey Medical Conference for the past 8 years for ‘Updates in Medicine’ and will again for the next conference.

He is a Clinical instructor for the University of Pittsburg Medical Center (UPMC) Altoona family practice programme past 19 years, an instructor for UPMC Altoona for ACLS past 14 years. Has developed a model mentoring programme with APWH for medical, podiatry, osteopathic and pa students for wound care. He serves as an Advisor to Penn State Hershey‐APWH newly formed student‐residency chapter and heads the BMA UPMC Altoona wound clinic.

Dr. Penny continues to be involved with clinical trials and is a Former Major US Army Reserves. He will be serving as Editor of a wound care book with Christian Brothers University of Memphis TN.

**Professor Anie Philip, Canada**

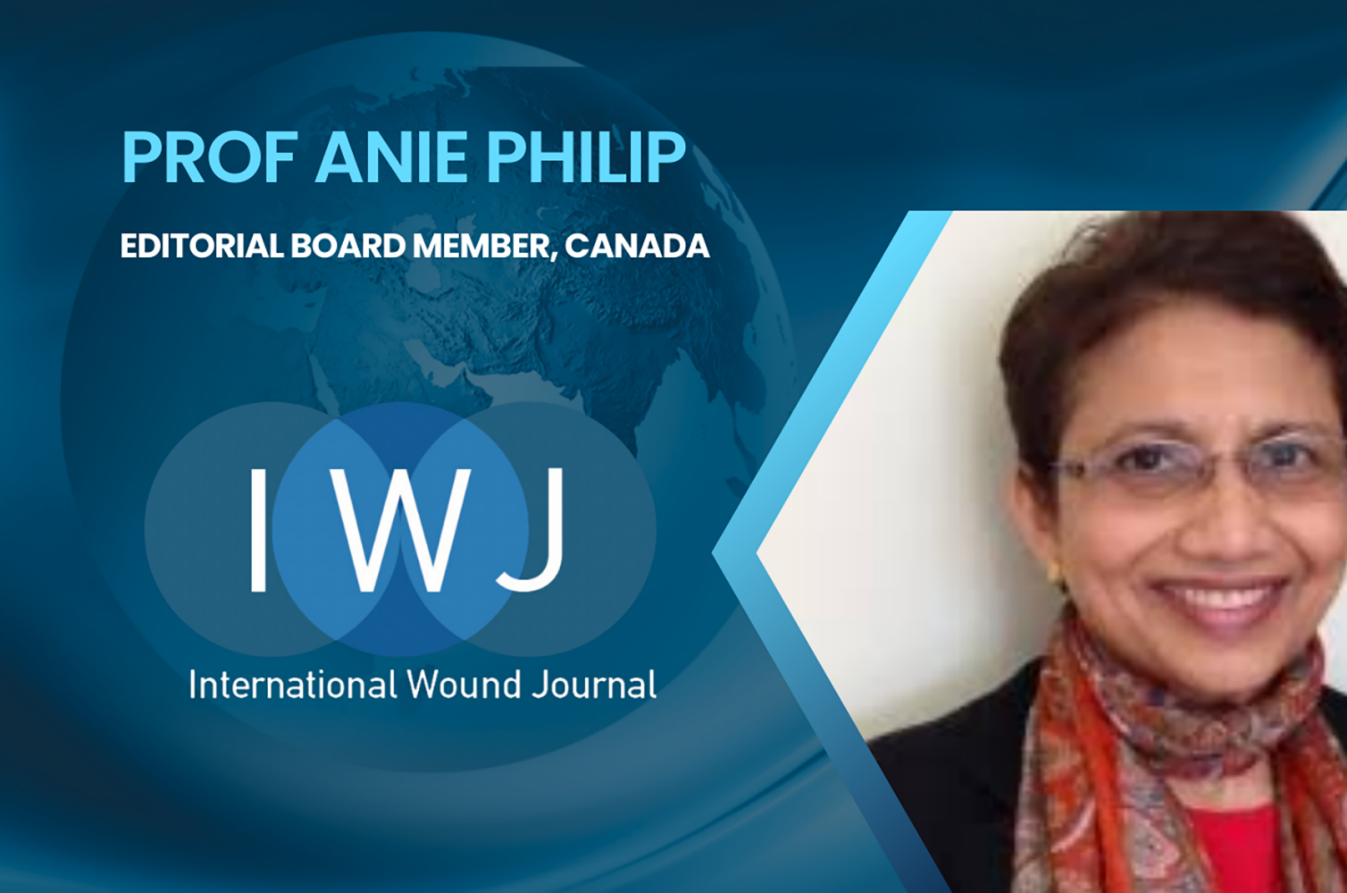

Anie Philip (PhD) is a full professor at McGill University and a Senior Scientist at the Research Institute of the McGill University Health Center (RI‐MUHC). She is currently the President of ‘Skin Research Group of Canada’. Dr Philip is also the Associate Director of the ‘Skin Investigators Network of Canada (SkIN Canada)’. Her most significant contributions include the discovery of CD109 as a TGF‐b co‐receptor and inhibitor of tissue fibrosis, and the finding that CD109 is a promoter of tumorigenesis in squamous cell carcinoma. Her laboratory is supported by three CIHR Project Grants, a CIHR Network grant and an NSERC Discovery grant. She has been funded by other agencies including the Department of Defence (DOD), the Heart and Stroke Foundation and the Canadian Arthritis Network. Dr Philip collaborates with scientists at the National and International levels and holds grants as a co‐applicant with them (New Frontiers in Research fund, Canada, FRQS). Her laboratory has an excellent track record in the training of HQP and typically supervises as the primary supervisor 2‐3 PhD students, 2‐3 MSc students, 2 PDFs and 2 undergraduates/medical students.

**Professor Eleonor Pusey‐Reid, USA**

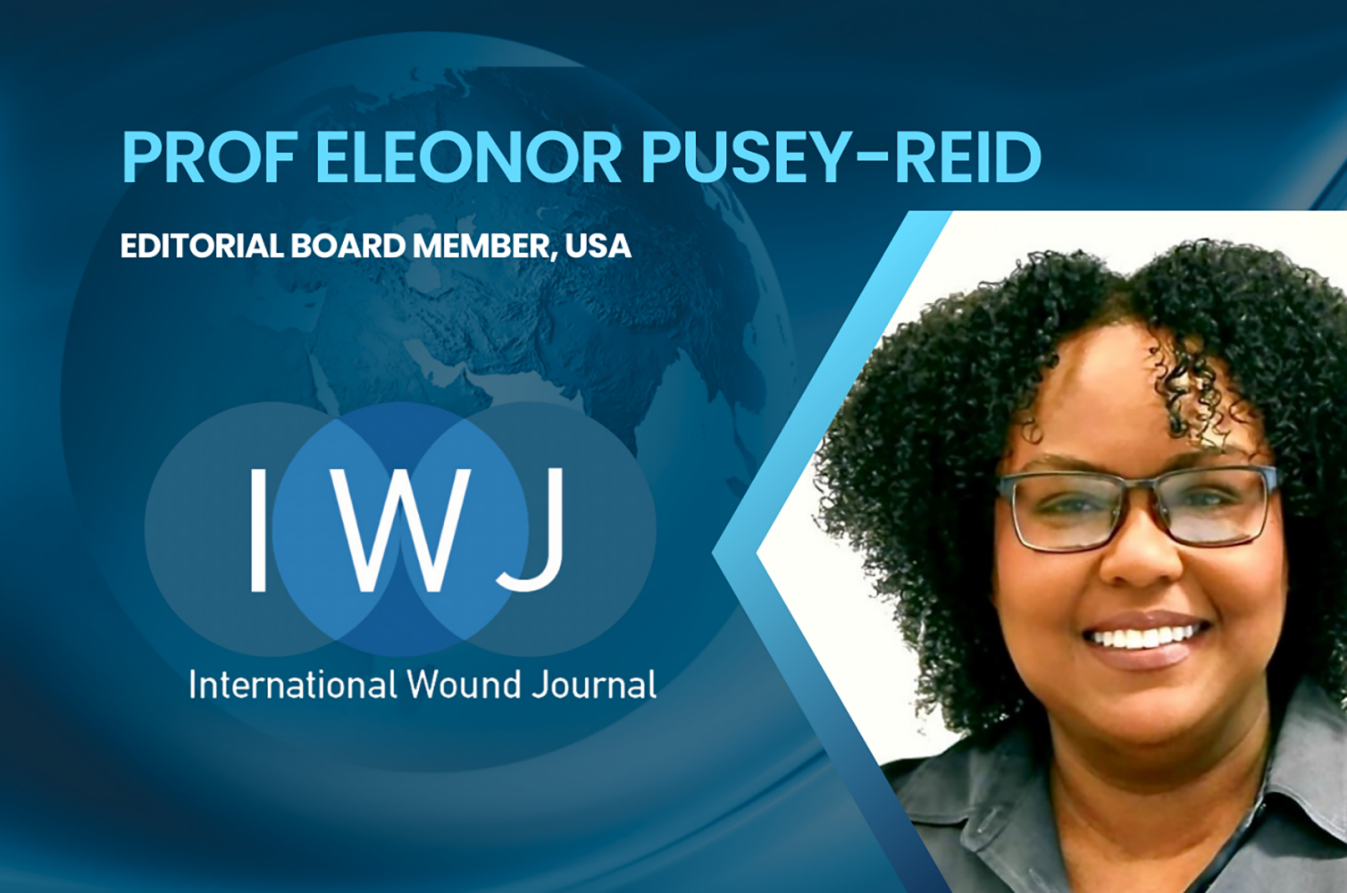

Dr. Eleonor Pusey‐Reid, is a distinguished Associate Professor at the MGH Institute of Health Professions School of Nursing (MGH IHP SON) with a career spanning over three decades. She has actively championed justice, equity, diversity and inclusion (JEDI) in the academic and clinical spheres.

She served over 4 years on the Massachusetts Board of Registration in Nursing, representing prelicensure education, and currently co‐chairs the School of Nursing JEDI Collaborative. Additionally, Dr. Pusey‐Reid contributes her expertise to the American Association of Colleges of Nursing's Diversity, Equity, Inclusion Leadership Network (DEILN).

Internationally, she has had a significant impact through a 4‐year tenure in Costa Rica, where she led and taught in a 4‐year nursing programme. She has also served as a visiting professor in Mexico and the Dominican Republic, focusing on critical care nursing in academic and clinical settings.

Dr. Pusey‐Reid's research addresses equity, intersectionality and justice in nursing education and practice. Her work on assessing dark skin tone (DST) in healthcare aims to improve provider competency and patient outcomes. She champions inclusive educational practices by examining how DST is represented in nursing textbooks and teaching materials, ensuring they reflect the diversity of the healthcare workforce and their clients.

In recognition of her efforts, Dr. Pusey‐Reid was awarded the Women of Colour Excellence in STEM Education Award in 2021 and the inaugural MGH Institute of Health Professions JEDI Award in 2022, underlining her profound influence on the field.

**Professor Eduardo Quintero, Mexico**

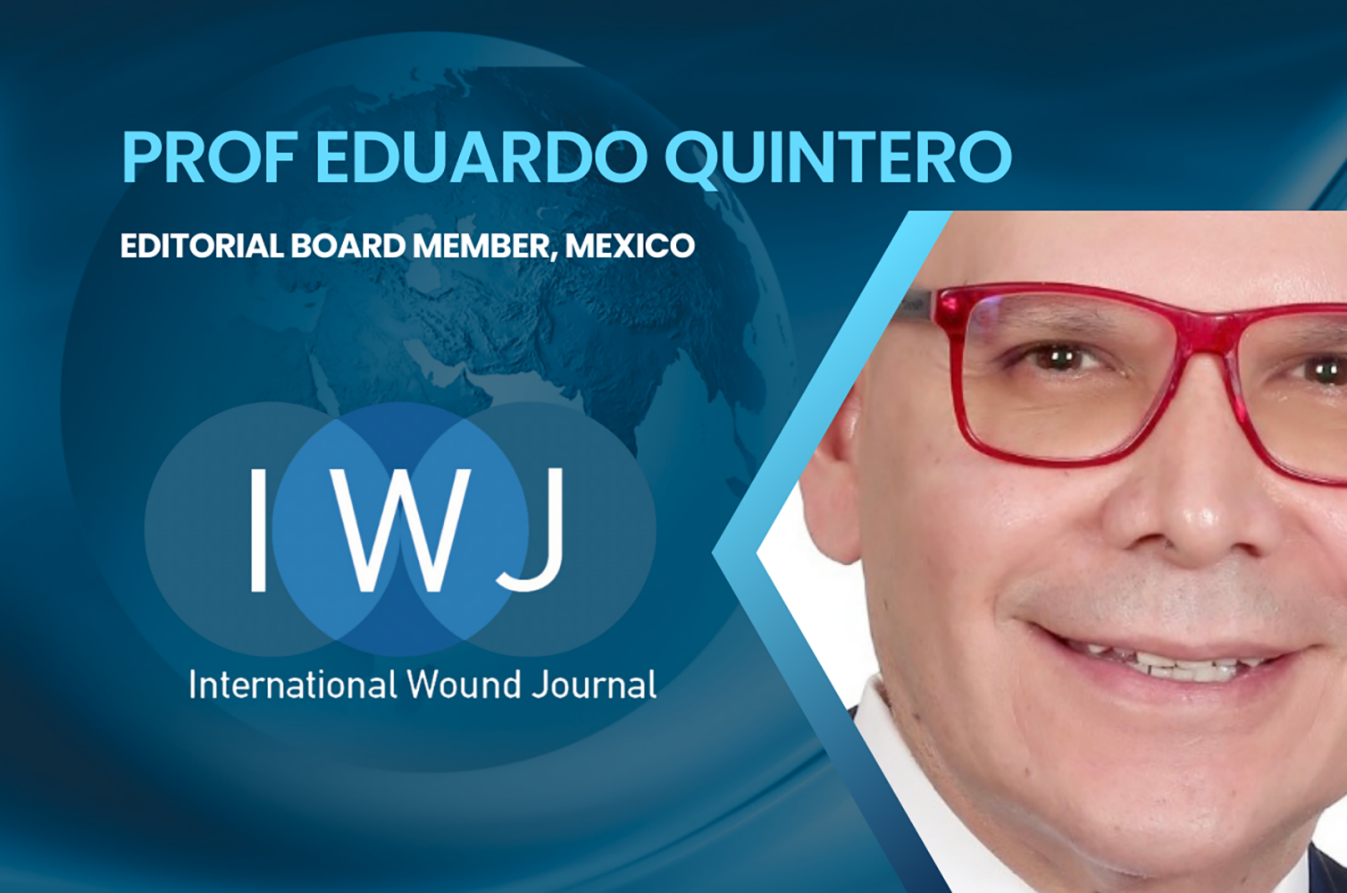

Professor Quintero graduated as a General Surgeon in 2002 and Plastic and Reconstructive Surgeon in 2005. He also received a diploma in advance wound care and stoma from UNAM.

His clinical career has been dedicated to the care, education and investigation of advance wound care and stoma in Mexico and Latin America.

He is an associate professor and part of the staff of the burn unit at ‘Centro Medico Nacional 20 de Noviembre’ in the education of the residents in plastic and reconstructive surgery.

Prof Quintero is a Past President of the Mexican Association for Wound and Healing Care and is currently their Head of international affairs. He was a Member of the continental board for Latin America for the WUWHS.

Prof Quinteri is a Member of the Journal of Wound Care editorial board (south America) and a Member of the Journal of Wound Care LATAM editorial board.

He has supervised multiple thesis students and has extensive publications in indexed journals. Hs is also an active member of different national and international associations of wound care and plastic and reconstructive surgery.

**Professor Piul Rabbani, USA**

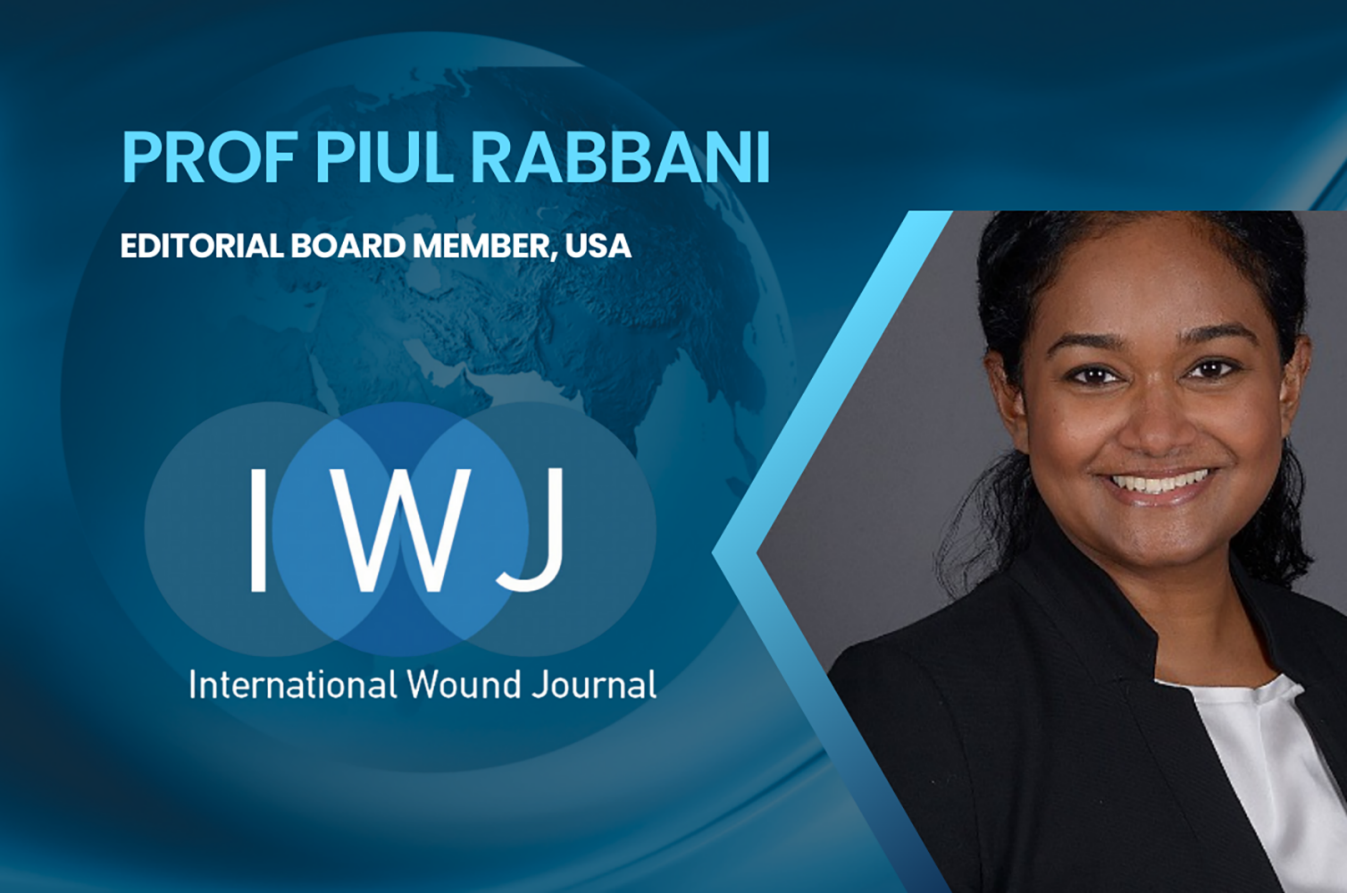

Dr. Piul Rabbani is an Assistant Professor in the Hansjörg Wyss Department of Plastic Surgery at New York University School of Medicine. Her lab's research programme is focused on the identification of intercellular communication mechanisms that are deficient in chronic non‐healing diabetic cutaneous wounds, with a particular interest in compensating with mesenchymal progenitor cell populations. Dr. Rabbani's lab is investigating extracellular vesicles as mediators of cellular conversation and exploring means of maximizing the therapeutic potential of extracellular vesicles harvested from adult bone marrow‐derived multipotent stromal cells. She is also involved in incorporating exosome‐based approaches with biomaterials to develop translatable delivery vehicles. Dr. Rabbani did her post‐doctoral work with Dr. Daniel Ceradini on multipotent stromal cells, their metabolic regulation and their application within diabetic wound healing. She did her graduate work with Dr. Mayumi Ito, investigating the co‐regulation of melanocyte and hair follicle epithelial stem cells.

**Professor Catherine Ratliff, USA**

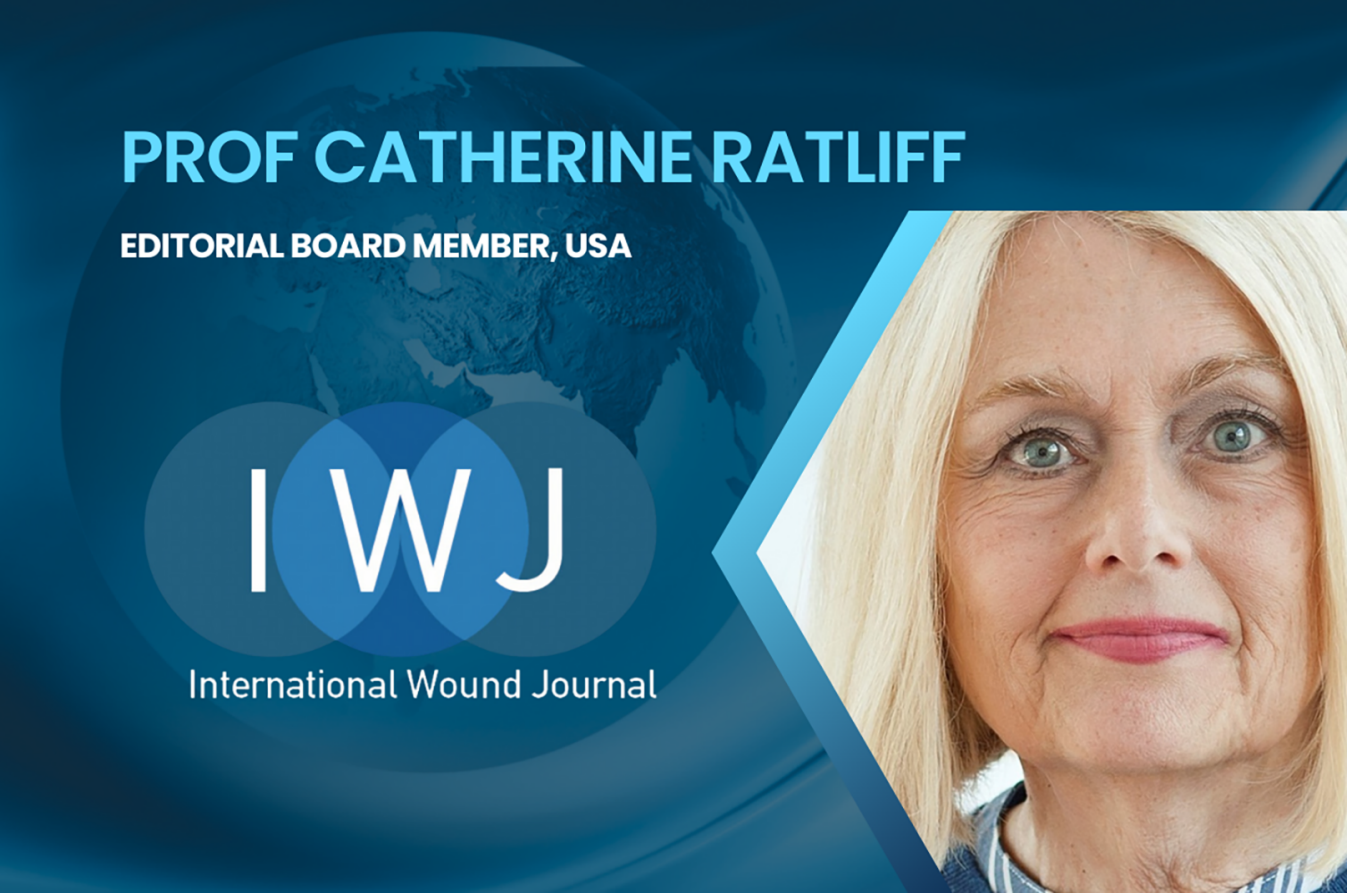

Dr Ratliff is a PhD prepared nurse practitioner with over 20 years of wound care experience and research. She is currently employed as a nurse practitioner at the University of Virginia where she has run a chronic wound care clinic there for over 19 years. The most common diagnosis for her patients at the clinic is pressure ulcer. She also teaches at the school of nursing where she is an associate professor. She is the programme director for a wound ostomy continence programme at the nursing school which is part of the graduate (masters) programme; one of only two such programmes in the country. In 2007, she won the UVA award for quality improvement for her work with pressure ulcers. She has published over 90 articles including being instrumental in publishing several national guidelines on pressure ulcers. She has spoken at many national meetings on the topic of pressure ulcers. She is also a past board member of the National Pressure Ulcer Advisory Panel.

**Dr Matthew Regulski, USA**

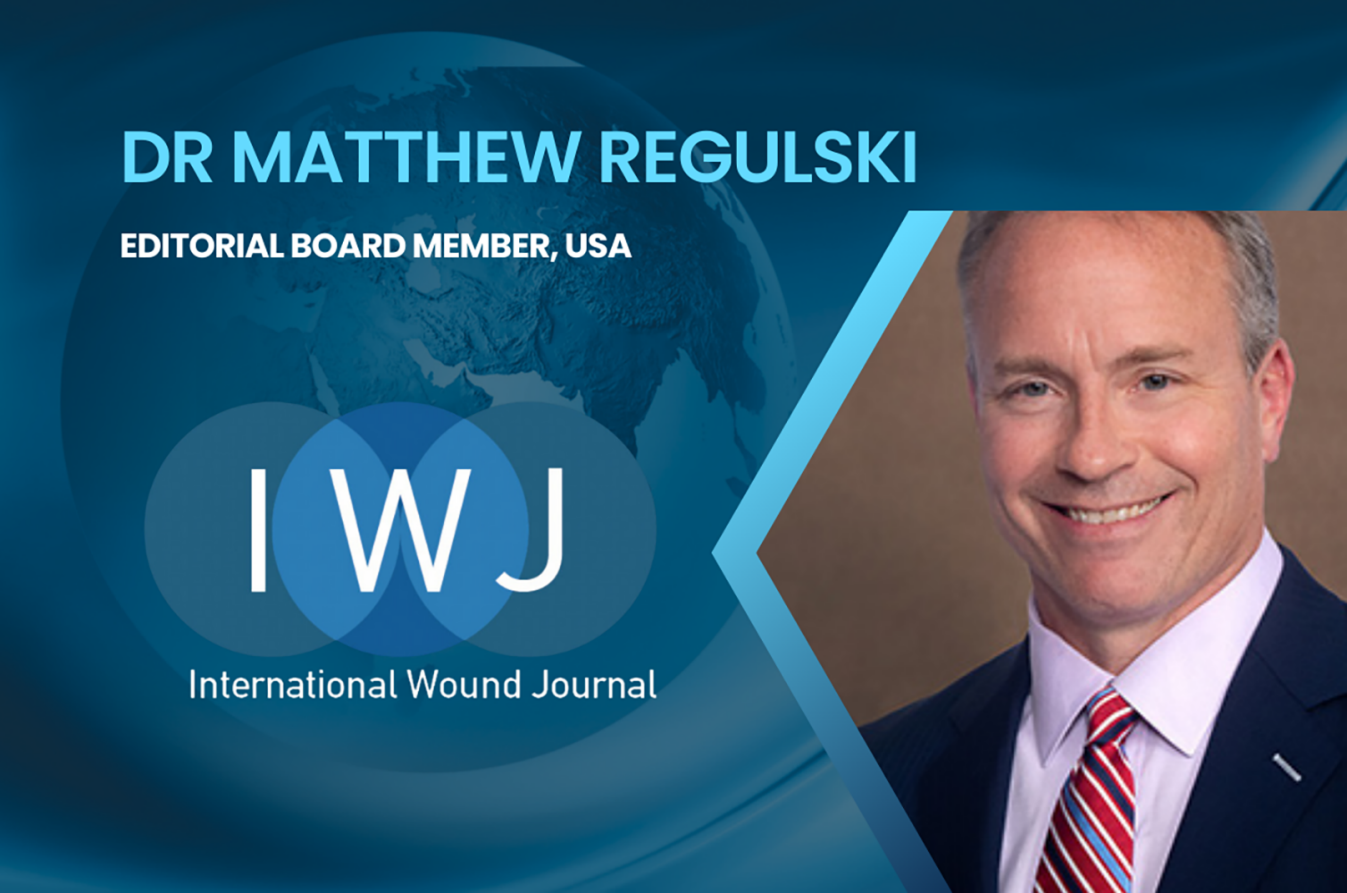

Dr. Regulski is a Graduate of Temple University School of Medicine, where he graduated Summa Cum Laude. He performed his residency at the Main Line Health System in Philadelphia, PA, and is currently the Medical Director of the Wound Care Institute of Ocean County, LLC. Dr. Regulski is also Co‐Director of The Center for Wound Healing & Hyperbaric Medicine at Community Medical Center and is involved at the Wound Care Center at the Kimball Institute. He has been the principal investigator for multiple clinical trials for wound healing, diabetic foot and venous leg ulcers, and has authored several peer‐reviewed articles for the treatment of chronic wound healing and limb‐salvage surgery.

Dr. Regulski is currently on the Board of Directors of the Federation of International Podiatry and is the Scientific Chairman for the Federation of International Podiatry. He is also the Communications Director for the Foot Working Group of the American Diabetes Association. Dr. Regulski is a wound care certified physician and fellow of both the Academy of Physicians in Wound Healing and the American Professional Wound Care Association. An International and National lecturer of wound healing and limb‐salvage surgery, Dr. Regulski has multiple certifications in wound healing, diabetic wounds and limb‐salvage surgery.

Dr. Matthew Regulski is a fellow faculty member at the Royal College of Physicians and Surgeons of Glasgow, Scotland.

**Professor Chandan Sen, USA**

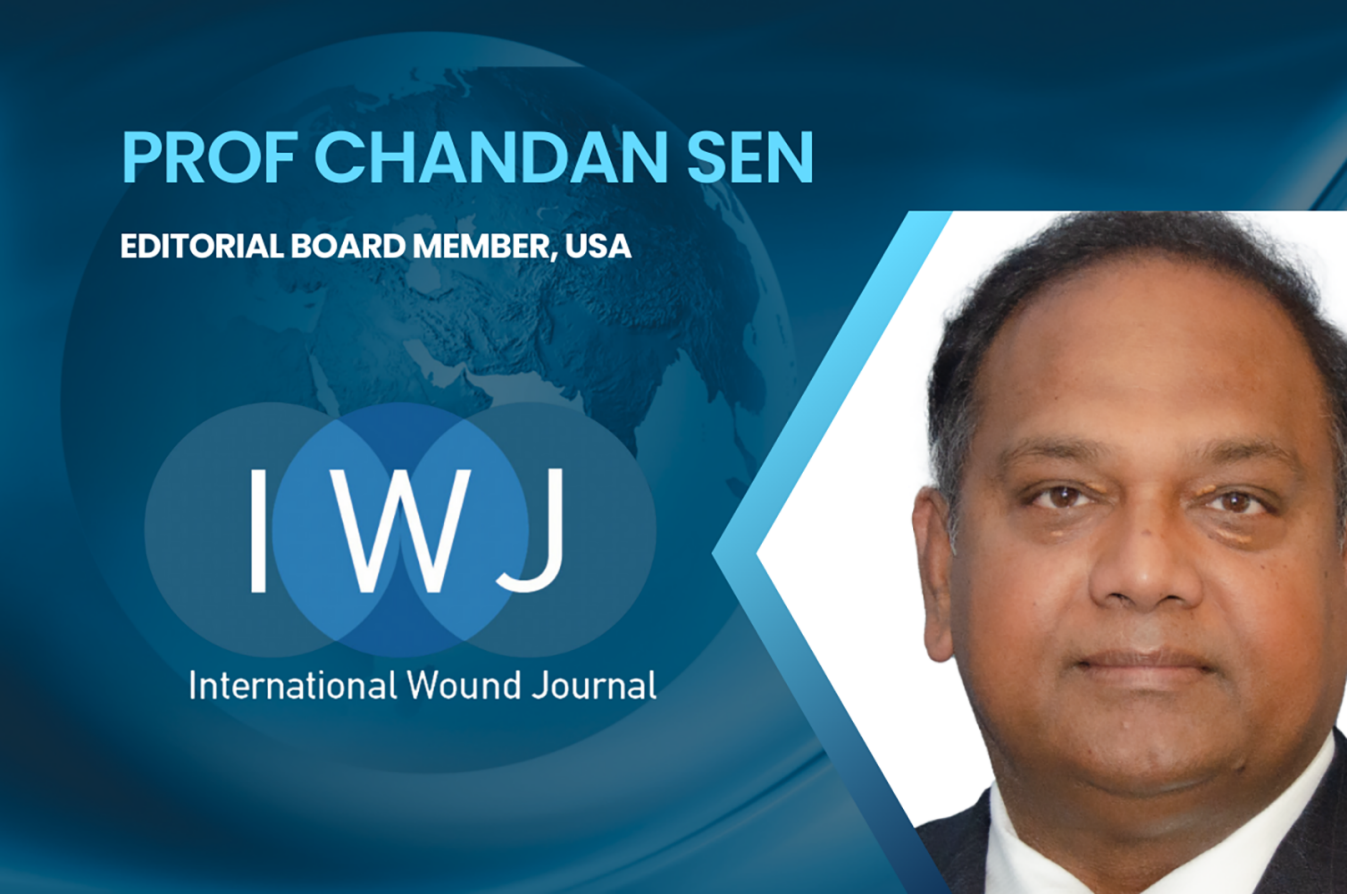

Chandan K. Sen, a world‐renowned regenerative medicine expert and pioneer of novel wound care technologies, joined the University of Pittsburgh in 2023 as associate vice chancellor for life sciences innovation and commercialization, health sciences and also serves as director of the University's McGowan Institute for Regenerative Medicine (MIRM). Prof. Sen is also a professor of surgery in the School of Medicine, with a secondary appointment in the Department of Plastic Surgery, and is the chief scientific officer of UPMC Wound Care Services.

Since 2000, Dr. Sen's research lab has consistently pioneered novel solutions for tissue injury, repair, regeneration and infection and propelled commercialization of a wide variety of therapeutics, preventatives and related products in the field. His scientific breakthroughs in tissue nanotransfection, electroceutical infection management, nanomedicine and bioinformatics apply to a wide range of diseases and conditions. He currently leads a patient‐based biomarker study for the NIH‐funded Diabetic Foot Consortium (DFC) —the first multicenter network to study diabetic foot ulcers. His team is also conducting a DFC study examining whether a breach of barrier function of the skin on a newly healed foot ulcer can predict the likelihood of its recurrence, a finding that would prove transformational in guiding treatment.

Prof. Sen earned his Master of Science degree in human physiology from the University of Calcutta, and his PhD in physiology from the University of Eastern Finland and completed a postdoctoral fellowship in molecular and cell biology at the University of California, Berkeley. Prof. Sen has published more than 350 peer‐reviewed papers (H‐index 110) and a dozen books, which have been cited more than 45,000 times.

**Dr Tom Serena, USA**

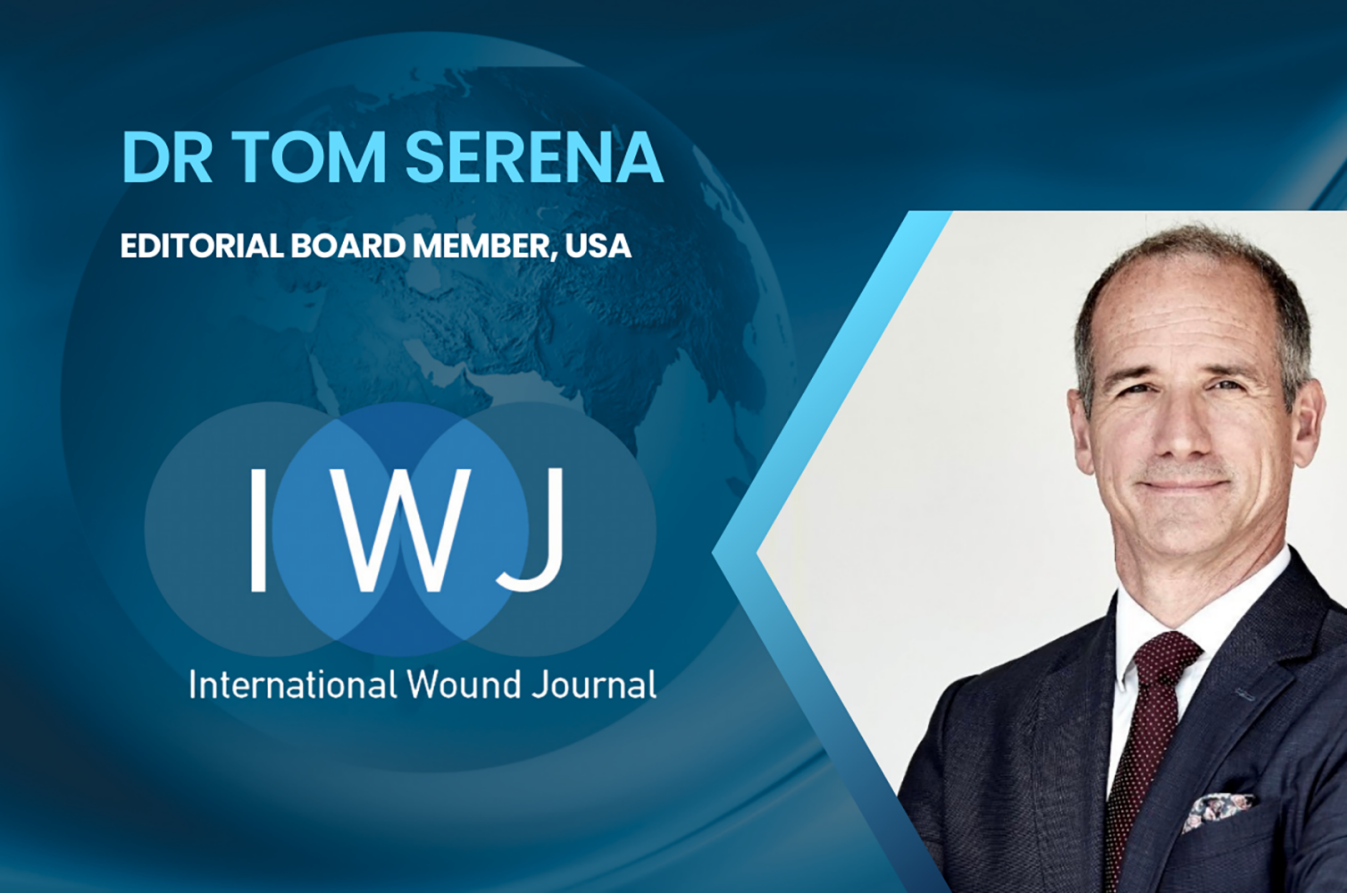

Dr. Thomas Serena, is the Founder and Medical Director of The SerenaGroup®, a contract research organization that conducts clinical trials in a broad range of diseases related to wound healing. SerenaGroup® established a site management organization (SMO) that assists hospitals and clinics in establishing compliant research programmes. In addition, SerenaGroup® provides consulting services for advanced wound and hyperbaric centres across the globe. Dr. Serena completed his residency in Surgery at Penn State's Hershey Medical Center and maintains board certification in Surgery.

Dr. Serena has opened and operates advanced wound care and research centres across the United States and globally. He has been the lead or Principal investigator in over 100 clinical trials, including diagnostics, antimicrobial dressings, growth factors, topical and parenteral antibiotics, and CAMP therapy. In 2011, he developed a diagnostic technique that now bears his name (The Serena Technique©). He is one of the world leaders in clinical research, quality improvement and real‐world data collection. He holds numerous patents on wound care devices and dressings.

Recognized internationally as an expert in the field of wound healing: He has more than 300 published papers and has given more than 2500 invited lectures throughout the world. He has two textbooks coming out in 2024. He has authored numerous book chapters. He has been a member of the Board of Directors of the Wound Healing Society and served two terms on the board of the Association for the Advancement of Wound Care (AAWC) and is a Past‐President. He has also been Vice‐President of the American College of Hyperbaric Medicine and President of the American Professional Wound Care Association. Presently, he is a board member of the FDA‐sponsored Wound Care Collaborative Community (WCCC) and Vice‐President of the International Surgical Wound Complication Advisory Board (ISWCAP).

**Professor Richard Simman, USA**

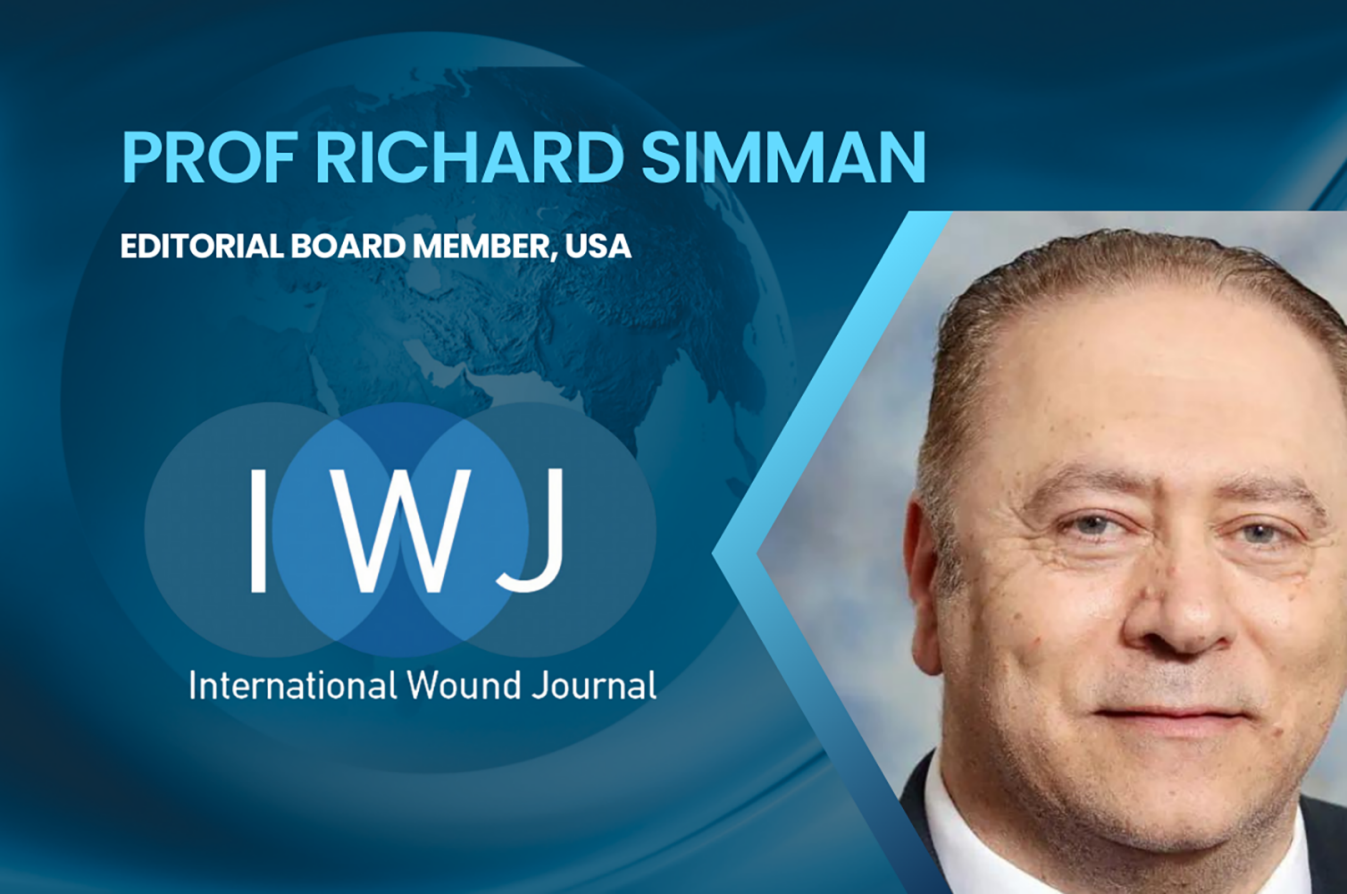

Dr. Simman is the founding president and chair of the American Board of Wound Medicine and Surgery (ABWMS), past president of the American College of Clinical Wound Specialists (ACCWS), past editor‐in‐chief of the journal of the college and sits on the editorial board of the Annals of Plastic Surgery and the Journal of Wound Care (UK).

He is a Clinical Professor at the University of Toledo College of Medicine and Life Sciences, Department of Surgery. He is the Co‐Director of the Wound Care Program at Jobst Vascular Institute/ProMedica Health Network, Toledo, Ohio.

His background includes training in General Surgery at St. Luke's‐Roosevelt Hospital Center, Columbia University in New York, NY and a Plastic Surgery residency at Providence Hospital and Medical Centers in Southfield, Michigan. In addition, he completed a Burn Fellowship followed by a Wound Healing/Cultured Keratinocytes Research Fellowship at the State University of New York at Stony Brook. He has published over 200 peer‐reviewed papers, abstracts, letters and book chapters.

**Professor Ranjani Somayaji, Canada**

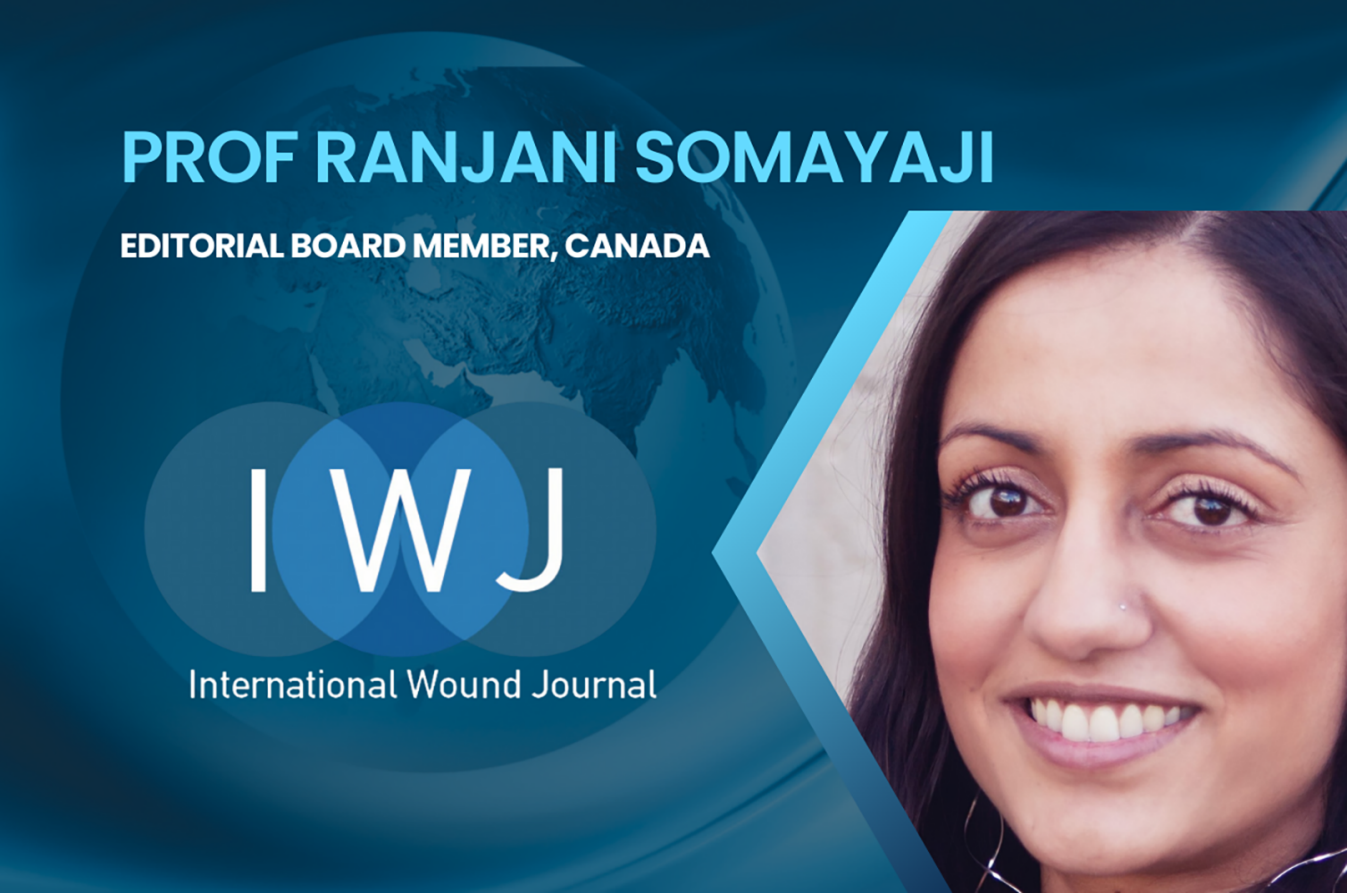

Dr. Ranjani Somayaji completed medical training followed by residency training in internal medicine and fellowship training in infectious disease through the University of Calgary. Dr. Somayaji completed a Master's in Public Health through Johns Hopkins University with a focus on quantitative methodologies. Following this, Dr. Somayaji completed 3 years of post‐doctoral training focused on cystic fibrosis and epidemiological research. Dr. Somayaji is an Assistant Professor as of October 2018 in the Section of Infectious Disease at the University of Calgary and is involved with clinical‐translational research initiatives with national and international collaborations. As an early career investigator and clinician‐scientist, Dr. Somayaji has more than 75 published manuscripts focused on the role and effects of acute and chronic infections on specific populations including persons living with wounds.

Key Areas of Expertise: Epidemiology of infectious disease, population health, microbiology, wound healing.

**Professor Marjana Tomic‐Canic, USA**

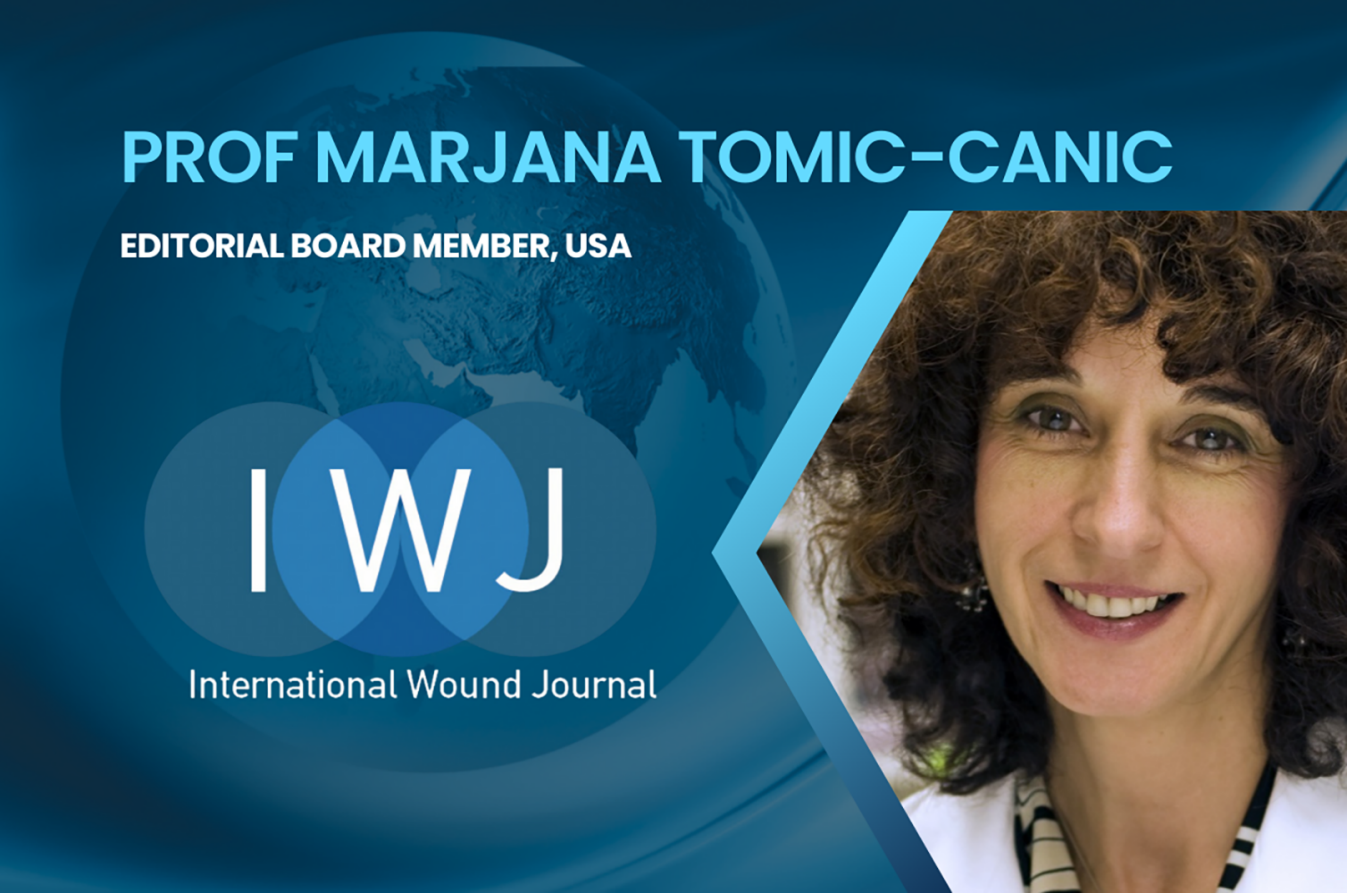

Marjana Tomic‐Canic, Ph.D., is William H. Eaglstein M.D. Chair in Wound Healing, Vice Chair of Research and Professor of Dermatology, and the Director of the Wound Healing and Regenerative Medicine Research Program in the Dr. Phillip Frost Department of Dermatology and Cutaneous Surgery at the University of Miami Miller School of Medicine. She also serves as a professor for the Microbiology and Immunology, Molecular and Cell Pharmacology and Human Genomics and Genetics Graduate Programs. In addition to her full‐time faculty appointment at UM, Tomic‐Canic also holds adjunct faculty appointment at the Ronald O. Perlman Department of Dermatology at the NYU Grossman School of Medicine and NYU Langone Health. Tomic‐Canic received her doctoral and postdoctoral training at NYU School of Medicine before joining the faculty in 1994. She also holds a degree in Nursing, which provides important insights into clinical applicability and translation of her research. In 2005, as a faculty member of the Cornell University Weill Medical College, she directed the Tissue Repair Program at the Hospital for Special Surgery's Department of Tissue Engineering, Regeneration and Repair. Tomic‐Canic joined the Miller School of Medicine in 2008.

For more than 30 years, Tomic‐Canic's primary research interest has been in skin biology and regenerative medicine. Her current research focuses on the molecular and cellular mechanisms of cutaneous wound healing and its pathophysiology, that includes human and diabetic models of wound healing, wound genome, mechanisms of cell‐based therapies, local sustained gene delivery, predictive and diagnostic biomarkers for chronic wounds, innate and adaptive immunity in wound infection and role of ageing in wound healing. A recognized national and international leader and inventor in wound healing research, she has been funded continuously by the National Institutes of Health (NIH) for over 25 years.

Tomic‐Canic has been a member of the Society of Investigative Dermatology since she was a graduate student (membership dates 1990). In addition, she is a member of the Wound Healing Society, serving as its President 2016–2017. She had multiple editorial responsibilities, including serving on the editorial boards of many journals covering dermatology, biological chemistry and wound care. Dr Tomic‐Canic also served at the Advisory Council of the NIH and completed her service as a Chair of the NIH ACTS study section. She serves as a member of the University of Miami Research Council.

**Dr Yi‐Ting Tzen, USA**

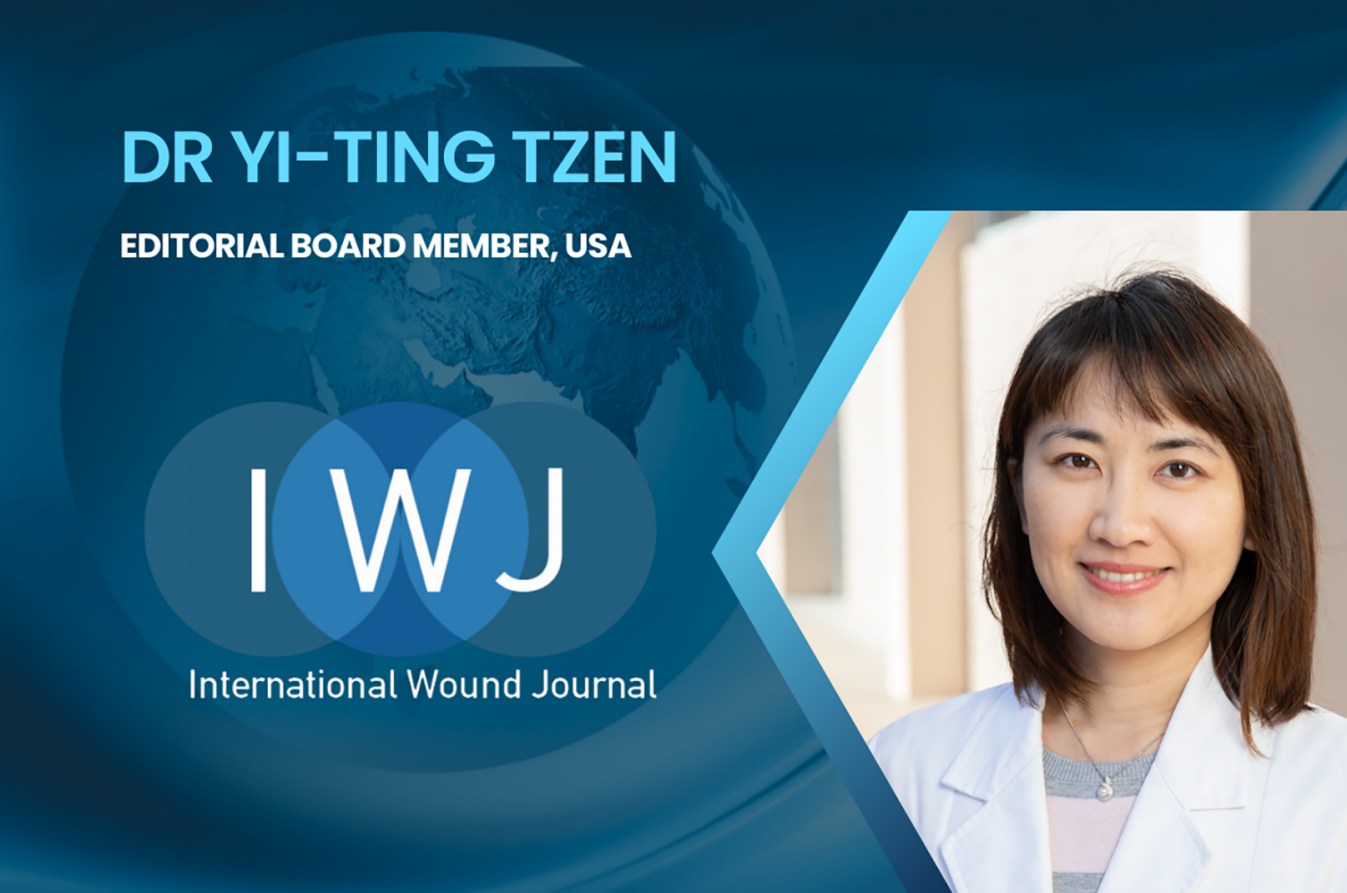

Dr. Tzen's research interest is the early detection of pressure injury in people with spinal cord injury using non‐invasive perfusion markers and serum biomarkers and wound prevention through assistive technology devices. Dr. Tzen had clinical training in Physical Therapy and Assistive Technology, and technology training in bioinstrumentation and signal processing.

**Professor Michael Stacey, Canada**

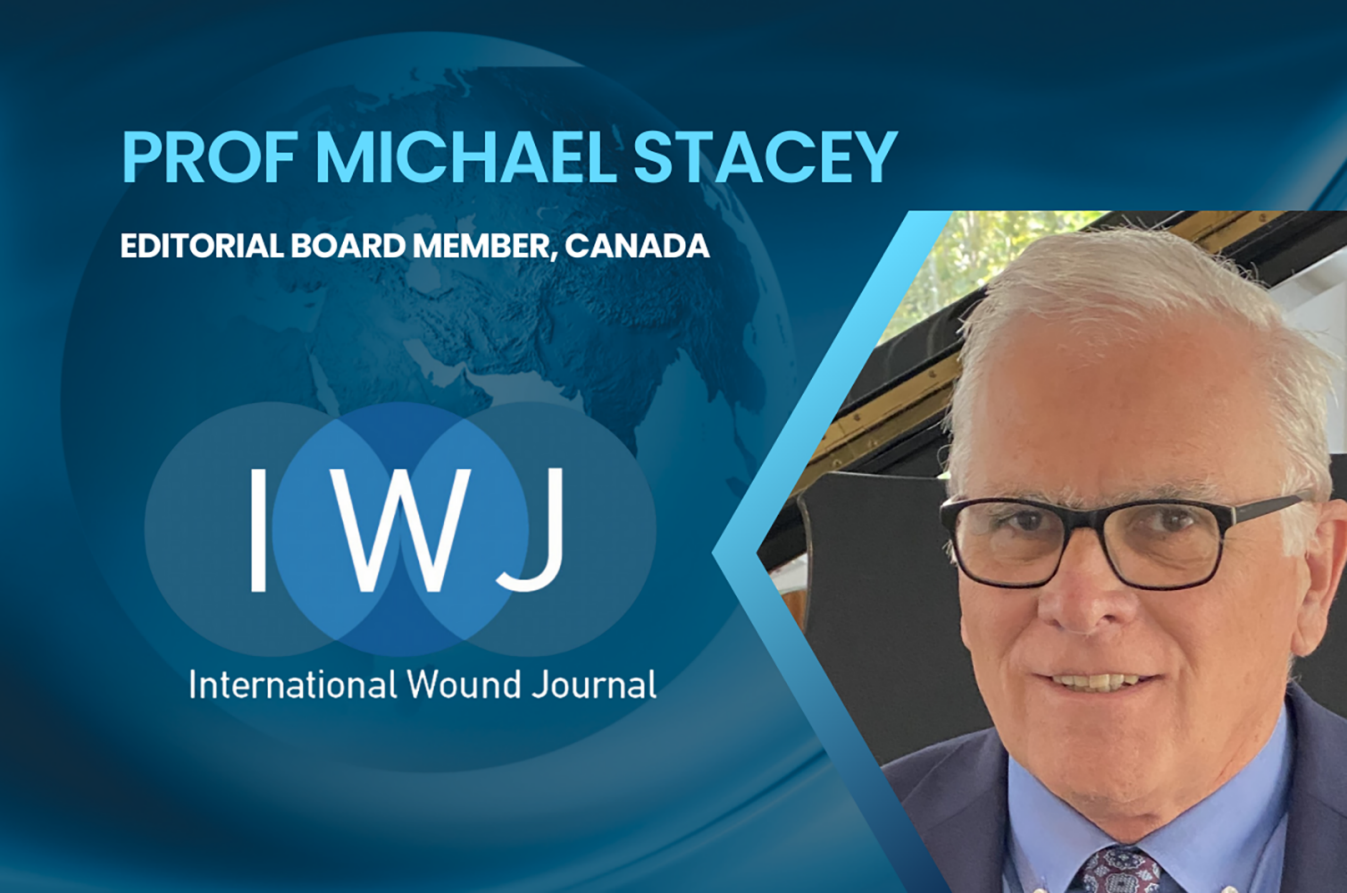

Michael is a vascular surgeon who came to Canada from Australia in 2014 as the Surgeon in Chief at Hamilton Health Sciences and Professor in the Department of Surgery at McMaster University. He completed his medical degree at the University of Western Australia, is a Fellow of the Royal Australasian College of Surgeons, and is licensed with the College of Physicians and Surgeons of Ontario. He spent 2 years working at St Thomas' Hospital in London, United Kingdom, where he conducted research for his Doctor of Surgery degree (PhD equivalent).

Michael took on the role of Chief Medical Executive and Executive Vice President Academic at Hamilton Health Sciences in 2018 until mid‐2023. Currently, he has increased his clinical work in vascular surgery and is a consultant for hospitals and biomedical companies in the areas of clinical practice, research and physician leadership.

As well as his practice in vascular surgery of more than 30 years, Michael has established active research programmes in wound healing in both Australia and Canada. He was the first President of the Australian Wound Management Association (now Wounds Australia), and the founding Chair of the World Union of Wound Healing Societies.

Michael has a passion for advancing wound care for patients. He has previously established a multidisciplinary diabetic foot ulcer clinic and a venous leg ulcer clinic in Australia and is currently establishing similar clinics at Hamilton Health Sciences and in a private clinic setting to provide a service to the Niagara region.

**Professor Joyce Stechmiller, USA**

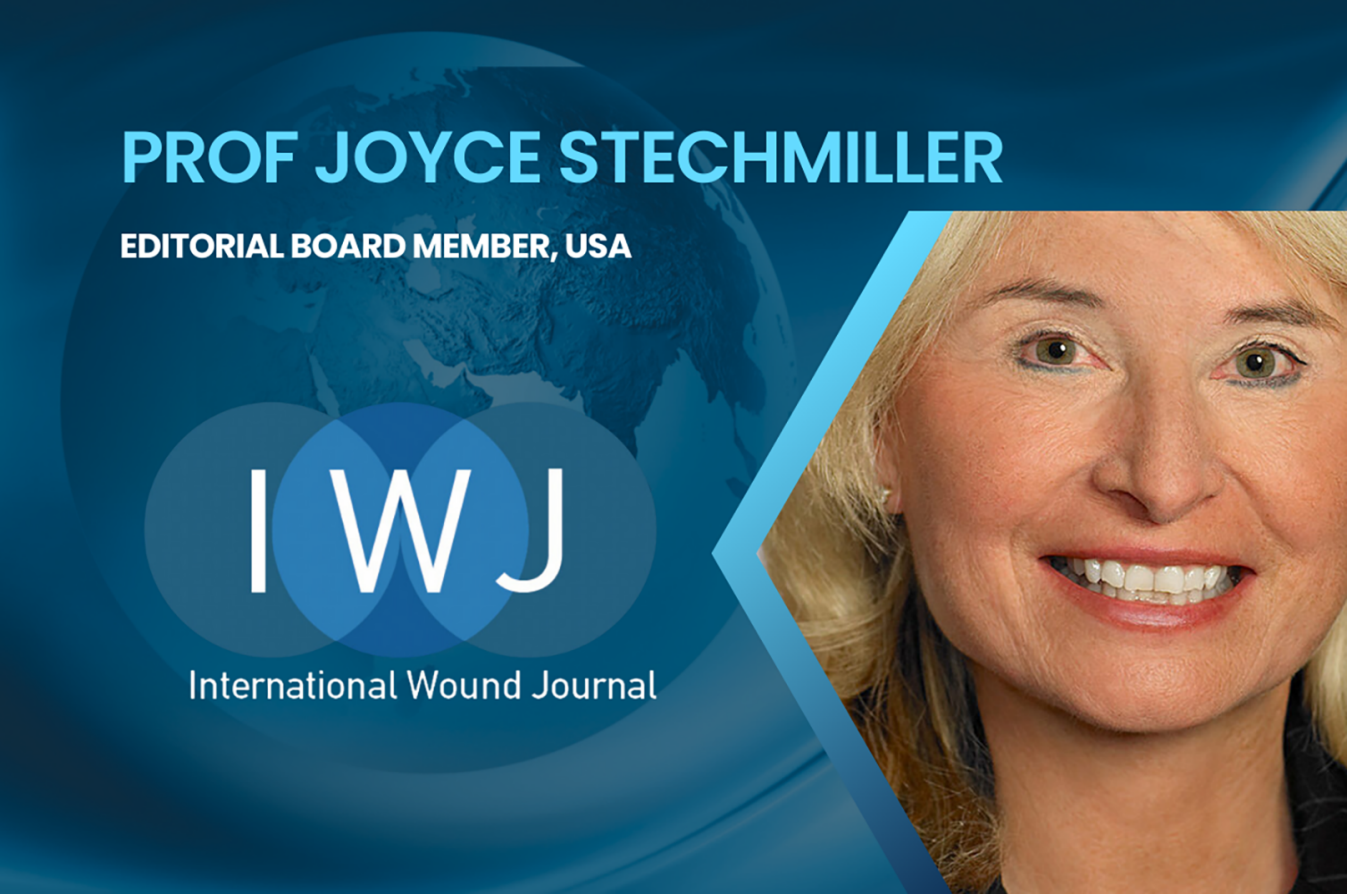

Dr Stechmiller and colleagues are currently studying functional characteristics of biofilm infections in chronic venous leg ulcers and how the presence of biofilm in the wound relates to local and systemic inflammation. We are also measuring symptoms and relating systemic inflammation to symptom clusters in older adults with these wounds. Our central hypothesis is that the interrelated cellular and molecular mechanisms whose immune activation contributes to the development and persistence of CVLUs may also lead to the development and severity of symptoms. It is our hope that biobehavioural research that focuses on the interrelated biobehavioural mechanisms associated with CVLUs will lead to interventions for symptom management that will improve the quality of life for many burdened patients. I have also co‐pioneered the study of how bacterial biofilms disrupt dynamic reciprocity in chronic wounds.

A major focus of my research has been identifying and measuring biomarkers of inflammation of chronic wounds. My work in the area of arginine, immune function and pressure ulcers is original and among the earliest in the field of inquiry regarding immune function, inflammation and nutrition.

Dr Steichmiller and colleagues previously studied inflammation in diabetic foot ulcers, pressure injuries and venous leg ulcers.

Our team was the first to report on pain in nursing home residents with suspected deep tissue injury and pressure ulcers using the newly developed MDS 3.0 data set. For example, among nursing home residents, we found that the pain intensity of persons with pressure ulcers was 15% (Stage II), 22% (Stage III), 44% (Stage IV) and 24% (sDTI) greater than that of persons with a Stage I pressure ulcer after adjusting for other predictors. These findings indicate that pain is a relevant biobehavioural concept in individuals with chronic wounds.

We also examined which epidemiological factors are associated with suspected deep tissue injury in nursing home residents with an analysis of the national MDS 3.0 dataset.

In collaboration with national and international colleagues directed the development and dissemination of evidence‐based guidelines for pressure ulcer prevention and management.

**Professor John Steinberg, USA**

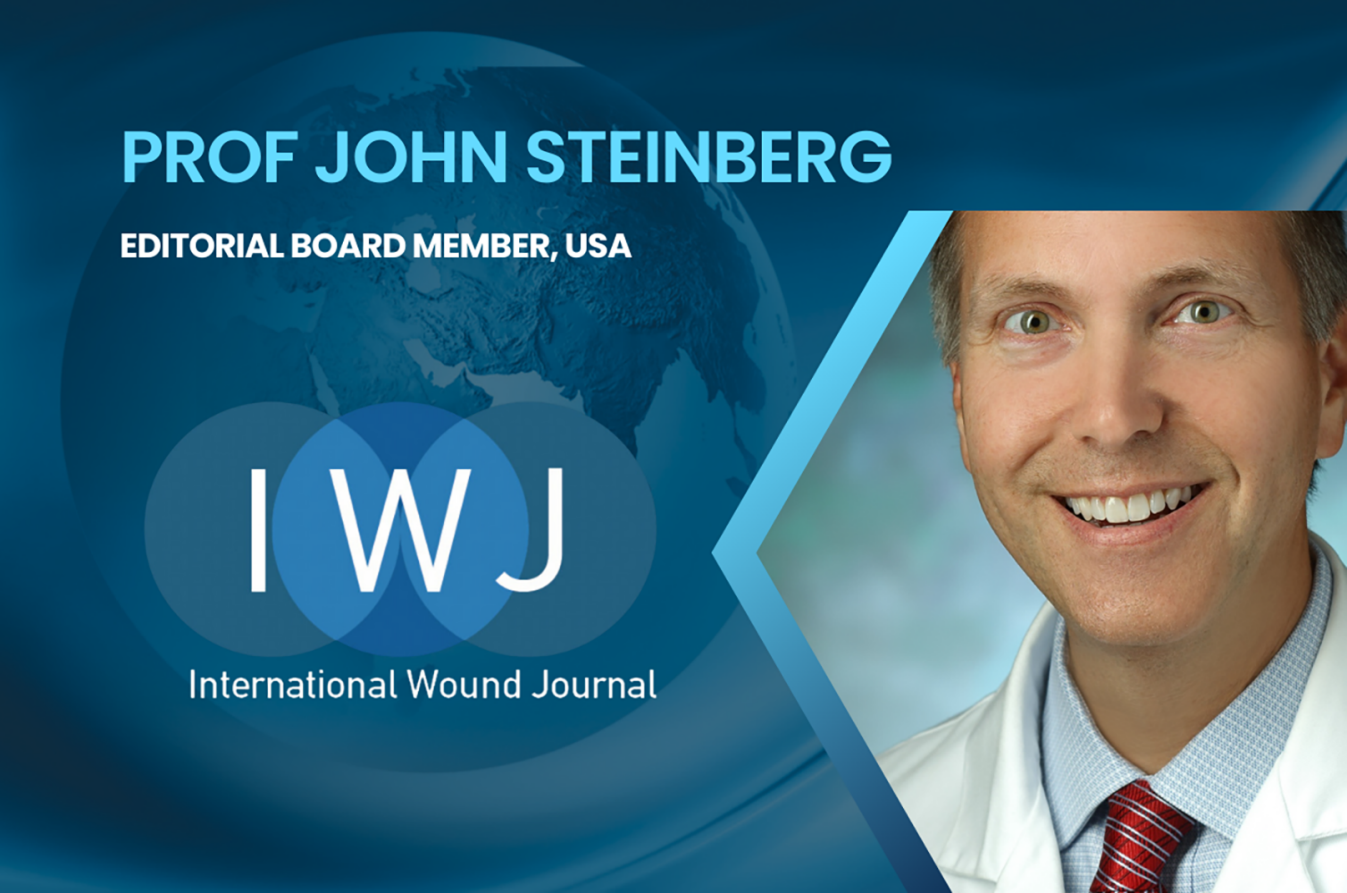

John S. Steinberg, DPM, FACFAS, is the Co‐Director of the Center for Wound Healing at MedStar Georgetown University Hospital and is the Director of the Podiatric Residency Training Program at MedStar Health. Dr. Steinberg is a Professor in the Department of Plastic Surgery at Georgetown University School of Medicine. He is board‐certified by the American Board of Podiatric Surgery in foot surgery and in reconstructive rearfoot and ankle surgery. Dr. Steinberg completed a 3‐year surgical residency at the Inova Fairfax Podiatric Residency Program and then completed a 1‐year Diabetic Foot/Limb Salvage Fellowship with the University of Texas at San Antonio.

In addition to general foot and ankle care, the majority of Dr. Steinberg's practice is devoted to non‐healing wounds and surgical care of patient with diabetic foot complications. His wound care team takes a progressive, often surgically based approach that helps to speed healing and avoid amputation.

Dr. Steinberg has lectured extensively on diabetic limb salvage both nationally and internationally. He dedicates significant effort to clinical research, education and publication in the field of wound healing and limb salvage surgery.

Dr. Steinberg comes from a family of podiatrists. He takes great pride in his specialty and his unique contributions to the field. Dr. Steinberg works with a multi‐disciplinary team that includes plastic surgeons, vascular surgeons and infectious disease specialists. Dr. Steinberg stresses the importance of including the patient's family in decisions made and communicating thoroughly with all who are involved.

**Dr Thomas Stewart, USA**

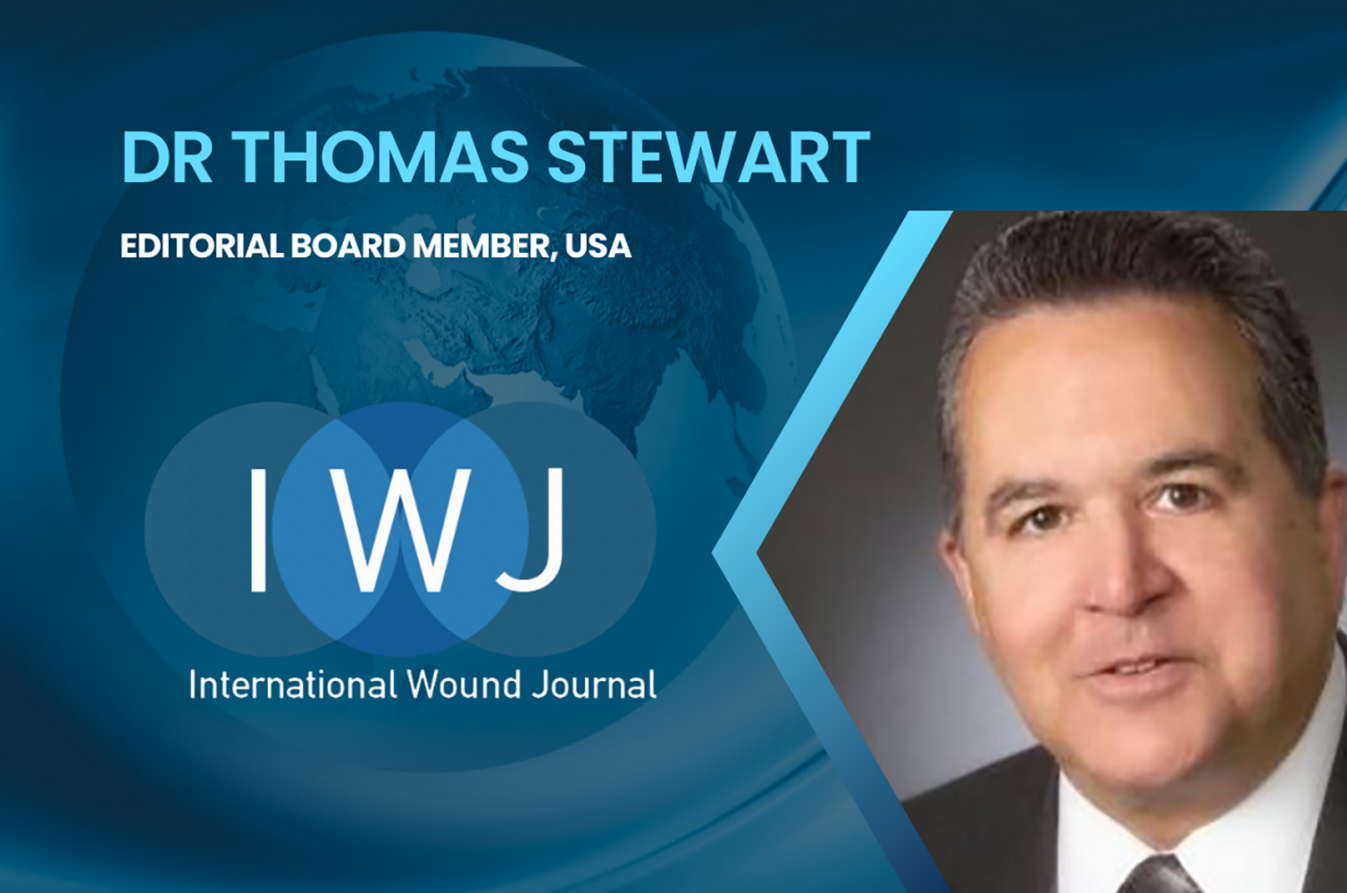

Dr. Thomas P. Stewart was the VP for Clinical Affairs at the Stryker Corporation's Medical Division for approximately 2 years after the sale of Gaymar Industries to Stryker in 2010. He was the President & CCO of Gaymar Industries from 2000 to 2010. Gaymar was a Global Medical Device Company, joining in 81 to oversee its Clinical Research; promoted to Dir Med Res, VP S & Mkt, VP Ops, VP NPD, Pres & COO.

Undergraduate degree: Daemen University, a doctorate from SUNY/ Buffalo, is an alumnus of its School of Med/Applied Sciences. In 1987, he founded the National Pressure Injury Advisory Panel; in 2003, he received the Panel's first ‘Thomas Stewart Award’, given annually to individuals who demonstrate leadership in public policy related to pressure injury care and patient advocacy.

He is a Senior Associate Clinical Professor at Daemen University and an adjunct Asst. Prof. at SUNY/Buffalo has authored over 30 articles in peer‐reviewed journals and a book chapter. Over 25 years of expertise in pressure ulcer pathophysiology, wound healing, support surface design and measurement, thermal physiology and nosocomial lesions acquired by patients during operating room and hospital stays; expert witness in medical malpractice cases nationwide—co‐inventor on seven patents for the therapeutic control of patient temperature and compression therapy.

**Dr Arthur Stone, USA**

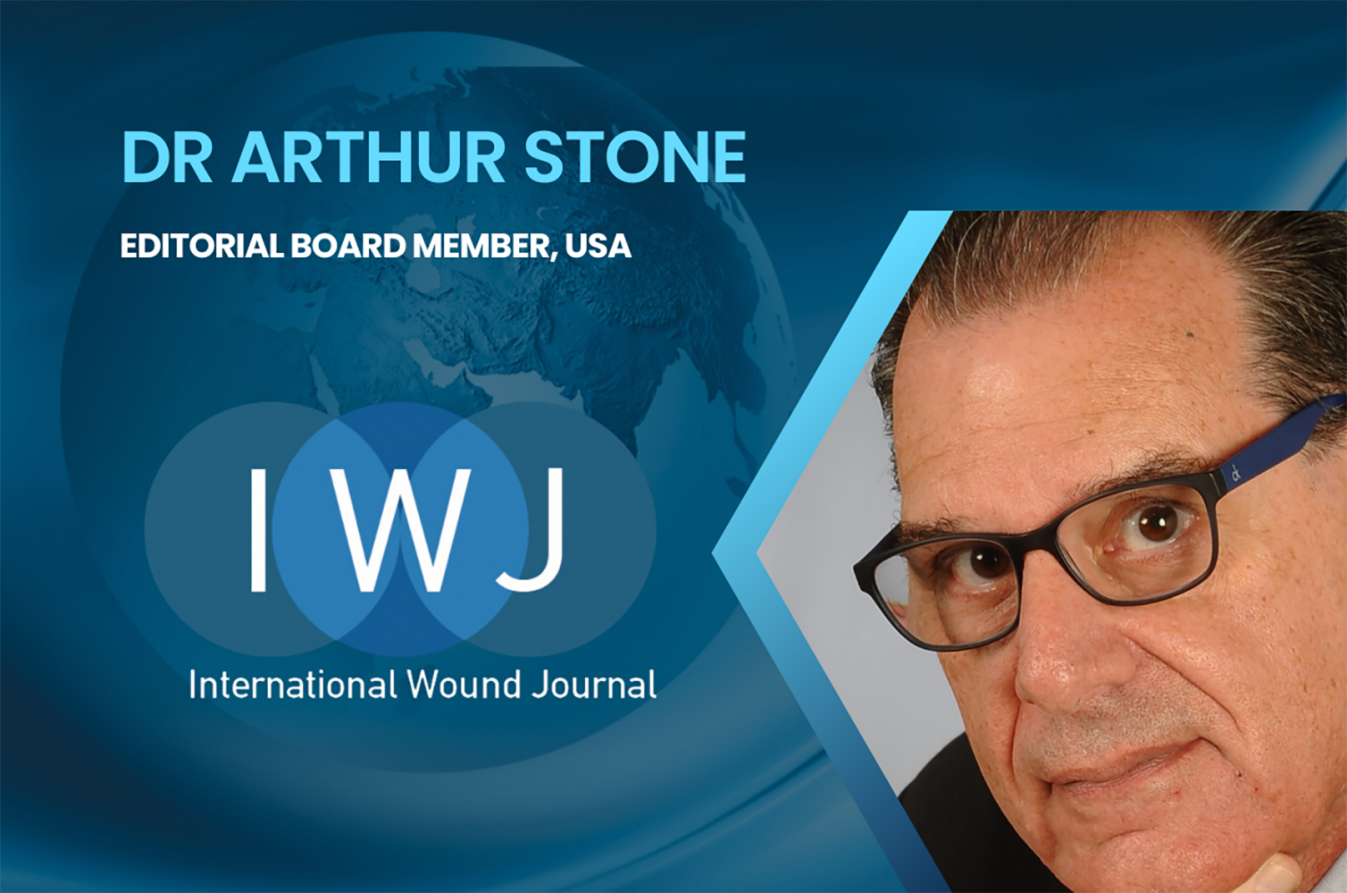

As a practicing podiatrist Dr Stone has provided care in Long‐Term Care facilities in South Carolina for the past 30 years. Through Dr. Stone's company, MedNexus, Inc, he has provided consulting services to ConvaTec, Hill‐Rom and Smith & Nephew and Stryker to name a few. He is a past Board member of The National Pressure Ulcer Advisory Panel/Support Surface Standards Initiative (S3I) and MAP member of Post‐ Acute/Long‐Term Care committee of the National Quality Forum/CMS. He is also an Advisory Board member for Advances in Skin and Wound Care.

Dr. Stone has been an invited speaker at wound conferences both nationally and internationally. He has chaired and participated in clinical trials/advisory panels and focus groups. He is a member of multiple wound care organizations and has published numerous articles.

**Dr Shabnam Vaezzadeh, Canada**

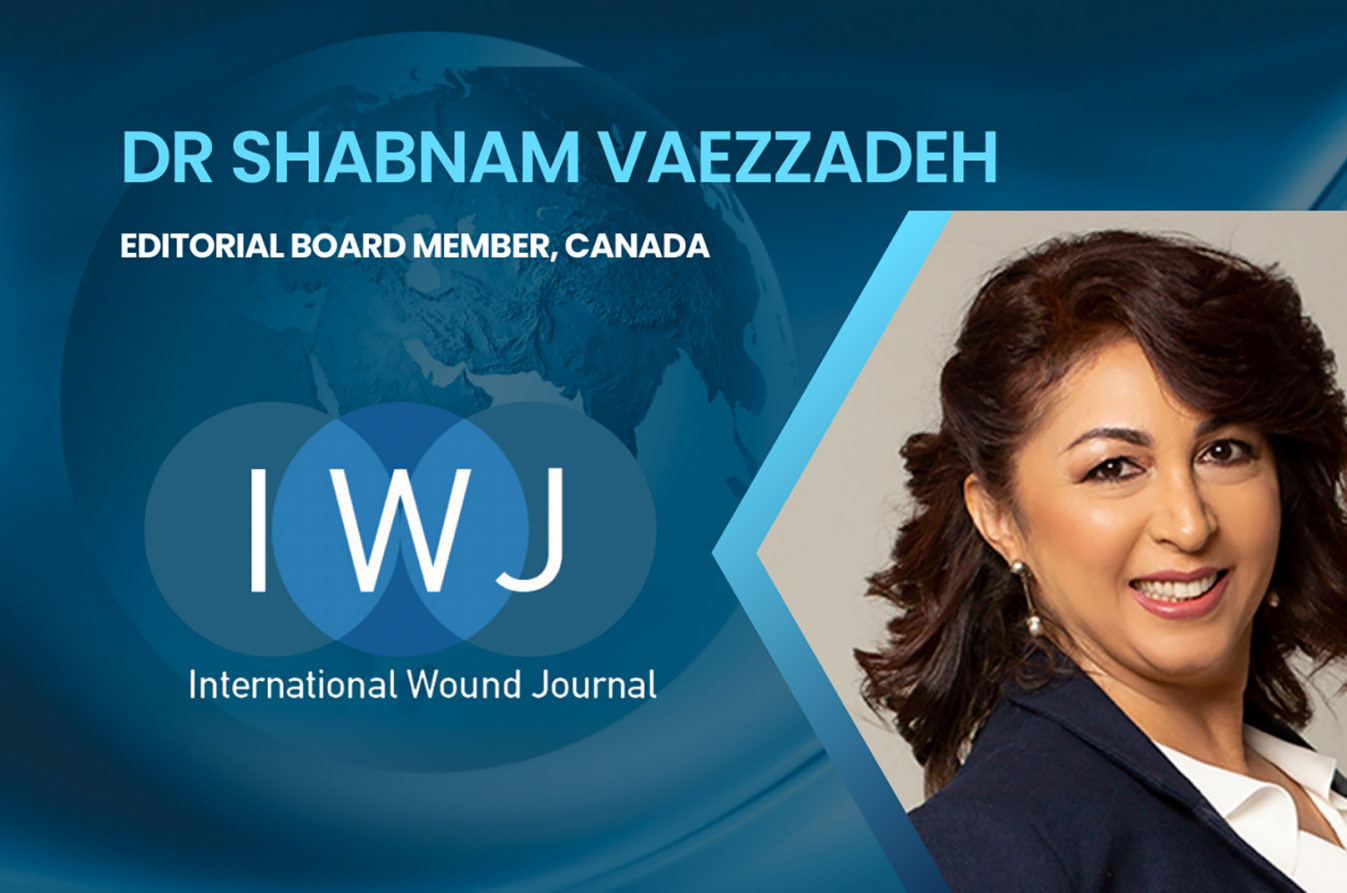

Dr. Shabnam Vaezzadeh is a physician with over 22 years of global corporate leadership in Product Safety, Medical Affairs, Clinical Development, including due diligence and business development, in prominent Medical Device and Regenerative Medicine companies, such as LifeScan (Johnson and Johnson), KCI/Acelity and Organogenesis. As the CEO of Exquisite Biomedical Consulting (EBC), Dr. Vaezzadeh is a life sciences industry consultant for 7 years.

Shabnam's background spans technologies and therapeutic areas including diabetes, wound healing, regenerative medicine, abdominal wall repair, breast reconstruction, orthopaedics and sports medicine, general surgery, neuromodulation and cardiology. Shabnam has led impactful initiatives in collaboration with expert physicians and clinicians, resulting in safe and effective market adoption of novel medical products. She has been responsible for generation and communication of clinical and economic evidence for various advanced medical technologies, notably in wound healing and tissue regeneration. She has guided several important wound care publications, some published by IWJ, and incorporated published evidence in global medical education. EBC advises startups on enhancing their value proposition and supports investors and accelerator on their ventures.

Dr. Vaezzadeh is a contributing member of the Wound Care Collaborative Community, an advisor to Silicon Valley Advantage and Innovate Calgary, a mentor with CELS SFO, and a member of Women in Business Leadership.

**Professor Diane Vilar‐Compte, Mexico**

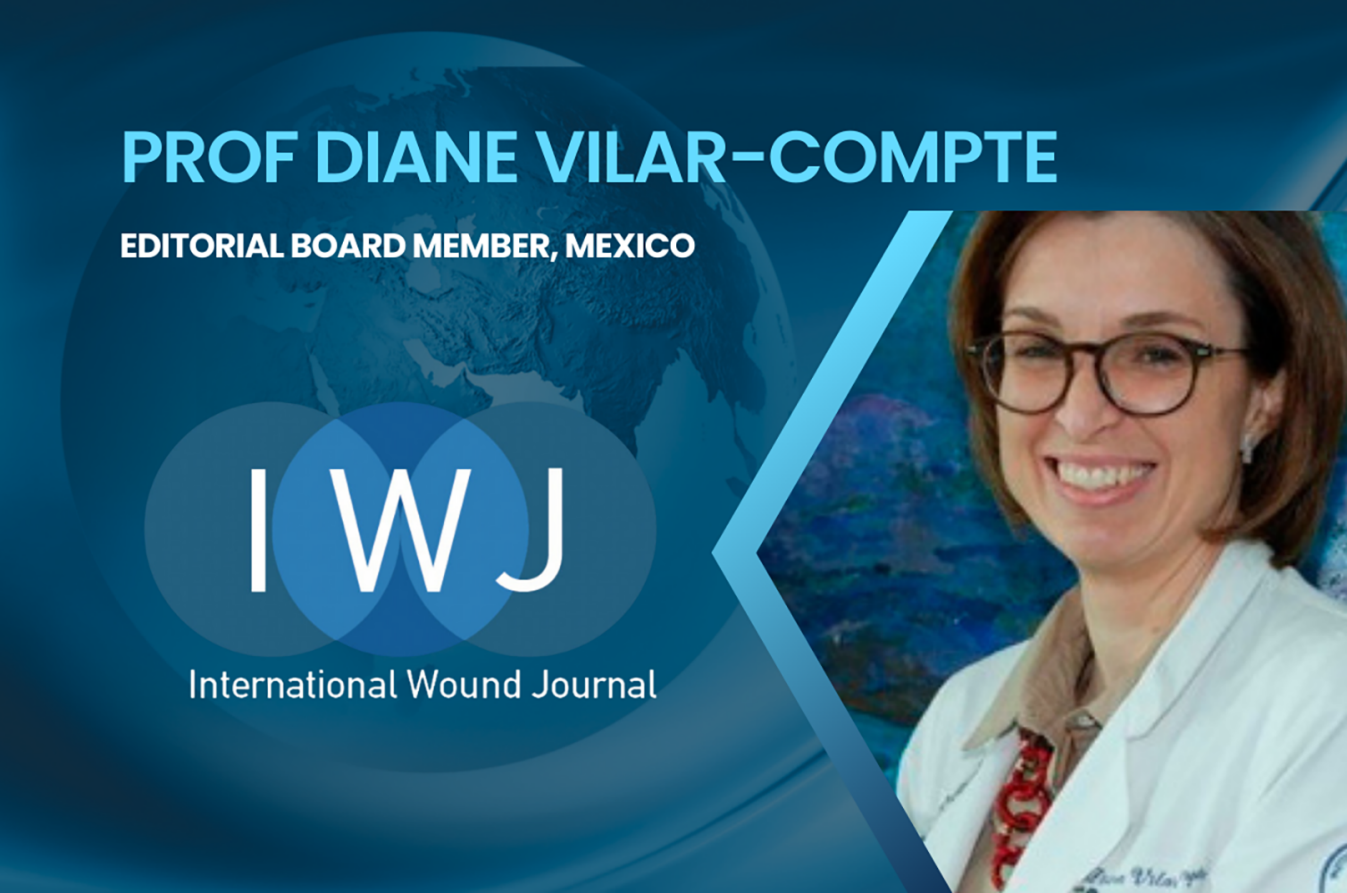

Professor Vilar‐Compte studied surgery at the National Autonomous University of Mexico. She also received a Master in Epidemiology from the same university. This was followed by specialization in Quality Management in Health Services, from the University of Murcia, Spain.

Diane is an affiliated Physician in the Department of Infectology, National Cancer Institute and Professor at the Faculty of Medicine of the UNAM. She is a Member of the National System of Researchers since 2005, currently, Level II and Researcher affiliated with CISIDAT.

Professor Vilar‐Compte is a Member of the National Academy of Medicine, the Mexican Association of Infectology and Clinical Microbiology, the Mexican Association for the Study of Nosocomial Infections, the American Society for Microbiology, the Society for Hospital Epidemiology of America, and the Mexican Association for the Care and Healing of Wounds.

Professor Vilar‐Compte's research interests include surgical complications, particularly surgical site infections and issues related to influenza and respiratory viruses. They also include chronic wounds and topics related to Hospital Epidemiology, infection control, antiseptics and quality of life.

**Dr Nicola Waters, Canada**

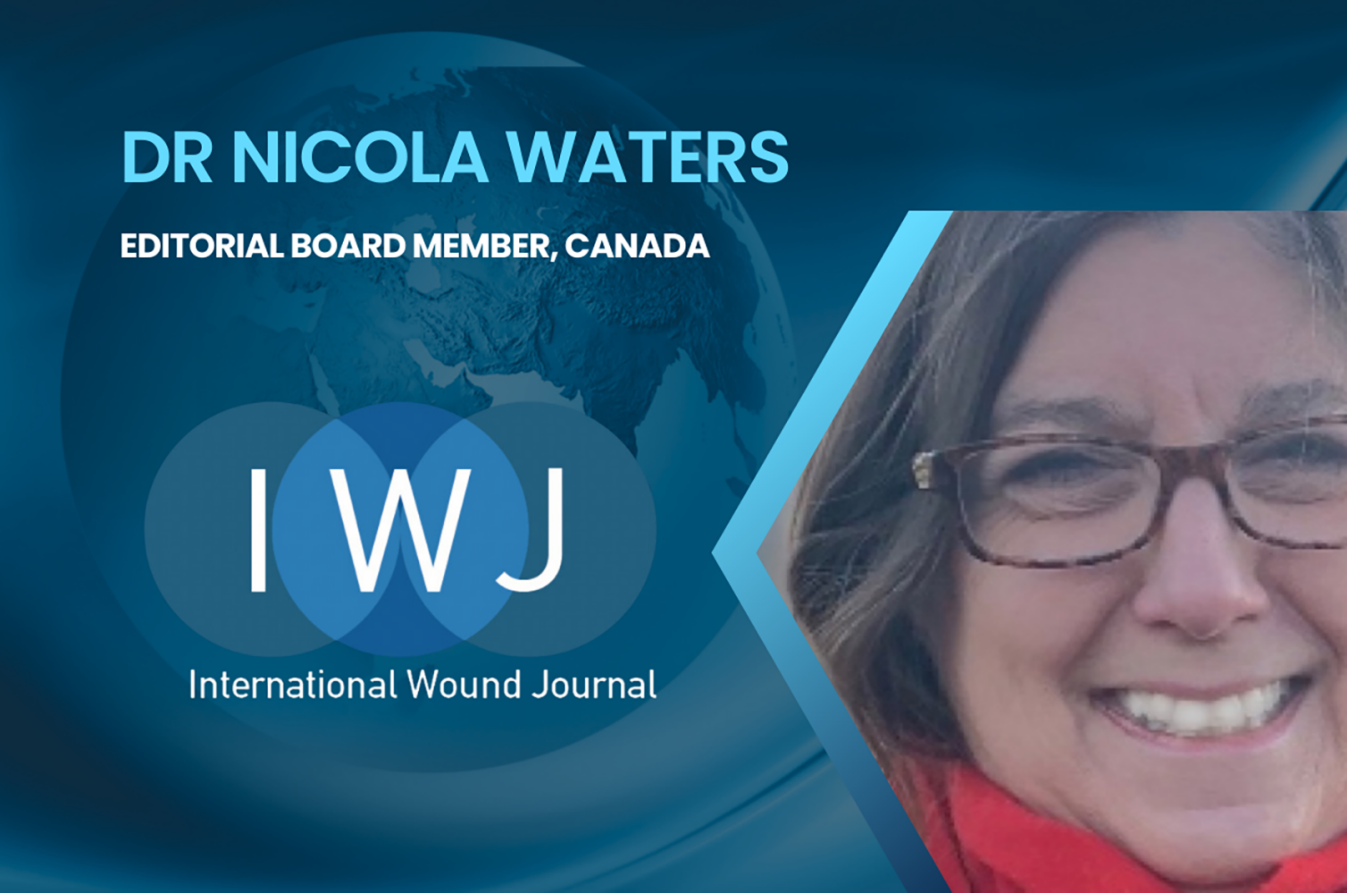

Researcher at the interface between humanity and technology. My experience in clinical practice, social and technical sciences, industry and academia offers a unique opportunity to foster translational links that honour differences while meeting common goals.

Dr Waters research interests include: Clinical Infectious Disease (Lead Health Research Cluster); Wound Healing and Tissue Repair Disaster Preparedness (Focus on sexual assault during disaster events); Institutional Ethnography‐ The Social Organization of Health Work Patients' Experience of Living with Chronic Wounds; Skin and Wound Management (Including remote delivery of wound care); Pressure Ulcer Prevention and Management and Diabetic Foot Ulcer Prevention and Management.

**Professor Stephanie Woelfel, USA**

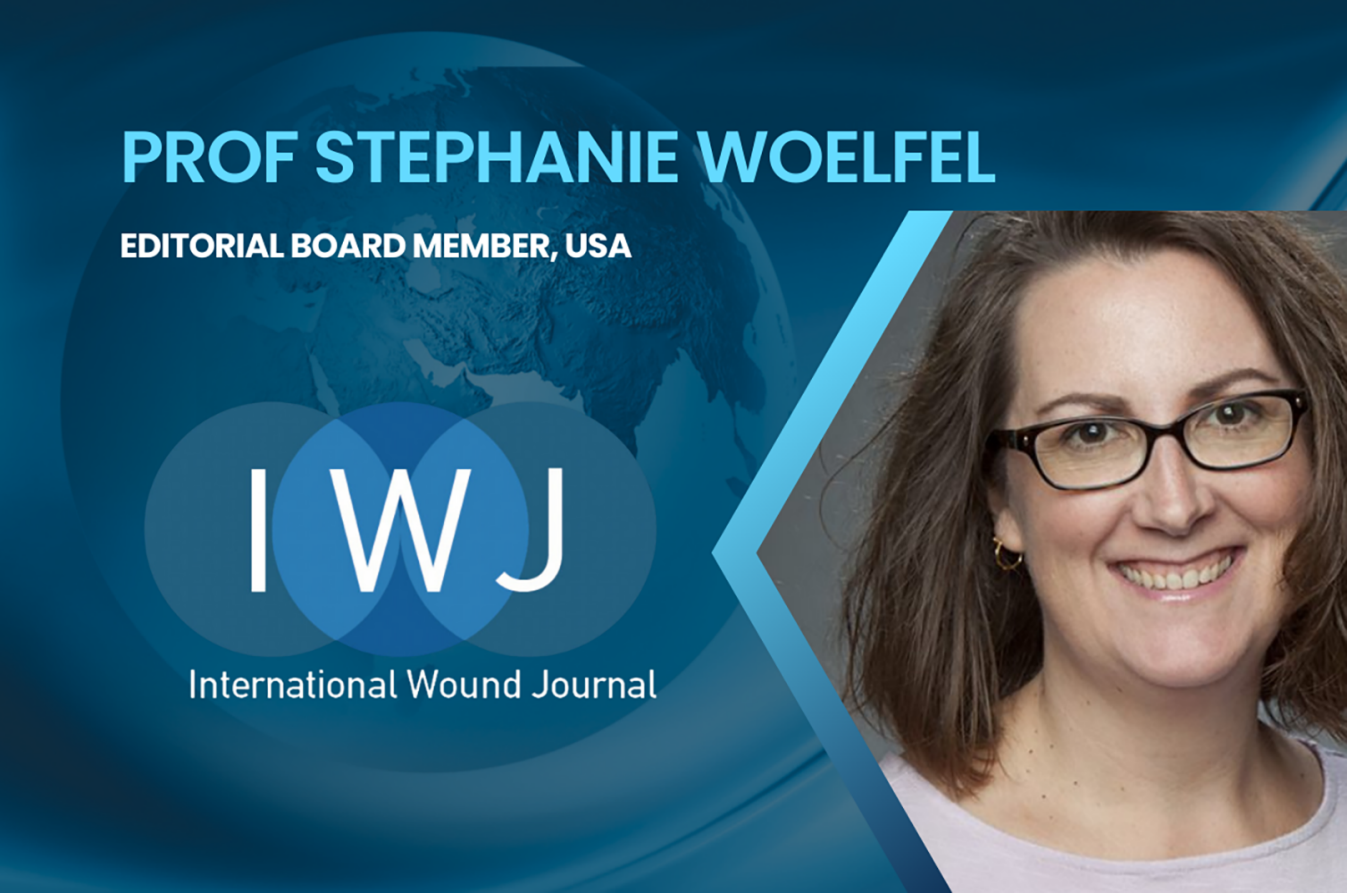

Dr. Stephanie Woelfel is an associate professor of clinical physical therapy with a dual faculty appointment in the Department of Surgery at USC. She is also the director of clinical physical therapy for hospital outpatient services at Keck Medical Center of USC and serves as the primary physical therapist in the Southwestern Academic Limb Salvage Alliance (SALSA) clinic of USC.

Her wound care career has spanned over 20 years in both short and long‐term acute care, skilled nursing facilities, outpatient, home care and consulting. Dr. Woelfel is active in the American Physical Therapy Association (APTA) and serves as the current president of the Academy of Clinical Electrophysiology & Wound Management (ACEWM). She also serves on the Board of Directors of the National Pressure Injury Advisory Panel (NPIAP).

**Dr Tom Wolvos, USA**

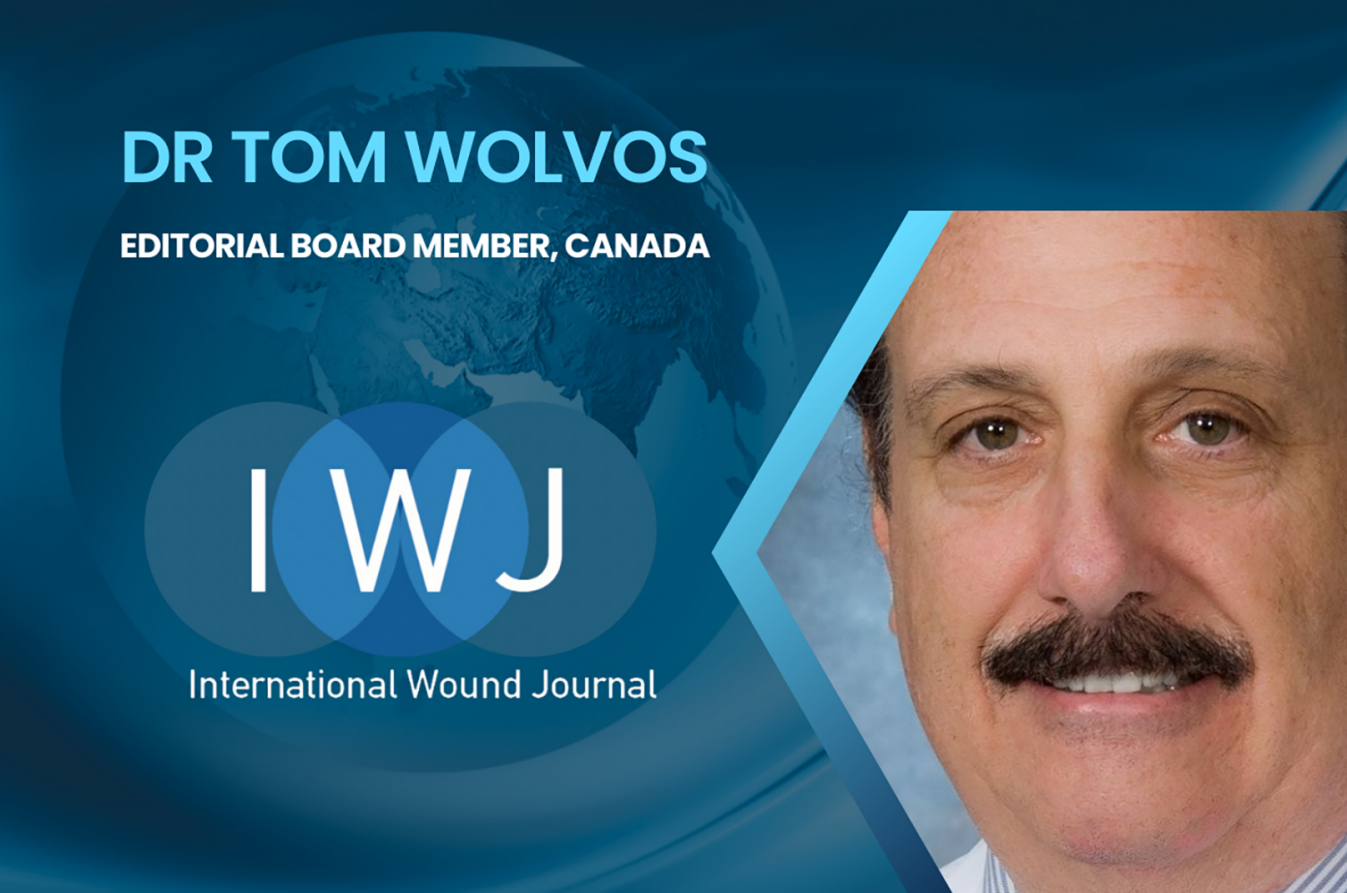

Dr. Wolvos completed his general surgery residency from St. Elizabeth's Hospital of Boston, Massachusetts. He has been in practice for over 40 years having performed general and peripheral vascular surgery, advanced wound care and hyperbaric medicine. He has numerous peer‐reviewed publications in wound journals and textbooks. He published the first two articles on the Wound VAC Instill. He has given over 200 presentations in Advanced Wound Care in seven different countries. He is the editor of the Scottsdale Wound Management Guide now in its third edition. He presently resides in Scottsdale Arizona where he is the HonorHealth Network Medical Director of Wound Management and Hyperbaric Services, Scottsdale/Phoenix Arizona, USA.

**Professor Kevin Woo, Canada**

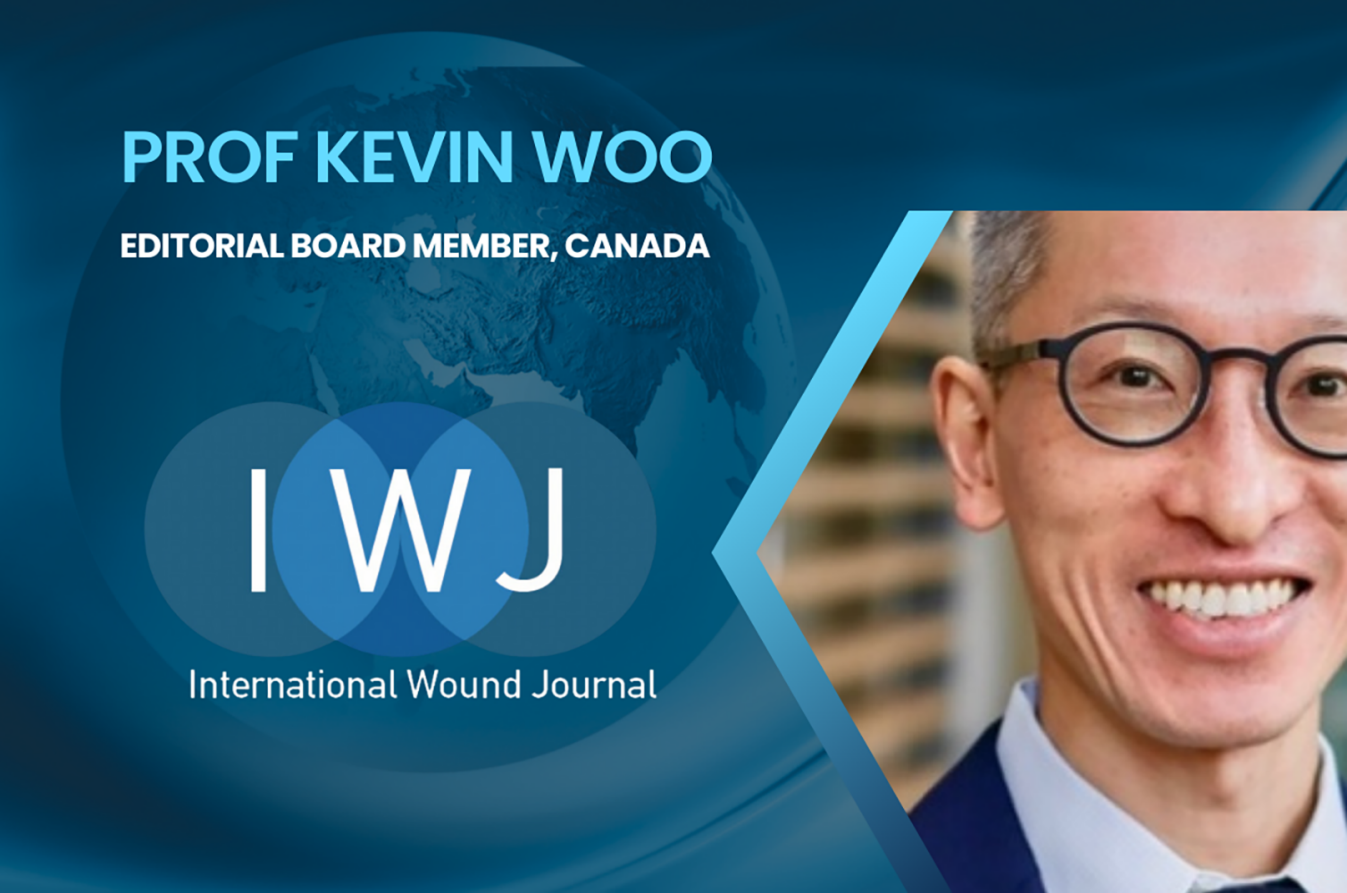

Dr. Kevin Woo is a Full Professor at Queen's University, Faculty of Health Sciences in Kingston, Ontario. He is an adjunct research professor at Western University. He is an affiliate Scientist at the Institute for Education Research (TIER), University Health Network.

He is known as an established Canadian researcher and educator in the fields of wound management and chronic disease management. He has published over 180 peer‐reviewed papers and received national and international awards for his work. Dr. Woo serves as the Chair of the Joint Research Ethics Board for West Park Health Center and Toronto Grace Health Center in Toronto. He is a founding member and the past president of the Canadian Pressure Injury Advisory Panel. He maintains an active clinical role in wound care.

**Professor Dane Wukich, USA**

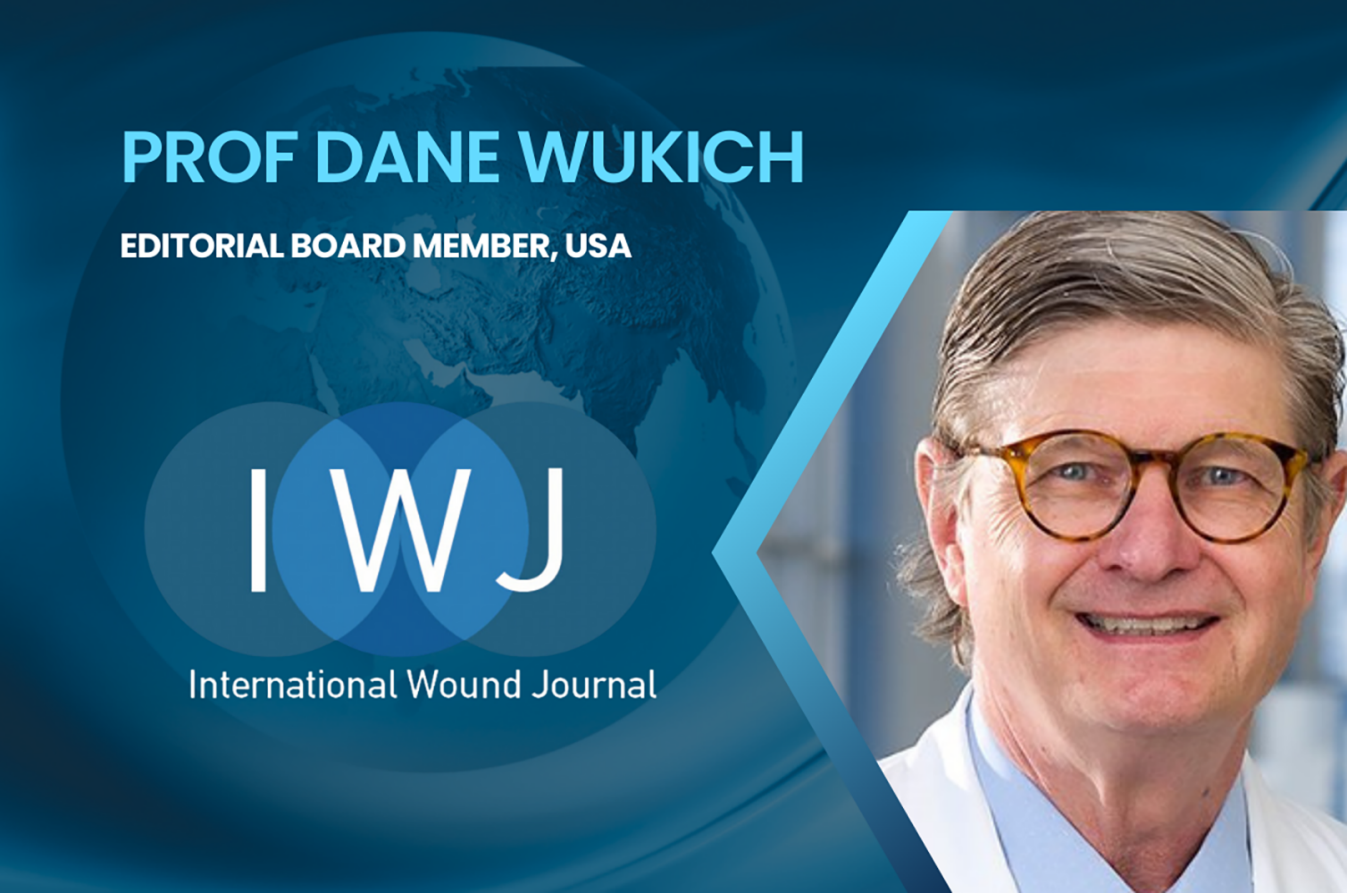

Dane Wukich, is Professor and Chair of the Department of Orthopaedic Surgery at UT Southwestern, where he holds the Dr. Charles F. Gregory Chair in Orthopaedic Surgery. He is also the Medical Director of Orthopaedic Surgery at UT Southwestern University Hospitals.

Dr. Wukich received his undergraduate education from Carnegie‐Mellon University and earned his medical degree at Georgetown University School of Medicine. He completed his general surgery residency and orthopaedic surgery residency at Walter Reed Army Medical Center, followed by a fellowship in foot and ankle surgery at the Cleveland Clinic Foundation.

As an Army Medical Corps officer, Dr. Wukich served as a chief of orthopaedic surgery in Operations Desert Storm and Desert Shield. He achieved the rank of Major with the U.S. Army, receiving several Army Commendation Medals.

Dr. Wukich is a nationally renowned foot and ankle specialist, educator, lecturer and researcher. He has written more than 100 papers and given invited lectures around the globe. He serves as peer reviewer for 11 journals, and is the author or co‐author of several book chapters dealing with foot and ankle problems in athletes and patients with diabetes. His research interest includes the complications of circular external fixation in patients with diabetes, foot and ankle problems in post‐transplant patients, and treatment of spastic deformities of the foot and ankle in patients with traumatic brain injury and/or stroke.

He remains actively involved in the education of orthopaedic surgery not only around the world but also here at UT Southwestern with orthopaedic residents.

Dr. Wukich is board certified by the American Board of Orthopaedic Surgery, and is a member of numerous professional organizations, including the American Academy of Orthopaedic Surgeons, the American Orthopaedic Foot and Ankle Society, the American Orthopaedic Association and the American Diabetes Association.

**Professor Min Zhao, USA**

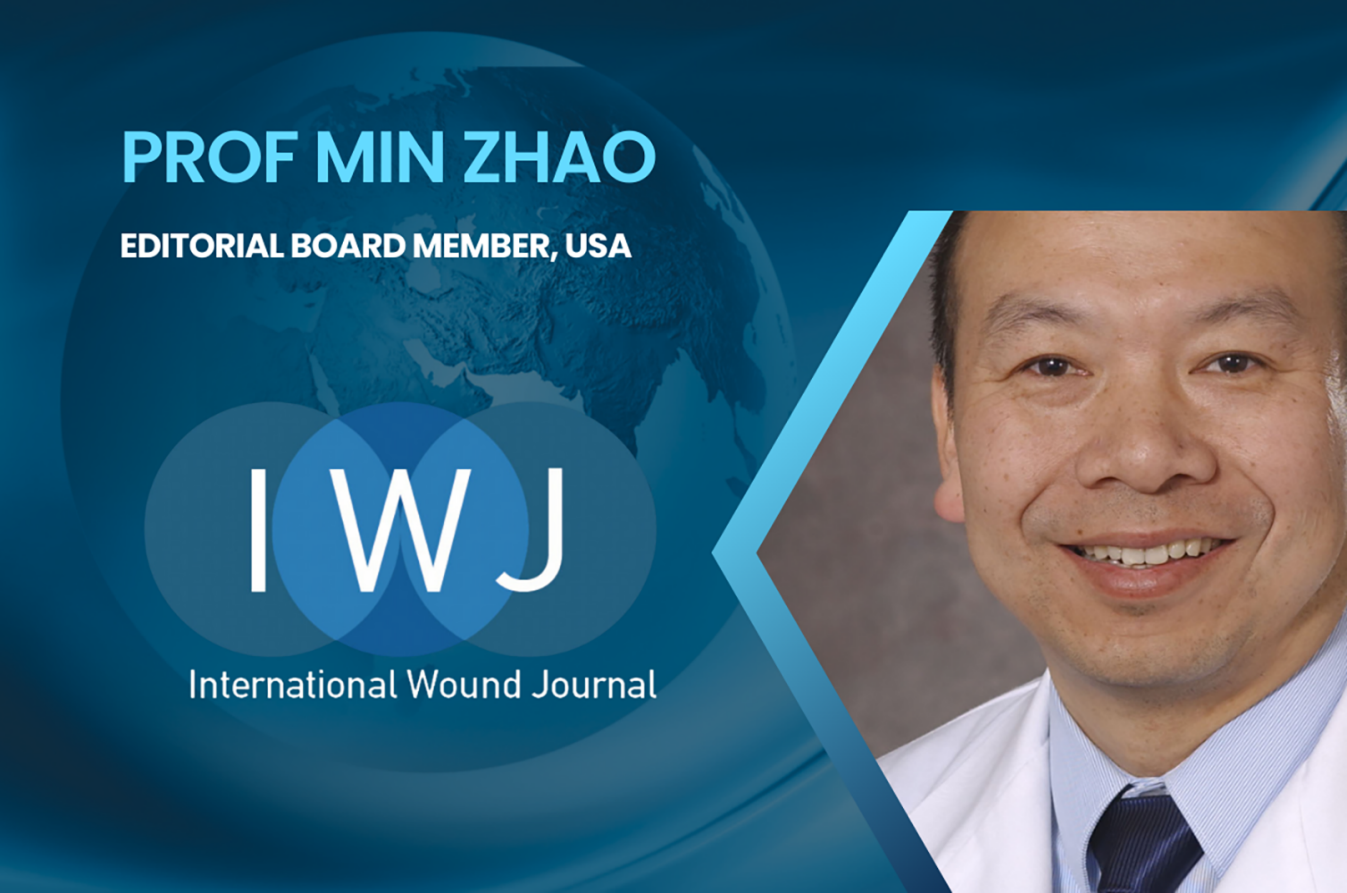

Dr. Zhao graduated from the 3rd Military Medical University in Chongqing, China and completed 1‐year clinical practice at the General Hospital in Beijing. He went on to complete his MSc and PhD in Trauma Surgery and Pathology at the Research Institute of Surgery under supervision of Professor Zhengguo WANG, academic and one of the founders of modern trauma surgery in China. He worked abroad as an honorary research fellow at University College London, England, UK, with Professor Geof Burnstock. He then moved to the University of Aberdeen, Scotland, UK, where he was awarded a prestigious Wellcome Trust University Award Lecturer and Senior Lecturer in 1999 and 2002, respectively, and to full professor in 2004. Dr. Zhao holds/ held honorary appointments as a visiting professor, faculty member and senior scientist with Johns Hopkins Medical School, UCSF, University of Toronto (Canada), Austrian Academy of Sciences (Vienna), The 3rd Military Medical University (China) and University of Aberdeen (UK).

**Dr Charles Zelen USA**

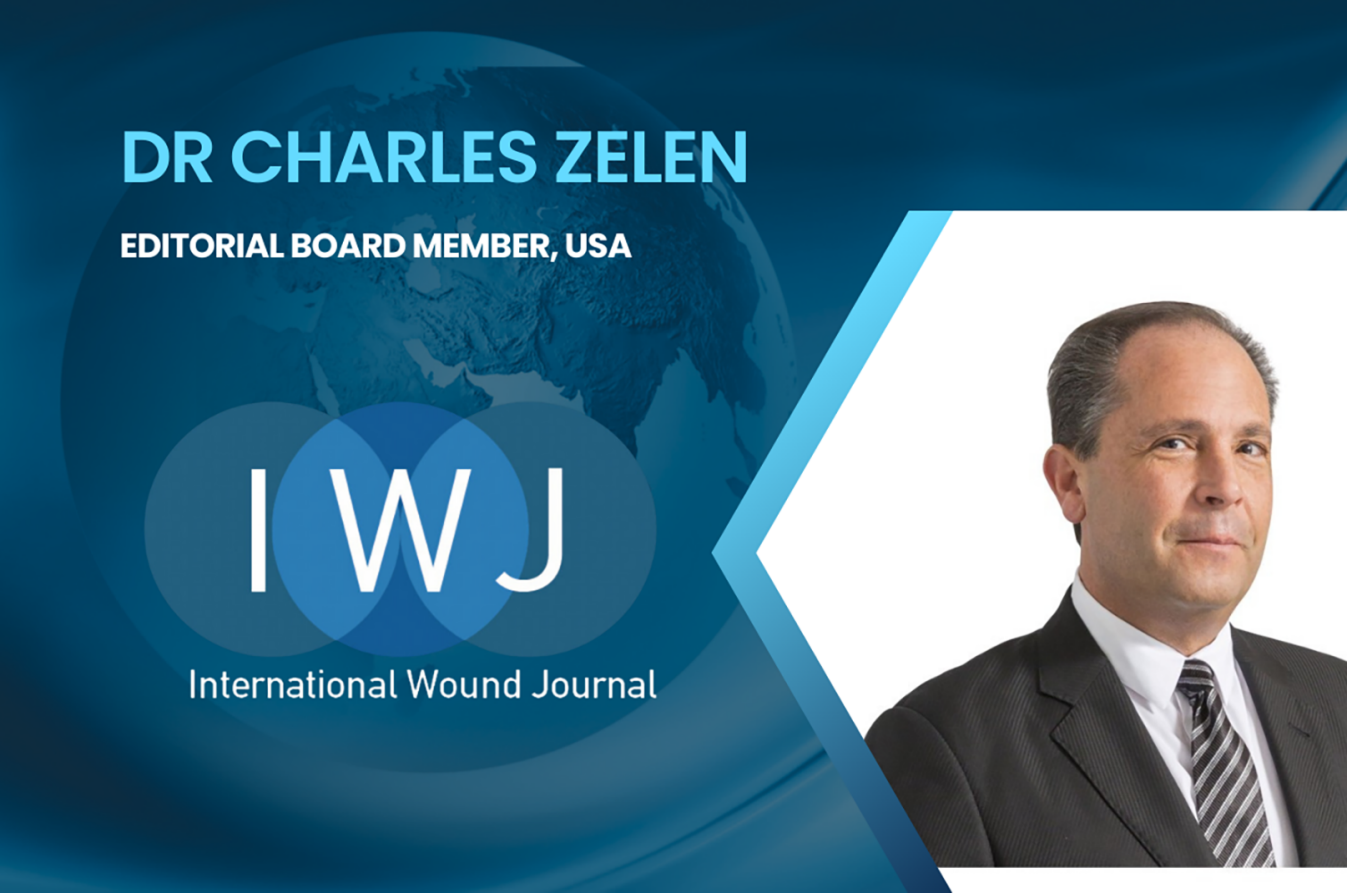

Dr. Charles Zelen, Founder and President of PERI, has over 20 years of clinical, academic and industry experience in extremities, wound care and biological development programmes. He has served as principal investigator and primary author on numerous wound healing randomized controlled trials. Dr. Zelen is Board Certified by the ABFAS. He received his BS in Psychobiology at the University of California at Riverside and his Doctor of Podiatric Medicine from the California School of Podiatric Medicine. He completed his residency at Inova Fairfax Hospital and received advanced training at Georgetown University Medical Center in Limb Salvage and Reconstruction. Dr. Zelen has volunteered with multiple charitable organizations, including organizations as far as Africa that bring children into the United States for Limb Salvage Surgery.

**Professor Karen Zulkowski, USA**

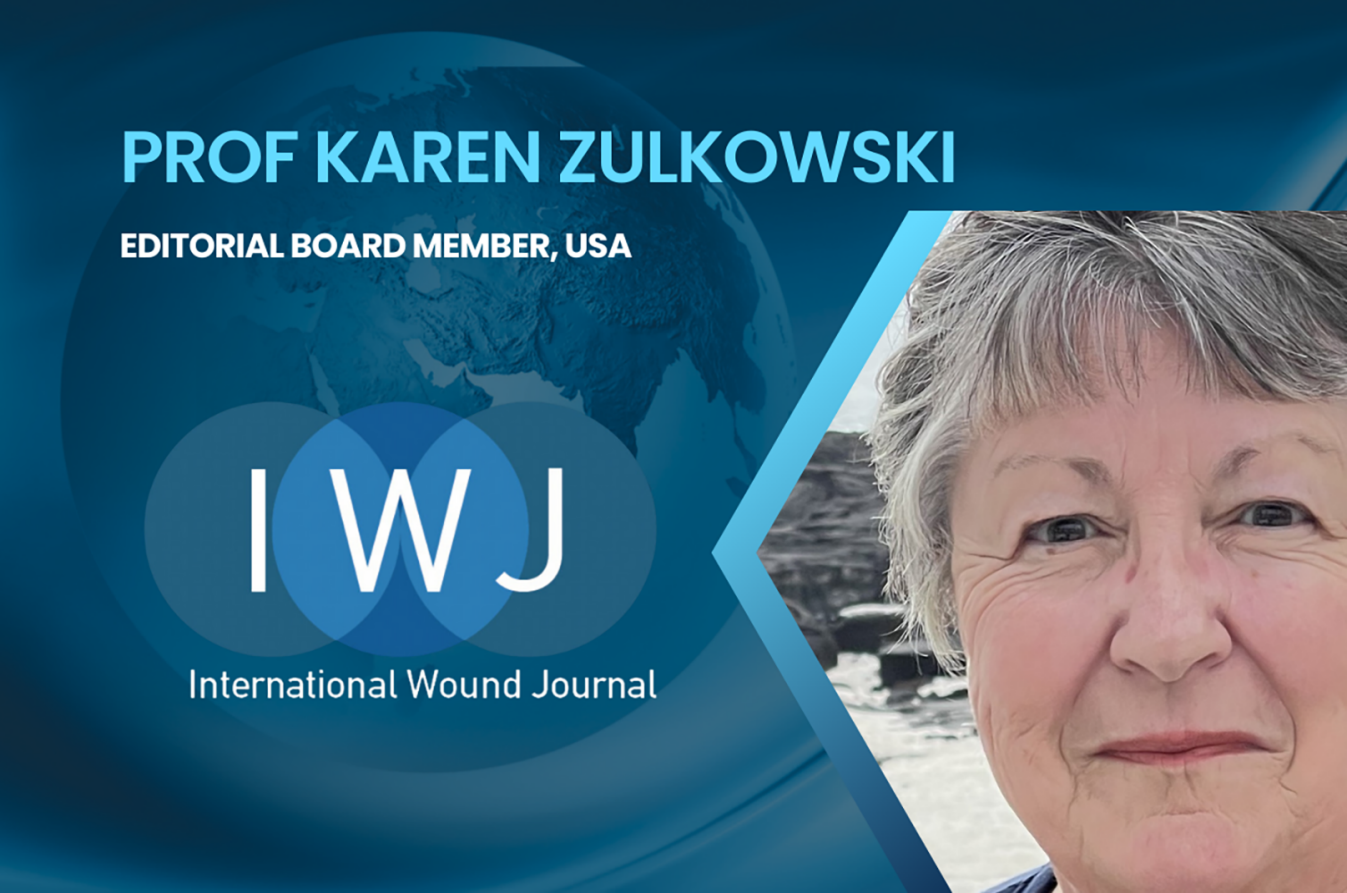

Dr Karen Zulkowski is a retired nursing professor. She is an internationally recognized wound specialist with extensive experience. Karen has consulted with AHRQ, CMS, the New Jersey Hospital Association and Mountain Pacific Quality Health. She was the editor and chief writer for the culturally appropriate based ostomy care and has been on national and international wound care boards as well as an HIH grant reviewer. Since moving to Kona, she has been on the Kona Community Hospital Foundation board and provided wound care to homeless in Oahu.

## References

[1] Harding K , Queen D . Expansion of our editorial board. Int Wound J. 2024;21:e70010. doi:10.1111/iwj.70010

